# Supplementary material for: Using a Chemical Genetic Screen to Enhance Our Understanding of the Antimicrobial Properties of Gallium against Escherichia coli
Source: Genes (Basel). 2019 Jan 9;10(1):34. doi: 10.3390/genes10010034 (PMC6356860; doi:10.3390/genes10010034)
Supplement: Supplementary file 1 [file genes-10-00034-s001.pdf]

Table S1. *Escherichia coli* gallium resistant and sensitive gene hits determined using a chemical genetic screen. Each individual score represents the mean of 9-12 trials.

ID	Name	Description	Score	P-value
JW0001	*thrA*	Bifunctional aspartokinase/homoserine dehydrogenase 1	0.0472	0.4974
JW0002	*thrB*	Homoserine kinase	0.4535	0.0103
JW0003	*thrC*	L-threonine synthase	0.0791	0.0185
JW0004	*yaaX*	DUF2502 family putative periplasmic protein	0.0959	0.0095
JW0005	*yaaA*	Peroxide resistance protein, lowers intracellular iron	0.0345	0.4262
JW0006	*yaaJ*	Putative transporter	0.0623	0.1141
JW0008	*mog*	Molybdochelatase incorporating molybdenum into molybdopterin	0.0269	0.1935
JW0009	*yaaH*	Succinate-acetate transporter	0.0876	0.0017
JW0010	*yaaW*	UPF0174 family protein	0.0694	0.2053
JW0012	*yaaI*	UPF0412 family protein	-0.0435	0.1445
JW0018	*nhaA*	Sodium-proton antiporter	-0.0091	0.7726
JW0019	*nhaR*	Transcriptional activator of nhaa	-0.0725	0.0417
JW0022	*rpsT*	30S ribosomal subunit protein S20	0.0020	0.9553
JW0024	*ileS*	Isoleucyl-trna synthetase	-0.1268	0.1086
JW0026	*fkpB*	FKBP-type peptidyl-prolyl cis-trans isomerase (rotamase)	0.0334	0.5347
JW0028	*rihC*	Ribonucleoside hydrolase 3	0.0069	0.9094
JW0030	*carA*	Carbamoyl phosphate synthetase small subunit, glutamine amidotransferase	0.0893	0.1038
JW0031	*carB*	Carbamoyl-phosphate synthase large subunit	0.0651	0.1219
JW0033	*caiF*	Cai operon transcriptional activator	0.1140	0.0898
JW0035	*caiD*	Carnitinyl-coa dehydratase	0.0250	0.5492
JW0037	*caiB*	Crotonobetainyl coa:carnitine coa transferase	0.0036	0.9101
JW0038	*caiA*	Crotonobetaine reductase subunit II, FAD-binding	-0.0047	0.9263
JW0039	*caiT*	Putative transporter	0.4032	0.0002
JW0040	*fixA*	Anaerobic carnitine reduction putative electron transfer flavoprotein subunit	0.0580	0.1061
JW0041	*fixB*	Putative electron transfer flavoprotein, NAD/FAD-binding domain and ETFP adenine nucleotide-binding domain-like protein	-0.0146	0.6336
JW0042	*fixC*	Putative oxidoreductase	-0.0065	0.8901
JW0043	*fixX*	Putative 4Fe-4S ferredoxin-type protein	0.0114	0.6153
JW0044	*yaaU*	Putative MFS sugar transporter; membrane protein	0.2370	0.0040
JW0045	*kefF*	Potassium-efflux system ancillary protein for kefc, glutathione-regulated; quinone oxidoreductase, FMN-dependent	0.0365	0.4077
JW0046	*kefC*	Potassium:proton antiporter	0.0821	0.1587
JW0048	*apaH*	Diadenosine tetraphosphatase	0.0322	0.4279

JW0049	*apaG*	Protein associated with Co2+ and Mg2+ efflux	0.0018	0.9319
JW0050	*ksgA*	16S rrna m(6)A1518, m(6)A1519 dimethyltransferase, SAM-dependent	0.0183	0.4308
JW0051	*pdxA*	4-hydroxy-L-threonine phosphate dehydrogenase, NAD-dependent	-0.0399	0.2976
JW0052	*surA*	Peptidyl-prolyl cis-trans isomerase (ppiase)	0.0615	0.0760
JW0054	*djlA*	Membrane-anchored dnak co-chaperone, DNA-binding protein	-0.0886	0.0213
JW0055	*yabP*	Pseudogene, pentapeptide repeats-containing	-0.0110	0.7437
JW0057	*rluA*	Dual specificity 23S rrna pseudouridine(746), trna pseudouridine(32) synthase, SAM-dependent	-0.0337	0.3005
JW0058	*hepA*	RNA polymerase remodeling/recycling factor atpase; RNA polymerase-associated, ATP-dependent RNA translocase	-0.0350	0.0106
JW0059	*polB*	DNA polymerase II	-0.1108	0.0259
JW0063	*araC*	Ara regulon transcriptional activator; autorepressor	-0.0071	0.6309
JW0065	*thiQ*	Thiamine/thiamine pyrophosphate ABC transporter atpase	-0.0016	0.9684
JW0066	*thiP*	Thiamine/thiamine pyrophosphate ABC transporter permease	-0.0074	0.7744
JW0067	*tbpA*	Thiamine/thiamine pyrophosphate/thiamine monophosphate ABC transporter periplasmic binding protein	-0.0807	0.0846
JW0068	*sgrR*	Transcriptional DNA-binding transcriptional activator of sgrs srna	0.0142	0.5347
JW0069	*setA*	Broad specificity sugar efflux system	0.1591	0.0062
JW0070	*leuD*	3-isopropylmalate dehydratase small subunit	0.1276	0.0071
JW0071	*leuC*	3-isopropylmalate dehydratase large subunit	0.2046	0.0029
JW0073	*leuA*	2-isopropylmalate synthase	0.3018	0.0007
JW0074	*leuL*	Leu operon leader peptide	0.1839	0.0154
JW0075	*leuO*	Global transcription factor	-0.0954	0.0058
JW0076	*ilvI*	Acetolactate synthase 3 large subunit	-0.0369	0.3710
JW0077	*ilvH*	Acetolactate synthase 3, small subunit, valine-sensitive	-0.0466	0.2660
JW0079	*mraZ*	Rsmh methytransferase inhibitor	0.0949	0.0169
JW0080	*mraW*	16S rrna m(4)C1402 methyltransferase, SAM-dependent	-0.0108	0.7186
JW0090	*ddlB*	D-alanine:D-alanine ligase	0.0217	0.6523
JW0097	*mutT*	Dgtp-preferring nucleoside triphosphate pyrophosphohydrolase	0.0637	0.0378
JW0099	*yacF*	Ftsz stabilizer	0.0369	0.3961
JW0100	*coaE*	Dephospho-coa kinase	-0.0713	0.2217
JW0101	*guaC*	GMP reductase	-0.0906	0.0906
JW0102	*hofC*	Assembly protein in type IV pilin biogenesis, transmembrane protein	0.0423	0.2413
JW0103	*hofB*	T2SE secretion family protein; P-loop atpase superfamily protein	0.1302	0.2282
JW0104	*ppdD*	Putative prepilin peptidase-dependent pilin	0.0307	0.2973

JW0105	*nadC*	Quinolinate phosphoribosyltransferase	0.0531	0.2658
JW0106	*ampD*	1,6-anhydro-N-acetylmuramyl-L-alanine amidase, Zn-dependent; murein amidase	-0.0325	0.2875
JW0107	*ampE*	Ampicillin resistance inner membrane protein; putative signaling protein in beta-lactamase regulation	0.0507	0.1490
JW0108	*aroP*	Aromatic amino acid transporter	-0.0040	0.9494
JW0109	*pdhR*	Pyruvate dehydrogenase complex repressor; autorepressor	0.3527	0.0006
JW0110	*aceE*	Pyruvate dehydrogenase, decarboxylase component E1, thiamine triphosphate-binding	0.0231	0.6135
JW0111	*aceF*	Pyruvate dehydrogenase, dihydrolipoyltransacetylase component E2	-0.0074	0.9198
JW0112	*lpd*	Dihydrolipoyl dehydrogenase; E3 component of pyruvate and 2-oxoglutarate dehydrogenases complexes; glycine cleavage system L protein; dihydrolipoamide dehydrogenase	0.0169	0.6226
JW0114	*acnB*	Aconitate hydratase 2; aconitase B; 2-methyl-cis-aconitate hydratase	0.0773	0.0493
JW0115	*yacL*	UPF0231 family protein	-0.0274	0.5653
JW0116	*speD*	S-adenosylmethionine decarboxylase	0.0504	0.1356
JW0117	*speE*	Spermidine synthase (putrescine aminopropyltransferase)	-0.0934	0.0296
JW0118	*yacC*	Puls_outs family protein	0.0396	0.0607
JW0119	*cueO*	Multicopper oxidase (laccase)	-0.0318	0.2142
JW0120	*gcd*	Glucose dehydrogenase	-0.0276	0.4399
JW0123	*yadG*	Putative ABC transporter atpase	-0.0423	0.1017
JW0124	*yadH*	Putative ABC transporter permease	-0.0854	0.0743
JW0125	*yadI*	Putative PTS Enzyme IIA	0.0623	0.1383
JW0126	*yadE*	Putative polysaccharide deacetylase lipoprotein	0.0522	0.0587
JW0127	*panD*	Aspartate 1-decarboxylase	-0.0540	0.3328
JW0129	*panC*	Pantothenate synthetase	0.0956	0.0027
JW0130	*panB*	3-methyl-2-oxobutanoate hydroxymethyltransferase	-0.0001	0.9988
JW0131	*yadC*	Putative fimbrial-like adhesin protein	-0.0385	0.2861
JW0132	*yadK*	Putative fimbrial-like adhesin protein	0.0591	0.0284
JW0133	*yadL*	Putative fimbrial-like adhesin protein	-0.0544	0.3251
JW0134	*yadM*	Putative fimbrial-like adhesin protein	-0.0475	0.1698
JW0135	*htrE*	Putative outer membrane usher protein	0.0886	0.0250
JW0136	*ecpD*	Putative periplasmic pilin chaperone	0.0361	0.0565
JW0137	*yadN*	Putative fimbrial-like adhesin protein	0.0044	0.9023
JW0141	*dksA*	Transcriptional regulator of rrna transcription; dnak suppressor protein	0.0751	0.1690
JW0142	*sfsA*	Sugar fermentation stimulation protein A	0.0127	0.7298
JW0144	*hrpB*	Putative ATP-dependent helicase	-0.0254	0.2078
JW0145	*mrcB*	Fused glycosyl transferase and transpeptidase	0.2493	0.0002

JW0147	*fhuC*	Iron(3+)-hydroxamate import ABC transporter atpase	0.0110	0.7885
JW0148	*fhuD*	Iron(3+)-hydroxamate import ABC transporter periplasmic binding protein	-0.0405	0.6255
JW0149	*fhuB*	Iron(3+)-hydroxamate import ABC transporter permease	0.0109	0.8317
JW0153	*yadS*	UPF0126 family inner membrane protein	0.0828	0.0008
JW0154	*btuF*	Vitamin B12 ABC transporter periplasmic binding protein	0.0151	0.5763
JW0155	*pfs*	5'-methylthioadenosine/S-adenosylhomocysteine nucleosidase	-0.3293	0.0001
JW0156	*dgt*	Deoxyguanosine triphosphate triphosphohydrolase	0.0022	0.9574
JW0157	*degP*	Serine endoprotease (protease Do), membrane-associated	0.0039	0.9025
JW0159	*yaeH*	UPF0325 family protein	0.0036	0.9317
JW0162	*glnD*	Uridylyltransferase	-0.0336	0.2002
JW0173	*hlpA*	Periplasmic chaperone	0.0900	0.0279
JW0178	*rnhB*	Ribonuclease HII, degrades RNA of DNA-RNA hybrids	0.0321	0.2498
JW0181	*ldcC*	Lysine decarboxylase 2, constitutive	-0.0490	0.2259
JW0182	*yaeR*	Putative lyase	-0.0432	0.3720
JW0185	*yaeP*	UPF0253 family protein	0.0589	0.0006
JW0186	*yaeQ*	PDDEXK superfamily protein	0.0076	0.8627
JW0187	*yaeJ*	Alternative stalled-ribosome rescue factor B; peptidyl-trna hydrolase, ribosome-attached	0.0106	0.8532
JW0188	*nlpE*	Lipoprotein involved with copper homeostasis and adhesion	-0.0371	0.3837
JW0191	*yaeB*	Trna-Thr(GGU) m(6)t(6)A37 methyltransferase, SAM-dependent	-0.0365	0.3118
JW0192	*rcsF*	Putative outer membrane protein	0.0082	0.7331
JW0193	*metQ*	DL-methionine transporter subunit	0.0465	0.1112
JW0194	*metI*	DL-methionine transporter subunit	-0.0544	0.2145
JW0195	*metN*	DL-methionine transporter subunit	0.0282	0.4134
JW0196	*gmhB*	D,D-heptose 1,7-bisphosphate phosphatase	-0.0387	0.2674
JW0197	*dkgB*	2,5-diketo-D-gluconate reductase B	-0.0265	0.4403
JW0198	*yafC*	Lysr family putative transcriptional regulator	-0.0307	0.5736
JW0200	*yafE*	Putative S-adenosyl-L-methionine-dependent methyltransferase	-0.0890	0.1394
JW0202	*gloB*	Hydroxyacylglutathione hydrolase	-0.2974	0.0001
JW0203	*yafS*	Putative S-adenosyl-L-methionine-dependent methyltransferase	0.0639	0.0796
JW0204	*rnhA*	Ribonuclease HI, degrades RNA of DNA-RNA hybrids	-0.0779	0.0007
JW0205	*dnaQ*	DNA polymerase III epsilon subunit	0.0716	0.1749
JW0206	*yafT*	Lipoprotein	-0.0496	0.3501
JW0207	*yafU*	Pseudogene	0.2145	0.0000
JW0210	*ivy*	Inhibitor of c-type lysozyme, periplasmic	0.0009	0.0055

JW0212	*lpcA*	D-sedoheptulose 7-phosphate isomerase	-0.0396	0.3807
JW0213	*yafJ*	Type 2 glutamine amidotransferase family protein	-0.0893	0.0214
JW0214	*yafK*	L,D-transpeptidase-related protein	0.0021	0.9528
JW0215	*yafQ*	Mrna interferase toxin of toxin-antitoxin pair yafq/dinj	-0.0373	0.3662
JW0216	*dinJ*	Antitoxin of yafq-dinj toxin-antitoxin system	-0.0170	0.7214
JW0217	*yafL*	Putative lipoprotein and C40 family peptidase	0.0532	0.3486
JW0218	*yafM*	RAYT REP element-mobilizing transposase; tnpa(REP)	-0.1104	0.0034
JW0221	*dinB*	DNA polymerase IV	-0.0320	0.0918
JW0222	*yafN*	Antitoxin of the yafo-yafn toxin-antitoxin system	-0.0030	0.9392
JW0223	*yafO*	Mrna interferase toxin of the yafo-yafn toxin-antitoxin system	0.0863	0.1191
JW0224	*yafP*	GNAT family putative N-acetyltransferase	0.0498	0.1897
JW0225	*ykfJ*	Pseudogene	0.0269	0.5742
JW0226	*prfH*	Putative peptide chain release factor homolog	0.1016	0.0030
JW0227	*pepD*	Aminoacyl-histidine dipeptidase (peptidase D)	0.0164	0.4127
JW0228	*gpt*	Xanthine phosphoribosyltransferase; xanthine-guanine phosphoribosyltransferase	0.0414	0.1513
JW0229	*frsA*	Fermentation-respiration switch protein; PTS Enzyme IIA(Glc)-binding protein; pnp-butyrate esterase activity	0.0000	0.8994
JW0230	*crl*	Pseudogene, sigma factor-binding protein, RNA polymerase holoenzyme formation stimulator	-0.0096	0.7782
JW0231	*phoE*	Outer membrane phosphoporin protein E	0.1049	0.0225
JW0232	*proB*	Gamma-glutamate kinase	0.2582	0.0017
JW0233	*proA*	Gamma-glutamylphosphate reductase	0.1610	0.0047
JW0234	*ykfI*	CP4-6 prophage; toxin of the ykfi-yafw toxin-antitoxin system	0.0490	0.0914
JW0235	*yafW*	CP4-6 prophage; antitoxin of the ykfi-yafw toxin-antitoxin system	0.0523	0.1142
JW0236	*ykfG*	CP4-6 prophage; radc-like JAB domain protein	0.0187	0.4978
JW0239	*ykfB*	CP4-6 prophage; uncharacterized protein	0.1860	0.1122
JW0242	*yafZ*	CP4-6 prophage; conserved protein	-0.0039	0.8677
JW0243	*ykfA*	CP4-6 prophage; putative GTP-binding protein	0.0559	0.1359
JW0254	*afuC*	CP4-6 prophage; putative ferric transporter subunit	-0.0989	0.0015
JW0258	*ykgN*	Putative transposase ykgn	0.0367	0.5541
JW0259	*yagB*	Pseudogene, CP4-6 prophage	-0.0165	0.8693
JW0261	*yagE*	2-keto-3-deoxy gluconate (KDG) aldolase; CP4-6 prophage	-0.0313	0.4052
JW0262	*yagF*	D-xylonate dehydratase yagf	0.0062	0.8250
JW0264	*yagH*	CP4-6 prophage; putative xylosidase/arabinosidase	-0.0046	0.8036
JW0265	*yagI*	CP4-6 prophage; putative DNA-binding transcriptional regulator	-0.0389	0.1147

JW0266	*argF*	Ornithine carbamoyltransferase 2, chain F; CP4-6 prophage	-0.0267	0.7595
JW0270	*yagJ*	CP4-6 prophage; uncharacterized protein	0.1469	0.0610
JW0271	*yagK*	CP4-6 prophage; conserved protein	0.0009	0.9845
JW0272	*yagL*	CP4-6 prophage; DNA-binding protein	0.0240	0.2312
JW0273	*yagM*	CP4-6 prophage; uncharacterized protein	-0.0790	0.2877
JW0274	*yagN*	Uncharacterized protein	0.0006	0.0001
JW0275	*intF*	CP4-6 prophage; putative phage integrase	0.0047	0.9030
JW0276	*yagP*	Pseudogene, lysr family, fragment	-0.0518	0.0456
JW0277	*yagQ*	Moco insertion factor for paoabc aldehyde oxidoreductase	0.0510	0.2110
JW0278	*yagR*	Paoabc aldehyde oxidoreductase, Moco-containing subunit	-0.0699	0.0961
JW0279	*yagS*	Paoabc aldehyde oxidoreductase, FAD-containing subunit	-0.0157	0.5515
JW0280	*yagT*	Paoabc aldehyde oxidoreductase, 2Fe-2S subunit	-0.0531	0.1353
JW0282	*ykgJ*	UPF0153 cysteine cluster protein	-0.3536	0.0520
JW0284	*yagW*	Polymerized tip adhesin of ECP fibers	0.0478	0.6753
JW0285	*yagX*	ECP production outer membrane protein	0.0308	0.3952
JW0287	*yagZ*	ECP pilin	-0.0234	0.8509
JW0291	*eaeH*	Pseudogene, attaching and effacing protein homology	0.0683	0.1652
JW0298	*ykgD*	Reactive chlorine species (RCS)-specific activator of the rcl genes	-0.0285	0.2830
JW0300	*ykgF*	Ferridoxin-like lutb family protein; putative electron transport chain ykgefg component	0.0579	0.0816
JW0302	*ykgH*	Putative inner membrane protein	-0.1279	0.0094
JW0303	*betA*	Choline dehydrogenase, a flavoprotein	-0.0105	0.8513
JW0304	*betB*	NAD/NADP-dependent betaine aldehyde dehydrogenase	-0.0312	0.3590
JW0306	*betT*	Choline transporter of high affinity	0.0556	0.1489
JW0307	*yahA*	C-di-GMP-specific phosphodiesterase	0.0379	0.2407
JW0308	*yahB*	Putative DNA-bindng transcriptional regulator	0.0222	0.5778
JW0309	*yahC*	Putative inner membrane protein	-0.0527	0.0506
JW0310	*yahD*	Ankyrin repeat protein	0.0773	0.0036
JW0311	*yahE*	DUF2877 family protein	0.0920	0.2564
JW0312	*yahF*	Putative NAD(P)-binding succinyl-coa synthase	0.1872	0.0094
JW0313	*yahG*	DUF1116 family protein	0.0166	0.6710
JW0315	*yahI*	Carbamate kinase-like protein	-0.0160	0.4839
JW0316	*yahJ*	Putative metallo-dependent hydrolase domain deaminase	0.0370	0.2233
JW0317	*yahK*	Broad specificity NADPH-dependent aldehyde reductase, Zn-containing	-0.0434	0.3082
JW0318	*yahL*	Uncharacterized protein	-0.0215	0.6557
JW0320	*yahN*	Amino acid exporter for proline, lysine, glutamate, homoserine	0.0146	0.5737

JW0321	*yahO*	Periplasmic protein, function unknown, yhcn family	-0.0075	0.6990
JW0322	*prpR*	Propionate catabolism operon regulatory protein	-0.0770	0.0254
JW0323	*prpB*	2-methylisocitrate lyase	0.0842	0.0173
JW0325	*prpD*	2-methylcitrate dehydratase	-0.0991	0.0151
JW0326	*prpE*	Propionate--coa ligase	-0.0150	0.6642
JW0328	*codA*	Cytosine/isoguanine deaminase	0.0435	0.0912
JW0330	*cynT*	Carbonic anhydrase	-0.1279	0.0397
JW0331	*cynS*	Cyanate aminohydrolase	-0.0099	0.8465
JW0333	*lacA*	Thiogalactoside acetyltransferase	0.0013	0.9752
JW0334	*lacY*	Lactose permease	0.0001	0.3369
JW0336	*lacI*	Lactose-inducible lac operon transcriptional repressor	-0.0136	0.8200
JW0338	*mhpA*	3-(3-hydroxyphenyl)propionate hydroxylase	-0.0018	0.9629
JW0339	*mhpB*	2,3-dihydroxyphenylpropionate 1,2-dioxygenase	-0.0507	0.2461
JW0340	*mhpC*	2-hydroxy-6-ketonona-2,4-dienedioic acid hydrolase	-0.0350	0.4862
JW0341	*mhpD*	2-keto-4-pentenoate hydratase	0.0175	0.7038
JW0342	*mhpF*	Acetaldehyde-coa dehydrogenase II, NAD-binding	-0.0358	0.2176
JW0343	*mhpE*	4-hyroxy-2-oxovalerate/4-hydroxy-2-oxopentanoic acid aldolase, class I	0.0574	0.3375
JW0345	*yaiL*	DUF2058 family protein	-0.0370	0.4138
JW0346	*frmB*	S-formylglutathione hydrolase	-0.0195	0.6300
JW0347	*frmA*	Alcohol dehydrogenase class III; glutathione-dependent formaldehyde dehydrogenase	-0.0349	0.2536
JW0348	*frmR*	Regulator protein that represses frmrab operon	0.0589	0.0482
JW0349	*yaiO*	Outer membrane protein	0.0184	0.8961
JW0350	*yaiX*	CP4-44 prophage; putative disrupted hemin or colicin receptor	-0.0538	0.3747
JW0355	*yaiP*	Putative family 2 glycosyltransferase	0.2771	0.0620
JW0356	*yaiS*	Putative PIG-L family deacetylase	-0.0638	0.4803
JW0357	*tauA*	Taurine ABC transporter periplasmic binding protein	0.0177	0.5973
JW0359	*tauC*	Taurine ABC transporter permease	-0.0271	0.6778
JW0360	*tauD*	Taurine dioxygenase, 2-oxoglutarate-dependent	0.0687	0.1301
JW0362	*yaiT*	CP4-44 prophage; putative disrupted hemin or colicin receptor	0.0715	0.0112
JW0368	*sbmA*	Peptide antibiotic transporter	-0.0169	0.6344
JW0370	*yaiY*	DUF2755 family inner membrane protein	0.1216	0.0233
JW0372	*ddlA*	D-alanine-D-alanine ligase A	0.0382	0.2436
JW0373	*yaiB*	Anti-rssb factor, rpos stabilzer during Pi starvation; anti-adapter protein	0.0189	0.5881
JW0374	*phoA*	Bacterial alkaline phosphatase	0.0698	0.1355
JW0376	*yaiC*	Diguanylate cyclase, cellulose regualtor	0.0130	0.4483
JW0377	*proC*	Pyrroline-5-carboxylate reductase, NAD(P)-binding	0.0135	0.6743

JW0378	*yaiI*	UPF0178 family protein	0.0690	0.1073
JW0379	*aroL*	Shikimate kinase II	0.0415	0.3263
JW0380	*yaiA*	Oxyr-regulated conserved protein	0.1142	0.0673
JW0381	*aroM*	Arom family protein	0.5247	0.0006
JW0382	*yaiE*	Pyrimidine/purine nucleoside phosphorylase	0.2110	0.1711
JW0383	*ykiA*	Pseudogene	-0.0832	0.0849
JW0384	*rdgC*	Recombination-associated protein rdgc	-0.0091	0.7698
JW0385	*mak*	Manno(fructo)kinase	0.0046	0.9371
JW0386	*araJ*	L-arabinose-inducible putative transporter, MFS family	-0.1904	0.0283
JW0387	*sbcC*	Exonuclease, dsdna, ATP-dependent	0.1393	0.2692
JW0388	*sbcD*	Exonuclease, dsdna, ATP-dependent	0.0375	0.2520
JW0389	*phoB*	Response regulator in two-component regulatory system with phor	-0.0032	0.9378
JW0390	*phoR*	Sensory histidine kinase in two-component regulatory system with phob	-0.0052	0.8876
JW0391	*brnQ*	Branched-chain amino acid transport system 2 carrier protein; LIV-II transport system for Ile, Leu, and Val	-0.0934	0.0150
JW0393	*malZ*	Maltodextrin glucosidase	0.0606	0.3683
JW0394	*yajB*	Acyl carrier protein (ACP) phosphodiesterase; ACP hydrolyase	0.0259	0.8446
JW0395	*queA*	S-adenosylmethionine:trna ribosyltransferase-isomerase	0.0056	0.8924
JW0396	*tgt*	Trna-guanine transglycosylase	-0.0425	0.1912
JW0397	*yajC*	Secyeg protein translocase auxillary subunit	0.0564	0.0178
JW0403	*ybaD*	Nrd regulon repressor	0.0533	0.6363
JW0406	*nusB*	Transcription antitermination protein	0.0588	0.0599
JW0408	*pgpA*	Phosphatidylglycerophosphatase A	-0.0640	0.3239
JW0409	*yajO*	2-carboxybenzaldehyde reductase	-0.0075	0.7981
JW0412	*xseB*	Exonuclease VII small subunit	-0.0197	0.7122
JW0413	*thiI*	Trna s(4)U8 sulfurtransferase	0.1320	0.1927
JW0415	*panE*	2-dehydropantoate reductase, NADPH-specific	-0.1197	0.0059
JW0418	*cyoE*	Protoheme IX farnesyltransferase	-0.0200	0.5023
JW0419	*cyoD*	Cytochrome o ubiquinol oxidase subunit IV	-0.1968	0.2691
JW0420	*cyoC*	Cytochrome o ubiquinol oxidase subunit III	0.0591	0.2626
JW0421	*cyoB*	Cytochrome o ubiquinol oxidase subunit I	0.1037	0.0163
JW0423	*ampG*	Muropeptide transporter	-0.0017	0.9576
JW0424	*yajG*	Putative lipoprotein	-0.0601	0.1249
JW0426	*tig*	Peptidyl-prolyl cis/trans isomerase (trigger factor)	0.0212	0.3959
JW0427	*clpP*	Proteolytic subunit of clpa-clpp and clpx-clpp ATP-dependent serine proteases	0.0177	0.6833
JW0428	*clpX*	Atpase and specificity subunit of clpx-clpp ATP-dependent serine protease	0.1388	0.0371
JW0430	*hupB*	HU, DNA-binding transcriptional regulator, beta subunit	0.1564	0.0219

JW0431	*ppiD*	Periplasmic folding chaperone, has an inactive ppiase domain	0.0004	0.9896
JW0432	*ybaV*	Putative competence-suppressing periplasmic helix-hairpin-helix DNA-binding protein	-0.0038	0.8970
JW0433	*ybaW*	Long-chain acyl-coa thioesterase III	0.0120	0.8238
JW0434	*ybaX*	7-cyano-7-deazaguanine (preq0) synthase; queuosine biosynthesis	-0.0300	0.2253
JW0435	*ybaE*	Putative ABC transporter periplasmic binding protein	-0.0291	0.5749
JW0437	*ybaO*	Putative DNA-binding transcriptional regulator	-0.0174	0.6375
JW0438	*mdlA*	Putative multidrug ABC transporter atpase	0.0208	0.6008
JW0440	*glnK*	Nitrogen assimilation regulatory protein for glnl, glne, and amtb	0.0153	0.4342
JW0441	*amtB*	Ammonium transporter	0.0447	0.3070
JW0442	*tesB*	Acyl-coa thioesterase 2	0.0952	0.0193
JW0443	*ybaY*	Outer membrane lipoprotein	0.0293	0.3735
JW0444	*ybaZ*	Excision repair protein, alkyltransferase-like protein ATL	-0.1142	0.0008
JW0445	*ybaA*	DUF1428 family protein	-0.0814	0.0517
JW0448	*maa*	Maltose O-acetyltransferase	0.0258	0.5687
JW0449	*hha*	Modulator of gene expression, with H-NS	0.0087	0.7809
JW0450	*ybaJ*	Hha toxicity attenuator; conjugation-related protein	0.0342	0.2269
JW0451	*acrB*	Multidrug efflux system protein	0.0802	0.0227
JW0452	*acrA*	Multidrug efflux system	-0.0551	0.1819
JW0453	*acrR*	Transcriptional repressor	0.0461	0.3424
JW0454	*kefA*	Mechanosensitive channel protein, intermediate conductance, K+ regulated	-0.2730	0.0881
JW0456	*priC*	Primosomal replication protein N''	0.1440	0.0311
JW0457	*ybaN*	DUF454 family inner membrane protein	-0.0989	0.4170
JW0458	*apt*	Adenine phosphoribosyltransferase	0.0259	0.3409
JW0460	*ybaB*	DNA-binding protein, putative nucleoid-associated protein	-0.0422	0.2370
JW0461	*recR*	Gap repair protein	-0.0360	0.5634
JW0462	*htpG*	Protein refolding molecular co-chaperone Hsp90, Hsp70-dependent; heat-shock protein; atpase	-0.0186	0.4757
JW0465	*aes*	Acetyl esterase	0.0060	0.8555
JW0466	*gsk*	Inosine/guanosine kinase	-0.0357	0.3419
JW0467	*ybaL*	Inner membrane putative NAD(P)-binding transporter	-0.0354	0.1149
JW0468	*fsr*	Putative fosmidomycin efflux system protein	0.0266	0.3010
JW0469	*ushA*	Bifunctional UDP-sugar hydrolase/5'-nucleotidase	-0.0251	0.5861
JW0470	*ybaK*	Cys-trna(Pro)/Cys-trna(Cys) deacylase	-0.0696	0.0746
JW0471	*ybaP*	Trab family protein	0.0240	0.7053
JW0472	*ybaQ*	Putative DNA-binding transcriptional regulator	0.0480	0.0600
JW0474	*ybaS*	Glutaminase 1	-0.0429	0.1848

JW0476	*cueR*	Copper-responsive regulon transcriptional regulator	-0.0007	0.9813
JW0478	*ybbK*	PHB domain membrane-anchored putative protease	0.1422	0.0003
JW0479	*ybbL*	Iron export ABC transporter atpase; peroxide resistance protein	-0.0732	0.2073
JW0482	*ybbO*	Short-chain dehydrogenases/reductases (SDR) family protein	-0.0627	0.0937
JW0483	*tesA*	Acyl-coa thioesterase 1 and protease I and lysophospholipase L1	-0.1179	0.0065
JW0485	*ybbP*	Putative ABC transporter permease	0.0037	0.8293
JW0487	*ybbC*	Putative immunity protein	0.0556	0.1033
JW0488	*ylbH*	Pseudogene, Rhs family C-terminal fragment with unique putative toxin domain	-0.0179	0.2883
JW0489	*ybbD*	Pseudogene	0.0507	0.2029
JW0491	*ybbB*	Trna 2-selenouridine synthase, selenophosphate-dependent	-0.0477	0.0472
JW0492	*ybbS*	Alld operon transcriptional activator	-0.0998	0.0063
JW0493	*allA*	Ureidoglycolate lyase, releasing urea	0.0211	0.3841
JW0494	*allR*	Glyoxylate-inducible transcriptional repressor of all and gcl operons	-0.0093	0.8149
JW0495	*gcl*	Glyoxylate carboligase	-0.0609	0.2334
JW0497	*glxR*	Tartronate semialdehyde reductase, NADH-dependent	-0.0103	0.7936
JW0498	*ybbV*	Uncharacterized protein	0.0005	0.0017
JW0499	*ybbW*	Putative allantoin transporter	0.0442	0.1522
JW0500	*allB*	Allantoinase	-0.0053	0.8667
JW0501	*ybbY*	Putative uracil/xanthine transporter	-0.0152	0.5182
JW0502	*glxK*	Glycerate kinase II	-0.0077	0.9058
JW0504	*allC*	Allantoate amidohydrolase	0.0047	0.8780
JW0505	*allD*	Ureidoglycolate dehydrogenase	-0.0190	0.7082
JW0506	*fdrA*	Putative NAD(P)-binding acyl-coa synthetase	0.4180	0.0365
JW0507	*ylbE*	CP4-44 prophage; putative disrupted hemin or colicin receptor	0.0341	0.4445
JW0510	*ybcF*	Putative carbonate kinase	-0.0142	0.4673
JW0511	*purK*	N5-carboxyaminoimidazole ribonucleotide synthase	0.0805	0.0075
JW0512	*purE*	N5-carboxyaminoimidazole ribonucleotide mutase	-0.0488	0.1404
JW0516	*ybcI*	DUF457 family inner membrane protein	0.0084	0.9143
JW0519	*sfmA*	Fima homolog, function unknown	-0.0177	0.6092
JW0521	*sfmD*	Putative outer membrane export usher protein	-0.0324	0.0180
JW0525	*intD*	DLP12 prophage; putative phage integrase	0.0418	0.1616
JW0526	*ybcC*	Pseudogene, DLP12 prophage; phage-type exonuclease family	0.0498	0.2276
JW0527	*ybcD*	CP4-44 prophage; putative disrupted hemin or colicin receptor	-0.0108	0.7997
JW0530	*renD*	Pseudogene, DLP12 prophage	-0.0345	0.1919

JW0531	*emrE*	DLP12 prophage; multidrug resistance protein	-0.0148	0.6497
JW0532	*ybcK*	DLP12 prophage; putative phage recombinase/integrase	-0.0022	0.9614
JW0533	*ybcL*	DLP12 prophage; inactive polymorphonuclear leukocyte migration suppressor; UPF0098 family secreted protein	0.0731	0.2792
JW0534	*ybcM*	DLP12 prophage; putative DNA-binding transcriptional regulator	-0.0278	0.5405
JW0535	*ybcN*	DLP12 prophage; SSB and ssdna binding protein; putative recombination protein	-0.1333	0.3909
JW0536	*ninE*	DLP12 prophage; conserved protein	-0.0130	0.7280
JW0537	*ybcO*	DLP12 prophage; DUF1364 family protein	-0.1306	0.4046
JW0539	*ybcQ*	DLP12 prophage; putative antitermination protein	0.0528	0.2645
JW0543	*essD*	DLP12 prophage; putative phage lysis protein	-0.0694	0.0325
JW0544	*ybcS*	DLP12 prophage; putative lysozyme	0.0278	0.5158
JW0546	*borD*	DLP12 prophage; putative lipoprotein	0.0308	0.4546
JW0548	*ybcW*	DLP12 prophage; uncharacterized protein	0.0086	0.7936
JW0549	*nohB*	DLP12 prophage; DNA packaging protein	-0.0564	0.1763
JW0551	*ybcY*	Pseudogene, DLP12 prophage; methyltransferase homology	0.0215	0.6260
JW0552	*ylcE*	Pseudogene, DLP12 prophage	-0.0619	0.1824
JW0553	*appY*	Global transcriptional activator; DLP12 prophage	-0.1109	0.0045
JW0554	*ompT*	DLP12 prophage; outer membrane protease VII; outer membrane protein 3b	0.0259	0.5577
JW0555	*envY*	Porin thermoregulatory transcriptional activator	0.1473	0.0304
JW0556	*ybcH*	PRK09936 family protein	0.1071	0.3901
JW0557	*nfrA*	Bacteriophage N4 receptor, outer membrane subunit	0.0609	0.0445
JW0558	*nfrB*	Bacteriophage N4 receptor, inner membrane subunit	-0.0262	0.5743
JW0561	*cusC*	Copper/silver efflux system, outer membrane component	0.0025	0.9092
JW0563	*cusB*	Copper/silver efflux system, membrane fusion protein	0.0526	0.1601
JW0564	*cusA*	Copper/silver efflux system, membrane component	0.0226	0.5199
JW0565	*pheP*	Phenylalanine transporter	-0.0111	0.7506
JW0566	*ybdG*	Mechanosensitive channel protein, miniconductance	-0.0461	0.4129
JW0567	*nfnB*	Dihydropteridine reductase, NAD(P)H-dependent, oxygen-insensitive	-0.0927	0.0224
JW0569	*ybdJ*	DUF1158 family putative inner membrane protein	0.0454	0.5670
JW0570	*ybdK*	Weak gamma-glutamyl:cysteine ligase	0.0057	0.8625
JW0576	*fes*	Enterobactin/ferrienterobactin esterase	0.2530	0.0488
JW0577	*ybdZ*	Stimulator of entf adenylation activity, mbth-like	-0.2336	0.0174
JW0578	*entF*	Enterobactin synthase multienzyme complex component, ATP-dependent	-0.0275	0.5968

JW0579	*fepE*	Regulator of length of O-antigen component of lipopolysaccharide chains	-0.0466	0.3297
JW0580	*fepC*	Ferrienterobactin ABC transporter atpase	-0.0386	0.7192
JW0581	*fepG*	Iron-enterobactin ABC transporter permease	0.3122	0.0027
JW0582	*fepD*	Ferrienterobactin ABC transporter permease	-0.0132	0.6283
JW0583	*ybdA*	Enterobactin exporter, iron-regulated	-0.1520	0.0109
JW0584	*fepB*	Ferrienterobactin ABC transporter periplasmic binding protein	0.0048	0.9616
JW0585	*entC*	Isochorismate synthase 1	-0.0851	0.0304
JW0586	*entE*	2,3-dihydroxybenzoate-AMP ligase component of enterobactin synthase multienzyme complex	0.0350	0.1471
JW0587	*entB*	Isochorismatase	-0.0869	0.2346
JW0588	*entA*	2,3-dihydro-2,3-dihydroxybenzoate dehydrogenase	-0.0692	0.0264
JW0589	*ybdB*	Enterobactin synthesis proofreading thioesterase	-0.0149	0.6590
JW0590	*cstA*	Carbon starvation protein involved in peptide utilization; APC peptide transporter family protein	-0.0234	0.6816
JW0591	*ybdD*	DUF466 family protein	0.0313	0.5646
JW0592	*ybdH*	Hydroxycarboxylate dehydrogenase A	-0.0250	0.5675
JW0594	*ybdM*	Spo0J family protein, parb-like nuclease domain	0.0180	0.4605
JW0595	*ybdN*	PAPS reductase-like domain protein	-0.1102	0.4590
JW0596	*ybdO*	Putative DNA-binding transcriptional regulator	-0.2785	0.0558
JW0597	*dsbG*	Thiol:disulfide interchange protein, periplasmic	-0.0494	0.0846
JW0600	*uspG*	Universal stress protein UP12	0.0837	0.0967
JW0601	*ybdR*	Uncharacterized zinc-type alcohol dehydrogenase-like protein	-0.0745	0.0715
JW0602	*rnk*	Regulator of nucleoside diphosphate kinase	0.0436	0.3543
JW0603	*rna*	Ribonuclease I	-0.0113	0.6756
JW0604	*citT*	Citrate/succinate antiporter; citrate carrier	-0.0265	0.5467
JW0605	*citG*	2-(5''-triphosphoribosyl)-3'-dephosphocoenzyme-A synthase	0.0256	0.7108
JW0606	*citX*	Apo-citrate lyase phosphoribosyl-dephospho-coa transferase	-0.0694	0.1744
JW0608	*citE*	Citrate lyase, citryl-ACP lyase (beta) subunit	-0.0231	0.5381
JW0609	*citD*	Citrate lyase, acyl carrier (gamma) subunit	-0.0544	0.1867
JW0610	*citC*	Citrate lyase ligase; [citrate [pro-3S]-lyase] ligase	-0.0898	0.0081
JW0611	*citA*	Sensory histidine kinase in two-component regulatory system with citb	0.0633	0.0686
JW0612	*citB*	Response regulator in two-component regulatory system with cita	0.0376	0.2979
JW0613	*dcuC*	CP4-44 prophage; putative disrupted hemin or colicin receptor	-0.0070	0.7961
JW0616	*dcuC*	CP4-44 prophage; putative disrupted hemin or colicin receptor	-0.0117	0.6716
JW0617	*crcA*	Phospholipid:lipid A palmitoyltransferase	0.0115	0.8187
JW0618	*cspE*	Constitutive cold shock family transcription antitermination protein; negative regulator of	-0.0525	0.1275

		cspa transcription; RNA melting protein; ssdna-binding protein		
JW0619	*crcB*	Fluoride efflux channel, dual topology membrane protein	-0.0199	0.7570
JW0620	*ybeH*	CP4-44 prophage; putative disrupted hemin or colicin receptor	0.0041	0.8888
JW0621	*ybeM*	CP4-44 prophage; putative disrupted hemin or colicin receptor	0.0447	0.0491
JW0623	*lipA*	Lipoyl synthase	-0.3178	0.0025
JW0624	*ybeF*	Lysr family putative transcriptional regulator	0.0599	0.0184
JW0626	*ybeD*	UPF0250 family protein	-0.0671	0.2183
JW0628	*rlpA*	Septal ring protein, suppressor of prc, minor lipoprotein	0.0846	0.0002
JW0631	*ybeA*	23S rrna m(3)Psi1915 pseudouridine methyltransferase, SAM-dependent	-0.0555	0.0333
JW0633	*cobC*	Putative alpha-ribazole-5'-P phosphatase	0.1701	0.0253
JW0638	*ybeL*	DUF1451 family protein	0.2345	0.0633
JW0640	*ybeR*	DUF1266 family protein	-0.1602	0.2288
JW0642	*ybeT*	Sel1 family TPR-like repeat protein	0.0284	0.3677
JW0644	*djlC*	J domain-containing hscc co-chaperone; Hsc56	0.0817	0.1717
JW0645	*hscC*	Hsp70 family chaperone Hsc62; rpod-binding transcription inhibitor	0.0350	0.1883
JW0646	*rihA*	Ribonucleoside hydrolase 1	0.0151	0.7904
JW0647	*gltL*	Glutamate/aspartate ABC transporter atpase	-0.0924	0.0270
JW0648	*gltK*	Glutamate/aspartate ABC transporter permease	0.0759	0.1957
JW0649	*gltJ*	Glutamate/aspartate ABC transporter permease	0.0112	0.6033
JW0655	*ybeX*	Putative ion transport	-0.0209	0.5041
JW0656	*ybeY*	Ssrna-specific endoribonuclease; 16S rrna 3' end maturation and quality control co-endoribonuclease working with rnase R; rrna transcription antitermination factor	-0.0657	0.1086
JW0657	*ybeZ*	Heat shock protein, putative ntpase; phoh-like protein	0.0446	0.2721
JW0658	*miaB*	Trna-i(6)A37 methylthiotransferase	0.0818	0.2146
JW0659	*ubiF*	2-octaprenyl-3-methyl-6-methoxy-1,4-benzoquinol oxygenase	-0.0682	0.0589
JW0660	*asnB*	Asparagine synthetase B	0.0326	0.3188
JW0661	*nagD*	UMP phosphatase	-0.0485	0.1483
JW0662	*nagC*	N-acetylglucosamine-inducible nag divergent operon transcriptional repressor	-0.1908	0.0019
JW0663	*nagA*	N-acetylglucosamine-6-phosphate deacetylase	0.1164	0.0631
JW0664	*nagB*	Glucosamine-6-phosphate deaminase	0.0780	0.0207
JW0667	*ybfM*	Chitoporin, uptake of chitosugars	0.1052	0.0813
JW0668	*ybfN*	Chitosugar-induced verified lipoprotein	0.0040	0.8985
JW0669	*fur*	Ferric iron uptake regulon transcriptional repressor; autorepressor	0.0318	0.5215
JW0674	*seqA*	Negative modulator of initiation of replication	-0.0103	0.8430
JW0675	*pgm*	Phosphoglucomutase	0.1333	0.1494

JW0676	*ybfP*	Lipoprotein	-0.0249	0.2222
JW0679	*potE*	Putrescine/proton symporter: putrescine/ornithine antiporter	-0.0089	0.6763
JW0680	*speF*	Ornithine decarboxylase isozyme, inducible	0.0112	0.8211
JW0683	*kdpD*	Fused sensory histidine kinase in two-component regulatory system with kdpe: signal sensing protein	-0.0422	0.3336
JW0684	*kdpC*	Potassium translocating atpase, subunit C	-0.0511	0.0371
JW0685	*kdpB*	Potassium translocating atpase, subunit B	0.0028	0.8971
JW0686	*kdpA*	Potassium translocating atpase, subunit A	-0.0035	0.9545
JW0687	*kdpF*	Potassium ion accessory transporter subunit	-0.0729	0.2746
JW0688	*ybfA*	DUF2517 family protein	-0.0104	0.5576
JW0689	*rhsC*	Rhs protein with putative toxin domain; putative neighboring cell growth inhibitor	-0.0051	0.8174
JW0691	*ybfB*	Putative membrane protein	0.0250	0.8436
JW0692	*ybfO*	Pseudogene, Rhs family	0.0148	0.6654
JW0693	*ybfC*	Putative secreted protein	0.0329	0.7769
JW0695	*ybfL*	Pseudogene, DDE domain transposase family	-0.0033	0.8756
JW0696	*ybfD*	H repeat-associated putative transposase	-0.0296	0.5886
JW0697	*ybgA*	DUF1722 family protein	-0.0525	0.2462
JW0698	*phr*	Deoxyribodipyrimidine photolyase, FAD-binding	0.1389	0.0300
JW0699	*ybgH*	Dipeptide and tripeptide permease D	-0.0580	0.0701
JW0700	*ybgI*	GTP cyclohydrolase-like radiation resistance protein; metal-binding	0.0618	0.0062
JW0701	*ybgJ*	Putative allophanate hydrolase, subunit 1	0.0031	0.9149
JW0702	*ybgK*	Putative allophanate hydrolase, subunit 2	-0.0326	0.4000
JW0703	*ybgL*	UPF0271 family protein	-0.0599	0.2199
JW0704	*nei*	Endonuclease VIII and 5-formyluracil/5-hydroxymethyluracil DNA glycosylase	0.0594	0.1862
JW0707	*ybgP*	Putative periplasmic pilin chaperone	-0.0090	0.8590
JW0709	*ybgD*	Putative fimbrial-like adhesin protein	0.0566	0.0579
JW0710	*gltA*	Citrate synthase	0.0427	0.3401
JW0711	*sdhC*	Succinate dehydrogenase, membrane subunit, binds cytochrome b556	0.2135	0.0188
JW0712	*sdhD*	Succinate dehydrogenase, membrane subunit, binds cytochrome b556	-0.0172	0.7319
JW0714	*sdhB*	Succinate dehydrogenase, fes subunit	0.0765	0.2140
JW0715	*sucA*	2-oxoglutarate decarboxylase, thiamine triphosphate-binding	0.0219	0.2638
JW0716	*sucB*	Dihydrolipoyl succinyltransferase, subunit of 2-oxoglutarate dehydrogenase	-0.0238	0.4334
JW0717	*sucC*	Succinyl-coa synthetase, beta subunit	-0.0753	0.1184
JW0718	*sucD*	Succinyl-coa synthetase, NAD(P)-binding, alpha subunit	0.0546	0.0519
JW0719	*mngR*	Transcriptional repressor for the mannosyl-D-glycerate catabolic operon	0.0091	0.6498

JW0720	*mngA*	Fused 2-O-a-mannosyl-D-glycerate specific PTS enzymes: IIA component/IIB component/IIC component	-0.0396	0.4701
JW0721	*mngB*	Alpha-mannosidase	-0.0014	0.9606
JW0723	*cydB*	Cytochrome d terminal oxidase, subunit II	0.0692	0.2180
JW0724	*ybgT*	Cytochrome d (bd-I) ubiquinol oxidase subunit X	-0.0120	0.6921
JW0725	*ybgE*	Putative inner membrane protein in cydabx-ybge operon	-0.0680	0.0691
JW0726	*ybgC*	Acyl-coa thioester hydrolase	-0.0001	0.9978
JW0727	*tolQ*	Membrane spanning protein in tola-tolq-tolr complex	-0.0427	0.3058
JW0728	*tolR*	Membrane spanning protein in tola-tolq-tolr complex	0.0770	0.0370
JW0729	*tolA*	Membrane anchored protein in tola-tolq-tolr complex	-0.0187	0.5552
JW0731	*pal*	Peptidoglycan-associated outer membrane lipoprotein	0.0762	0.0685
JW0732	*ybgF*	Periplasmic tola-binding protein	-0.0245	0.4913
JW0733	*nadA*	Quinolinate synthase, subunit A	0.1018	0.0158
JW0734	*pnuC*	Nicotinamide riboside transporter	0.0649	0.2637
JW0735	*zitB*	Zinc efflux system	0.0507	0.3648
JW0736	*ybgS*	Putative periplasmic protein	-0.0125	0.7878
JW0737	*aroG*	3-deoxy-D-arabino-heptulosonate-7-phosphate synthase, phenylalanine repressible	-0.0579	0.2842
JW0738	*gpmA*	Phosphoglyceromutase 1	-0.1748	0.0001
JW0740	*galK*	Galactokinase	-0.1040	0.0040
JW0741	*galT*	Galactose-1-phosphate uridylyltransferase	-0.0491	0.0444
JW0742	*galE*	UDP-galactose-4-epimerase	-0.0157	0.5908
JW0743	*modF*	Molybdate ABC transporter atpase	-0.0238	0.6270
JW0744	*modE*	Transcriptional repressor for the molybdenum transport operon modabc	-0.0565	0.0153
JW0746	*modA*	Molybdate-binding protein moda	0.0623	0.1778
JW0747	*modB*	Molybdate ABC transporter permease; chlorate resistance protein	0.1142	0.0118
JW0748	*modC*	Molybdate ABC transporter atpase; chlorate resistance protein	0.0864	0.1413
JW0749	*ybhA*	Pyridoxal phosphate (PLP) phosphatase	0.0130	0.6270
JW0750	*ybhE*	6-phosphogluconolactonase	0.0290	0.3805
JW0752	*ybhH*	Putative prpf family isomerase	0.0230	0.3954
JW0753	*ybhI*	Putative DASS family tricarboxylate or dicarboxylate transporter	-0.0267	0.5251
JW0756	*ybhB*	Kinase inhibitor homolog, UPF0098 family	0.0125	0.3522
JW0757	*bioA*	7,8-diaminopelargonic acid synthase, PLP-dependent	0.1081	0.0090
JW0758	*bioB*	Biotin synthase	0.1570	0.0006
JW0759	*bioF*	8-amino-7-oxononanoate synthase	0.1833	0.0004
JW0760	*bioC*	Malonyl-ACP O-methyltransferase, SAM-dependent	0.0216	0.5398

JW0761	*bioD*	Dethiobiotin synthetase	-0.0268	0.6669
JW0762	*uvrB*	Exision nuclease of nucleotide excision repair, DNA damage recognition component	0.0747	0.1059
JW0763	*ybhK*	Putative cofd superfamily transferase	-0.0518	0.4211
JW0764	*moaA*	Molybdopterin biosynthesis protein A	0.0179	0.6359
JW0766	*moaC*	Molybdopterin biosynthesis, protein C	0.0193	0.5725
JW0767	*moaD*	Molybdopterin synthase, small subunit	0.0997	0.0114
JW0768	*moaE*	Molybdopterin synthase, large subunit	0.0625	0.0013
JW0769	*ybhL*	Putative acetate transporter; BAX Inhibitor-1 family inner membrane protein	-0.0066	0.8836
JW0770	*ybhM*	BAX Inhibitor-1 family inner membrane protein	-0.1157	0.0282
JW0771	*ybhN*	Inner membrane protein ybhn	-0.0022	0.9444
JW0772	*ybhO*	Cardiolipin synthase 2	0.0059	0.8340
JW0774	*ybhQ*	Inner membrane protein	0.0569	0.0116
JW0777	*ybhS*	Putative ABC transporter permease	0.0433	0.1267
JW0779	*ybhG*	Putative membrane fusion protein (MFP) component of efflux pump, membrane anchor	-0.0261	0.4784
JW0780	*ybiH*	DUF1956 domain-containing tetr family putative transcriptional regulator	0.0196	0.5485
JW0781	*rhlE*	ATP-dependent RNA helicase	-0.0631	0.1120
JW0783	*ybiA*	DUF1768 family protein	0.0505	0.7370
JW0784	*dinG*	ATP-dependent DNA helicase	-0.0633	0.1858
JW0785	*ybiB*	Putative family 3 glycosyltransferase	0.0867	0.0228
JW0786	*ybiC*	Hydroxycarboxylate dehydrogenase B	-0.0213	0.6405
JW0788	*ybiI*	Dksa-type zinc finger protein	-0.3346	0.0482
JW0790	*fiu*	Catecholate siderophore receptor	-0.0526	0.0126
JW0794	*glnQ*	Glutamine transporter subunit	-0.0981	0.0001
JW0795	*glnP*	Glutamine transporter subunit	-0.0020	0.9486
JW0796	*glnH*	Glutamine transporter subunit	0.0140	0.6115
JW0797	*dps*	Fe-binding and storage protein; stress-inducible DNA-binding protein	-0.0237	0.4468
JW0798	*rhtA*	Threonine and homoserine efflux system	0.0074	0.8357
JW0799	*ompX*	Outer membrane protein X	0.0040	0.8955
JW0800	*ybiP*	OPG biosynthetic transmembrane phosphoethanolamine transferase	0.0240	0.3316
JW0801	*mntR*	Mn(2+)-responsive manganese regulon transcriptional regulator	0.0152	0.7726
JW0802	*ybiR*	Putative arsb family transporter; inner membrane protein	-0.0201	0.6142
JW0803	*ybiS*	L,D-transpeptidase linking Lpp to murein	-0.0208	0.5051
JW0804	*ybiT*	ABC-F family putative regulatory atpase	0.0197	0.6556
JW0805	*ybiU*	DUF1479 family protein	0.0660	0.5892
JW0806	*ybiV*	Sugar phosphatase; fructose-1-P/ribose-5-P/glucose-6-P phosphatase	0.0759	0.0068
JW0807	*ybiW*	Putative pyruvate formate lyase	-0.0767	0.0187
JW0808	*ybiY*	Putative pyruvate formate lyase activating enzyme	0.0216	0.7201

JW0810	*moeB*	Molybdopterin synthase sulfurylase	0.0454	0.1341
JW0811	*moeA*	Molybdopterin molybdenumtransferase; molybdopterin biosynthesis protein	0.0366	0.3277
JW0812	*iaaA*	Isoaspartyl peptidase	-0.0280	0.3000
JW0815	*yliC*	Glutathione ABC transporter permease	-0.0836	0.0140
JW0816	*yliD*	Glutathione ABC transporter permease	-0.0139	0.6931
JW0817	*yliE*	Putative membrane-anchored cyclic-di-GMP phosphodiesterase	0.0217	0.5794
JW0818	*yliF*	Putative membrane-anchored diguanylate cyclase	0.0075	0.8537
JW0819	*yliG*	Ribosomal protein S12 methylthiotransferase	-0.1013	0.0309
JW0820	*yliH*	Repressor of biofilm formation by indole transport regulation	0.0482	0.1943
JW0821	*yliI*	Soluble aldose sugar dehydrogenase	0.0313	0.2685
JW0822	*yliJ*	Glutathione S-transferase	0.0495	0.6729
JW0824	*deoR*	Deoxyribose-5-phosphate-inducible deoxyribose operon transcriptional repressor; repressor of nupg and tsx	-0.1311	0.0281
JW0826	*cmr*	Multidrug efflux system protein	0.0795	0.0002
JW0827	*ybjH*	Uncharacterized protein	-0.0261	0.6375
JW0829	*ybjJ*	Putative drug efflux MFS transporter, inner membrane protein	0.0954	0.0632
JW0831	*ybjL*	Putative transporter	0.0133	0.3645
JW0832	*ybjM*	Inner membrane protein	-0.0911	0.0508
JW0833	*grxA*	Glutaredoxin 1, redox coenzyme for ribonucleotide reductase (RNR1a)	-0.0167	0.7490
JW0834	*ybjC*	DUF1418 family protein	-0.0335	0.3211
JW0835	*nfsA*	Nitroreductase A, NADPH-dependent, FMN-dependent	-0.0210	0.3119
JW0836	*rimK*	Ribosomal protein S6 modification protein	-0.0170	0.6719
JW0838	*potF*	Putrescine ABC transporter periplasmic binding protein	0.1032	0.1739
JW0840	*potH*	Putrescine ABC transporter permease	0.0044	0.9087
JW0841	*potI*	Putrescine ABC transporter permease	0.0566	0.1569
JW0842	*ybjO*	DUF2593 family inner membrane protein	0.0965	0.4684
JW0843	*rumB*	23S rrna (uracil(747)-C(5))-methyltransferase rlmc	0.0248	0.5281
JW0844	*artJ*	Arginine ABC transporter periplasmic binding protein	-0.0248	0.4953
JW0845	*artM*	Arginine ABC transporter permease	0.0232	0.4346
JW0846	*artQ*	Arginine ABC transporter permease	-0.0419	0.1942
JW0847	*artI*	Arginine transporter subunit	-0.0380	0.5351
JW0848	*artP*	Arginine ABC transporter atpase	-0.0471	0.1816
JW0849	*ybjP*	Lipoprotein	0.0066	0.8336
JW0850	*ybjQ*	UPF0145 family protein	-0.0266	0.8436
JW0851	*ybjR*	1,6-anhydro-N-acetylmuramyl-L-alanine amidase, Zn-dependent; OM lipoprotein	0.0375	0.1005
JW0854	*ltaE*	L-allo-threonine aldolase, PLP-dependent	0.0341	0.3333

JW0855	*poxB*	Pyruvate dehydrogenase, thiamine triphosphate-binding, FAD-binding	0.0474	0.2730
JW0857	*hcp*	Hydroxylamine reductase	0.0000	0.9985
JW0858	*ybjE*	Putative transporter	-0.0032	0.9209
JW0859	*aqpZ*	Aquaporin Z	0.1158	0.0013
JW0860	*ybjD*	Putative OLD family ATP-dependent endonuclease; DUF2813 family protein	0.1441	0.2879
JW0861	*ybjX*	DUF535 family protein	0.0166	0.3596
JW0862	*macA*	Macrolide transporter membrane fusion protein (MFP) component	-0.0228	0.4021
JW0863	*macB*	Macrolide ABC transporter peremase/atpase	-0.0537	0.2148
JW0864	*cspD*	Inhibitor of DNA replication, cold shock protein homolog	0.0385	0.3211
JW0865	*clpS*	Regulatory protein for clpa substrate specificity	0.0093	0.7536
JW0866	*clpA*	Atpase and specificity subunit of clpa-clpp ATP-dependent serine protease, chaperone activity	-0.0248	0.5470
JW0868	*aat*	Leucyl/phenylalanyl-trna-protein transferase	0.0498	0.3120
JW0870	*cydD*	Glutathione/cysteine ABC transporter export permease/atpase	0.0254	0.6813
JW0871	*trxB*	Thioredoxin reductase, FAD/NAD(P)-binding	0.0675	0.2056
JW0872	*lrp*	Leucine-responsive global transcriptional regulator	-0.0627	0.2691
JW0875	*ycaJ*	Recombination intermediate processing DNA-dependent atpase	-0.0260	0.4917
JW0878	*dmsB*	Dimethyl sulfoxide reductase, anaerobic, subunit B	-0.0179	0.5967
JW0879	*dmsC*	Dimethyl sulfoxide reductase, anaerobic, subunit C	0.0091	0.7445
JW0880	*ycaC*	Putative isochorismatase family hydrolase	-0.0210	0.5845
JW0881	*ycaD*	Putative MFS transporter, inner membrane protein	0.0609	0.3984
JW0884	*ycaK*	Putative NAD(P)H-dependent oxidoreductase	-0.0375	0.2679
JW0885	*pflA*	Pyruvate formate-lyase 1-activating enzyme; [formate-C-acetyltransferase 1]-activating enzyme; PFL activase	-0.0288	0.2610
JW0886	*pflB*	Formate C-acetyltransferase 1, anaerobic; pyruvate formate-lyase 1	-0.0817	0.0289
JW0887	*focA*	Formate channel	-0.0029	0.9310
JW0888	*ycaO*	Ribosomal protein S12 methylthiotransferase accessory factor	0.0542	0.2766
JW0889	*ycaP*	UPF0702 family putative inner membrane protein	-0.0411	0.3690
JW0890	*serC*	3-phosphoserine/phosphohydroxythreonine aminotransferase	-0.0944	0.1109
JW0891	*aroA*	5-enolpyruvylshikimate-3-phosphate synthetase	0.0553	0.0730
JW0892	*ycaL*	Putative peptidase-related chaperone	0.1320	0.0004
JW0893	*cmk*	Cytidylate kinase	-0.0668	0.2819
JW0895	*ihfB*	Integration host factor (IHF), DNA-binding protein, beta subunit	0.1517	0.1056

JW0899	*ycaQ*	DUF1006 family protein with C-terminal whth domain	-0.0213	0.4513
JW0900	*ycaR*	Peroxide and acid resistance protein, UPF0434 family	0.0279	0.6251
JW0902	*ycbJ*	Uncharacterized protein	0.0575	0.7053
JW0904	*smtA*	Putative S-adenosyl-L-methionine-dependent methyltransferase	0.0349	0.1272
JW0908	*ycbB*	Murein L,D-transpeptidase	-0.0775	0.0322
JW0909	*ycbK*	M15A protease-related family periplasmic protein	-0.0134	0.6681
JW0910	*ycbL*	Putative metal-binding enzyme	0.0409	0.4393
JW0912	*ompF*	Outer membrane porin 1a (Ia;b;F)	-0.0076	0.8917
JW0914	*pncB*	Nicotinate phosphoribosyltransferase	-0.0718	0.0633
JW0915	*pepN*	Aminopeptidase N	0.0562	0.2382
JW0916	*ssuB*	Aliphatic sulfonate ABC transporter atpase	0.0392	0.1821
JW0918	*ssuD*	Alkanesulfonate monooxygenase, FMNH(2)-dependent	-0.0371	0.5026
JW0919	*ssuA*	Aliphatic sulfonate ABC transporter periplasmic binding protein	-0.0591	0.3424
JW0920	*ssuE*	NAD(P)H-dependent FMN reductase	0.0032	0.9372
JW0922	*ycbR*	Putative periplasmic pilin chaperone	0.0224	0.5539
JW0923	*ycbS*	Putative outer membrane fimbrial subunit export usher protein	0.0505	0.2884
JW0924	*ycbT*	Putative fimbrial-like adhesin protein	-0.2011	0.1055
JW0925	*ycbU*	Putative fimbriae protein	0.0388	0.7305
JW0928	*pyrD*	Dihydro-orotate oxidase, FMN-linked	0.0019	0.9633
JW0931	*ycbY*	23S rrna m(2)G2445 and m(7)G2069 methyltransferases, SAM-dependent	0.0471	0.2322
JW0932	*uup*	Replication regulatory ABC-F family DNA-binding atpase	0.0580	0.2104
JW0933	*pqiA*	Inner membrane subunit of the putative pqiabc transporter	0.0173	0.6157
JW0934	*pqiB*	Periplasmic MCE subunit of the putative pqiabc transporter, IM-anchored, paraquat-inducible	0.0036	0.9077
JW0936	*rmf*	Ribosome modulation factor	-0.0358	0.3865
JW0938	*ycbZ*	Putative peptidase	-0.0231	0.3210
JW0939	*ycbG*	Ter macrodomain organizer mats-binding protein	-0.0269	0.1743
JW0940	*ompA*	Outer membrane protein A (3a;II*;G;d)	-0.0042	0.9403
JW0944	*yccF*	DUF307 family inner membrane protein	0.0243	0.4447
JW0945	*helD*	DNA helicase IV	0.0147	0.7413
JW0947	*yccT*	UPF0319 family protein	0.0165	0.6353
JW0952	*yccK*	Mnm(5)-s(2)U34-trna 2-thiolation sulfurtransferase	-0.0220	0.5169
JW0953	*yccA*	Modulator of ftsh protease, inner membrane protein	0.0520	0.1309
JW0954	*hyaA*	Hydrogenase 1, small subunit	-0.0267	0.4669
JW0955	*hyaB*	Hydrogenase 1, large subunit	-0.0180	0.5548
JW0956	*hyaC*	Hydrogenase 1, b-type cytochrome subunit	0.1174	0.0466

JW0957	*hyaD*	Hydrogenase 1 maturation protease	0.0189	0.6880
JW0958	*hyaE*	Putative hyaa chaperone	0.0561	0.2015
JW0960	*appC*	Cytochrome bd-II oxidase, subunit I	0.0467	0.1665
JW0961	*appB*	Cytochrome bd-II oxidase, subunit II	-0.0204	0.5795
JW0963	*appA*	Phosphoanhydride phosphorylase	0.0246	0.4752
JW0964	*yccC*	Tyrosine-protein kinase, role in O-antigen capsule formation	0.0085	0.7371
JW0966	*yccZ*	Putative O-antigen capsule outer membrane auxillary protein export channel	0.0327	0.4699
JW0967	*ymcA*	Putative O-antigen capsule production periplasmic protein	-0.0219	0.5690
JW0968	*ymcB*	Putative O-antigen capsule production periplasmic protein	-0.0536	0.1227
JW0969	*ymcC*	O-antigen capsule production lipoprotein	-0.1005	0.0136
JW0974	*cspG*	Cold shock protein homolog, cold-inducible	-0.0301	0.5372
JW0975	*ymcE*	Cold shock gene	-0.0096	0.8698
JW0976	*gnsA*	Putative phosphatidylethanolamine synthesis regulator	0.0219	0.2765
JW0977	*yccM*	Putative 4Fe-4S membrane protein	0.0595	0.0191
JW0979	*torT*	Periplasmic protein tort	-0.0058	0.8843
JW0980	*torR*	Response regulator in two-component regulatory system with tors	-0.0543	0.1217
JW0981	*torC*	Trimethylamine N-oxide (TMAO) reductase I, cytochrome c-type subunit	0.2593	0.0025
JW0982	*torA*	Trimethylamine N-oxide (TMAO) reductase I, catalytic subunit	0.0030	0.9118
JW0983	*torD*	Tora-maturation chaperone	0.0273	0.4712
JW0984	*cbpM*	Chaperone modulatory protein cbpm	0.0468	0.1991
JW0986	*yccE*	PRK09784 family protein	0.1382	0.0005
JW0987	*agp*	Glucose-1-phosphatase/inositol phosphatase	-0.0240	0.6534
JW0988	*yccJ*	Uncharacterized protein	0.0449	0.1605
JW0994	*rarA*	Putative reactive intermediate detoxifying aminoacrylate hydrolase	0.0161	0.6213
JW0995	*ycdK*	Putative aminoacrylate deaminase, reactive intermediate detoxification; weak enamine/imine deaminase activity	0.0363	0.2183
JW0997	*ycdM*	Pyrimidine oxygenase, FMN-dependent	-0.0034	0.9188
JW0998	*ycdC*	Rut operon transcriptional repressor for	-0.0607	0.0898
JW0999	*putA*	Fused DNA-binding transcriptional regulator/proline dehydrogenase/pyrroline-5-carboxylate dehydrogenase	0.0056	0.9214
JW1001	*putP*	Proline:sodium symporter	-0.0230	0.5567
JW1002	*ycdN*	CP4-44 prophage; putative disrupted hemin or colicin receptor	0.0633	0.0696
JW1003	*ycdO*	Inactive ferrous ion transporter efeuob	0.0025	0.8930
JW1005	*phoH*	ATP-binding protein; putative phoh family P-loop atpase	-0.0600	0.0390

JW1006	*ycdP*	Biofilm PGA synthase pgacd, regulatory subunit; c-di-GMP-stimulated activity and dimerization	0.0116	0.5323
JW1007	*ycdQ*	Biofilm PGA synthase pgacd, catalytic subunit; poly-beta-1,6-N-acetyl-D-glucosamine synthase	-0.1451	0.0023
JW1010	*ycdS*	Biofilm adhesin polysaccharide PGA secretin; OM porin; poly-beta-1,6-N-acetyl-D-glucosamine export protein	0.1072	0.0066
JW1015	*ycdU*	Putative inner membrane protein	-0.0420	0.1079
JW1017	*ycdX*	Alkaline phosphatase	0.0116	0.7615
JW1018	*ycdY*	Redox enzyme maturation protein (REMP) chaperone for ycdx	0.0620	0.6888
JW1020	*csgG*	Curli production assembly/transport outer membrane lipoprotein	0.1193	0.0619
JW1021	*csgF*	Curli nucleation outer membrane protein	0.0560	0.0006
JW1022	*csgE*	Curlin secretion specificity factor	0.0070	0.8888
JW1023	*csgD*	Csgbac operon transcriptional regulator	-0.0450	0.5252
JW1024	*csgB*	Curlin nucleator protein, minor subunit in curli complex	0.0379	0.3545
JW1026	*csgC*	Curli assembly protein	-0.0832	0.0919
JW1031	*ymdA*	Uncharacterized protein	-0.0078	0.8087
JW1032	*ymdB*	O-acetyl-ADP-ribose deacetylase; rnase III inhibitor during cold shock; putative cardiolipin synthase C regulatory subunit	0.1955	0.1643
JW1034	*mdoC*	OPG biosynthetic transmembrane succinyltransferase	0.0812	0.0023
JW1035	*mdoG*	OPG biosynthetic periplasmic beta-1,6 branching glycosyltransferase	0.0764	0.0429
JW1037	*mdoH*	OPG biosynthetic ACP-dependent transmembrane UDP-glucose beta-1,2 glycosyltransferase; nutrient-dependent cell size regulator, ftsz assembly antagonist	0.0624	0.0237
JW1039	*msyB*	Multicopy suppressor of secy and seca	-0.0624	0.0872
JW1040	*mdtG*	Putative drug efflux system protein	0.0420	0.0647
JW1041	*lpxL*	Lauryl-acyl carrier protein (ACP)-dependent acyltransferase	-0.0310	0.5969
JW1042	*yceA*	Putative rhodanese-related sulfurtransferase	-0.0349	0.3689
JW1043	*yceI*	Periplasmic high ph-inducible lipid-binding protein	0.0239	0.7737
JW1044	*yceJ*	Putative cytochrome b561	0.1338	0.3341
JW1045	*yceO*	Uncharacterized protein	0.1576	0.0007
JW1048	*dinI*	DNA damage-inducible protein I	0.1392	0.3094
JW1049	*pyrC*	Dihydro-orotase	0.0446	0.0428
JW1050	*yceB*	Lipoprotein, DUF1439 family	0.0333	0.2806
JW1051	*grxB*	Glutaredoxin 2 (Grx2)	-0.0275	0.4561
JW1052	*mdtH*	Multidrug resistance efflux transporter conferring overexpression resistance to norfloxacin and enoxacin	0.0954	0.0139
JW1053	*rimJ*	Ribosomal-protein-S5-alanine N-acetyltransferase	-0.1045	0.0014
JW1054	*yceH*	UPF0502 family protein	-0.1624	0.0075

JW1055	*mviM*	Putative oxidoreductase	-0.0498	0.2640
JW1057	*flgN*	Export chaperone for flgk and flgl	0.0589	0.0933
JW1059	*flgA*	Assembly protein for flagellar basal-body periplasmic P ring	-0.0108	0.7172
JW1060	*flgB*	Flagellar component of cell-proximal portion of basal-body rod	-0.0833	0.0955
JW1061	*flgC*	Flagellar component of cell-proximal portion of basal-body rod	0.0424	0.4548
JW1062	*flgD*	Flagellar hook assembly protein	0.0430	0.1903
JW1063	*flgE*	Flagellar hook protein	-0.0280	0.4663
JW1064	*flgF*	Flagellar component of cell-proximal portion of basal-body rod	-0.0599	0.3005
JW1067	*flgI*	Putative flagellar basal body protein	0.0160	0.7080
JW1068	*flgJ*	Bifunctional flagellar rod assembly protein (N-terminal) and beta-N-acetylglucosaminidase (C-terminal)	-0.0400	0.1543
JW1069	*flgK*	Flagellar hook-filament junction protein 1	0.0440	0.2118
JW1070	*flgL*	Flagellar hook-filament junction protein	0.0343	0.3256
JW1072	*rluC*	23S rrna pseudouridine(955,2504,2580) synthase	0.0297	0.5075
JW1075	*rpmF*	50S ribosomal subunit protein L32	-0.0817	0.0552
JW1077	*fabH*	3-oxoacyl-[acyl-carrier-protein] synthase III	0.0433	0.3873
JW1081	*fabF*	3-oxoacyl-[acyl-carrier-protein] synthase II	-0.0596	0.1208
JW1082	*pabC*	4-amino-4-deoxychorismate lyase component of para-aminobenzoate synthase multienzyme complex	-0.2580	0.0003
JW1083	*yceG*	Septation protein, ampicillin sensitivity	0.0243	0.3849
JW1086	*ycfH*	Putative dnase	0.0255	0.4382
JW1087	*ptsG*	Fused glucose-specific PTS enzymes: IIB component/IIC component	0.0348	0.5871
JW1089	*hinT*	Purine nucleoside phosphoramidase	0.0267	0.3218
JW1090	*ycfL*	Uncharacterized protein	0.0111	0.6085
JW1092	*ycfN*	Thiamine kinase	-0.0290	0.4450
JW1093	*nagZ*	Beta N-acetyl-glucosaminidase	-0.0376	0.4920
JW1095	*ndh*	Respiratory NADH dehydrogenase 2/cupric reductase	-0.0117	0.6204
JW1096	*ycfJ*	Uncharacterized protein	-0.0056	0.8771
JW1098	*ycfR*	Biofilm, cell surface and signaling protein	0.0633	0.0253
JW1100	*mfd*	Transcription-repair coupling factor	0.1260	0.3375
JW1101	*ycfT*	Inner membrane protein	-0.0694	0.0723
JW1105	*ycfX*	N-acetyl-D-glucosamine kinase	0.0724	0.0054
JW1106	*cobB*	Deacetylase of acs and chey, chemotaxis regulator	-0.0678	0.1934
JW1107	*ycfZ*	Inner membrane protein ycfz	0.0249	0.3718
JW1109	*potD*	Spermidine/putrescine ABC transporter periplasmic binding protein	0.0192	0.6828
JW1110	*potC*	Spermidine/putrescine ABC transporter permease	0.0339	0.4288
JW1111	*potB*	Spermidine/putrescine ABC transporter permease	0.0387	0.3627
JW1112	*potA*	Spermidine/putrescine ABC transporter atpase	-0.0471	0.0025

JW1113	*pepT*	Peptidase T	0.0335	0.2802
JW1114	*ycfD*	50S ribosomal protein L16 arginine hydroxylase; 2-oxoglutarate oxygenase	0.0651	0.2425
JW1115	*phoQ*	Sensory histidine kinase in two-component regulatory system with phop	0.0836	0.0769
JW1116	*phoP*	Response regulator in two-component regulatory system with phoq	0.0206	0.3042
JW1119	*trmU*	Trna(Gln,Lys,Glu) U34 2-thiouridylase	-0.2118	0.0000
JW1120	*ymfB*	Bifunctional thiamine pyrimidine pyrophosphate hydrolase and thiamine pyrophosphate hydrolase	-0.0428	0.1319
JW1121	*ymfC*	23S rrna pseudouridine(2457) synthase	-0.0712	0.0933
JW1122	*icd*	Isocitrate dehydrogenase; e14 prophage attachment site; tellurite reductase	0.1253	0.0121
JW1123	*ymfD*	E14 prophage; putative SAM-dependent methyltransferase	-0.0113	0.7758
JW1125	*lit*	T4 phage exclusion protein; cell death peptidase, e14 prophage	-0.0301	0.5622
JW1126	*intE*	E14 prophage; putative integrase	-0.0981	0.0284
JW1127	*ymfG*	E14 prophage; putative excisionase	0.0486	0.0292
JW1128	*ymfH*	Uncharacterized protein	0.0640	0.0259
JW1130	*ymfJ*	Uncharacterized protein	-0.2599	0.0000
JW1133	*ymfL*	E14 prophage; putative DNA-binding transcriptional regulator	0.0274	0.2161
JW1134	*ymfM*	E14 prophage; uncharacterized protein	-0.0818	0.1192
JW1135	*ymfN*	Pseudogene, phage terminase protein A family, e14 prophage	0.0849	0.1899
JW1136	*ymfR*	E14 prophage; uncharacterized protein	-0.0050	0.7627
JW1137	*ymfO*	Pseudogene, portal protein family, e14 prophage	-0.0079	0.7487
JW1139	*ymfQ*	Prohage e14 tail protein homolog	0.0199	0.0784
JW1142	*tfaE*	E14 prophage; putative tail fiber assembly protein	0.0745	0.0093
JW1144	*pin*	E14 prophage; site-specific DNA recombinase	0.1127	0.0009
JW1145	*mcrA*	Putative 5-methylcytosine/5-hydroxymethylcytosine-specific restriction nuclease; 5-methylcytosine DNA binding protein	0.0120	0.4671
JW1147	*elbA*	Rpos stabilzer during Mg starvation, anti-rssb factor	-0.0699	0.0470
JW1148	*ycgX*	DUF1398 family protein	-0.1008	0.0625
JW1149	*ycgE*	Repressor of blue light-responsive genes	-0.0358	0.1806
JW1150	*ycgF*	Anti-repressor for ycge, blue light-responsive; FAD-binding; inactive c-di-GMP phosphodiesterase-like EAL domain protein	-0.0140	0.7299
JW1151	*ycgZ*	Rcsb connector protein for regulation of biofilm and acid-resistance	-0.0458	0.3618
JW1152	*ymgA*	Rcsb connector protein for regulation of biofilm	0.0133	0.7015
JW1153	*ymgB*	Rcsb connector protein for regulation of biofilm and acid-resistance	0.0370	0.4051
JW1154	*ymgC*	Blue light, low temperature and stress induced protein	-0.0713	0.0140
JW1156	*ymgF*	Inner membrane division septum protein	0.0685	0.0545

JW1162	*ycgI*	CP4-44 prophage; putative disrupted hemin or colicin receptor	-0.0010	0.2181
JW1166	*ycgJ*	Uncharacterized protein	0.0210	0.2000
JW1167	*ycgK*	Periplasmic inhibitor of g-type lysozyme	0.0095	0.7645
JW1168	*ycgL*	UPF0745 family protein	0.0237	0.6364
JW1169	*ycgM*	Putative isomerase/hydrolase	0.0640	0.0868
JW1173	*umuC*	Translesion error-prone DNA polymerase V subunit; DNA polymerase activity	-0.0328	0.5277
JW1175	*nhaB*	Sodium:proton antiporter	0.0738	0.0547
JW1176	*fadR*	Fatty acid metabolism regulon transcriptional regulator	-0.0235	0.5775
JW1177	*ycgB*	Spovr family stationary phase protein	-0.0370	0.1700
JW1178	*dadA*	D-amino acid dehydrogenase	-0.0056	0.8853
JW1179	*dadX*	Alanine racemase, catabolic, PLP-binding	-0.0280	0.4012
JW1181	*ldcA*	Murein tetrapeptide carboxypeptidase; LD-carboxypeptidase A	0.1598	0.0000
JW1183	*ycgR*	Flagellar velocity braking protein, c-di-GMP-regulated	-0.0361	0.3438
JW1184	*ymgE*	UPF0410 family putative inner membrane protein	0.0439	0.0311
JW1185	*ycgY*	Uncharacterized protein	0.0715	0.0010
JW1186	*treA*	Periplasmic trehalase	-0.0091	0.8519
JW1194	*ychF*	Catalase inhibitor protein; atpase, K+-dependent, ribosome-associated	-0.0307	0.1819
JW1196	*ychH*	DUF2583 family putative inner membrane protein	-0.0613	0.2009
JW1204	*ychQ*	SIRB family inner membrane protein	-0.0391	0.1718
JW1205	*ychA*	Transglutaminase-like TPR-repeat protein	0.0233	0.6063
JW1207	*chaA*	Calcium/sodium:proton antiporter	0.0458	0.3100
JW1208	*chaB*	Cation transport regulator	0.0238	0.4896
JW1209	*chaC*	Cation transport regulator	0.0652	0.0219
JW1210	*ychN*	Uncharacterized protein	0.0274	0.2544
JW1211	*ychP*	Putative invasin	0.0014	0.9757
JW1212	*narL*	Response regulator in two-component regulatory system with narx	-0.0845	0.1688
JW1213	*narX*	Sensory histidine kinase in two-component regulatory system with narl	-0.0363	0.2948
JW1214	*narK*	Nitrate/nitrite transporter	-0.0285	0.4847
JW1215	*narG*	Nitrate reductase 1, alpha subunit	-0.0763	0.0021
JW1216	*narH*	Nitrate reductase 1, beta (Fe-S) subunit	-0.0108	0.7194
JW1217	*narJ*	Molybdenum-cofactor-assembly chaperone delta subunit of nitrate reductase 1	0.1171	0.0166
JW1218	*narI*	Nitrate reductase 1, gamma (cytochrome b(NR)) subunit	0.0279	0.5850
JW1219	*tpr*	Protamine-like protein	0.0506	0.1003
JW1220	*purU*	Formyltetrahydrofolate hydrolase	0.1159	0.1572
JW1221	*ychJ*	UPF0225 family protein	0.0441	0.2793
JW1223	*rssB*	Pcnb-degradosome interaction factor; response regulator	0.1519	0.0016

JW1224	*galU*	UTP--glucose-1-phosphate uridylyltransferase	0.1258	0.0088
JW1225	*hns*	Global DNA-binding transcriptional dual regulator H-NS	-0.1748	0.0086
JW1226	*tdk*	Thymidine kinase/deoxyuridine kinase	-0.0347	0.3113
JW1228	*adhE*	Fused acetaldehyde-coa dehydrogenase/iron-dependent alcohol dehydrogenase/pyruvate-formate lyase deactivase	0.0242	0.6568
JW1229	*ychE*	UPF0056 family inner membrane protein	0.0226	0.4710
JW1235	*oppA*	Oligopeptide ABC transporter periplasmic binding protein	0.0502	0.2626
JW1237	*oppC*	Oligopeptide ABC transporter permease	-0.0496	0.0243
JW1238	*oppD*	Oligopeptide ABC transporter atpase	-0.0429	0.0307
JW1239	*oppF*	Oligopeptide ABC transporter atpase	0.0725	0.1608
JW1240	*yciU*	UPF0263 family protein	-0.0547	0.0563
JW1241	*cls*	Cardiolipin synthase 1	-0.1707	0.0007
JW1242	*kch*	Voltage-gated potassium channel	0.0148	0.5672
JW1243	*yciI*	Putative DGPF domain-containing enzyme	-0.0081	0.7564
JW1245	*yciA*	Acyl-coa esterase	0.0046	0.9199
JW1246	*yciB*	Ispa family inner membrane protein	-0.0914	0.0239
JW1247	*yciC*	UPF0259 family inner membrane protein	-0.0645	0.0125
JW1248	*ompW*	Outer membrane protein W	0.0388	0.4573
JW1249	*yciE*	Putative rubrerythrin/ferritin-like metal-binding protein	0.0152	0.6064
JW1250	*yciF*	Putative rubrerythrin/ferritin-like metal-binding protein	0.0625	0.0081
JW1251	*yciG*	KGG family protein	-0.0374	0.4283
JW1252	*trpA*	Tryptophan synthase, alpha subunit	0.2241	0.0528
JW1253	*trpB*	Tryptophan synthase, beta subunit	0.6111	0.0020
JW1254	*trpC*	Indole-3-glycerolphosphate synthetase and N-(5-phosphoribosyl)anthranilate isomerase	0.0531	0.2637
JW1255	*trpD*	Fused glutamine amidotransferase (component II) of anthranilate synthase/anthranilate phosphoribosyl transferase	0.2732	0.0068
JW1256	*trpE*	Component I of anthranilate synthase	0.2306	0.0784
JW1257	*trpL*	Trp operon leader peptide	0.0124	0.7462
JW1258	*yciV*	PHP domain protein	0.0201	0.6064
JW1261	*rluB*	23S rrna pseudouridine(2605) synthase	0.0425	0.3474
JW1262	*btuR*	Cob(I)yrinic acid a,c-diamide adenosyltransferase	0.0150	0.7962
JW1263	*yciK*	Putative emrky-tolc system oxoacyl-(acyl carrier protein) reductase	-0.0899	0.0001
JW1264	*sohB*	Inner membrane protein, S49 peptidase family protein	0.0156	0.6067
JW1265	*yciN*	DUF2498 protein ycin	-0.0302	0.4552
JW1267	*cysB*	N-acetylserine-responsive cysteine regulon transcriptional activator; autorepressor	0.0012	0.9719
JW1268	*acnA*	Aconitate hydratase 1; aconitase A	-0.1841	0.0103
JW1270	*pgpB*	Phosphatidylglycerophosphatase B	0.0400	0.4757

JW1271	*yciS*	DUF1049 family inner membrane protein, function unknown	-0.0688	0.0165
JW1272	*yciM*	LPS regulatory protein; putative modulator of lpxc proteolysis	-0.2170	0.0164
JW1273	*pyrF*	Orotidine-5'-phosphate decarboxylase	0.0562	0.0057
JW1274	*yciH*	Initiation factor function partial mimic, SUI1 family	0.2349	0.0621
JW1275	*osmB*	Osmotically and stress inducible lipoprotein	0.0742	0.0902
JW1276	*yciT*	Global regulator of transcription; deor family	0.1154	0.0001
JW1278	*gmr*	Cyclic-di-GMP phosphodiesterase; csgd regulator; modulator of rnase II stability	-0.0210	0.6480
JW1279	*rnb*	Ribonuclease II	0.0196	0.4592
JW1282	*ycjD*	DUF559 family endonuclease-related protein	-0.0557	0.0700
JW1283	*sapF*	Antimicrobial peptide ABC transporter atpase	-0.0056	0.8323
JW1284	*sapD*	Antimicrobial peptide ABC transporter atpase	0.0690	0.1776
JW1285	*sapC*	Antimicrobial peptide transport ABC transporter permease	-0.0187	0.4939
JW1286	*sapB*	Antimicrobial peptide transport ABC transporter permease	-0.0478	0.1034
JW1287	*sapA*	Antimicrobial peptide transport ABC transporter periplasmic binding protein	0.0314	0.2113
JW1288	*ymjA*	DUF2543 family protein	0.0596	0.0209
JW1289	*puuP*	Putrescine importer	-0.0720	0.0049
JW1291	*puuD*	Gamma-glutamyl-gamma-aminobutyrate hydrolase	-0.0966	0.1445
JW1292	*puuR*	Repressor for the divergent puu operons, putrescine inducible	0.0098	0.8523
JW1293	*puuC*	Gamma-glutamyl-gamma-aminobutyraldehyde dehydrogenase; succinate semialdehyde dehydrogenase	-0.0217	0.2609
JW1294	*puuB*	Gamma-glutamylputrescine oxidoreductase	-0.0800	0.0358
JW1295	*puuE*	4-aminobutyrate aminotransferase, PLP-dependent	0.1173	0.0902
JW1296	*pspF*	Psp operon transcriptional activator	0.0332	0.5197
JW1297	*pspA*	Regulatory protein for phage-shock-protein operon	-0.1999	0.1350
JW1298	*pspB*	Psp operon transcription co-activator	-0.1342	0.0002
JW1299	*pspC*	Psp operon transcription co-activator	0.0202	0.5291
JW1300	*pspD*	Peripheral inner membrane phage-shock protein	-0.0056	0.8705
JW1301	*pspE*	Thiosulfate:cyanide sulfurtransferase (rhodanese)	-0.0172	0.7050
JW1302	*ycjM*	Alpha amylase catalytic domain family protein	0.0532	0.1754
JW1303	*ycjN*	Putative ABC sugar transporter periplasmic binding protein	0.0096	0.6466
JW1304	*ycjO*	Putative sugar ABC transporter permease	-0.0013	0.9673
JW1305	*ycjP*	Putative sugar ABC transporter permease	0.0633	0.0427
JW1306	*ycjQ*	Putative Zn-dependent NAD(P)-binding oxidoreductase	-0.0048	0.8781
JW1308	*ycjS*	Putative NADH-binding oxidoreductase	-0.0741	0.0071

JW1309	*ycjT*	Putative family 65 glycosyl hydrolase	-0.0587	0.0839
JW1310	*ycjU*	Beta-phosphoglucomutase	0.1125	0.0229
JW1311	*ycjV*	CP4-44 prophage; putative disrupted hemin or colicin receptor	-0.0453	0.0355
JW1312	*ompG*	Outer membrane porin G	0.0877	0.0557
JW1313	*ycjW*	Laci family putative transcriptional repressor	0.0185	0.4799
JW1314	*ycjX*	DUF463 family protein, puatative P-loop ntpase	-0.0005	0.9913
JW1315	*ycjF*	UPF0283 family inner membrane protein	-0.0056	0.8849
JW1316	*tyrR*	Aromatic amino acid biosynthesis and transport regulon transcriptional regulator; autorepressor; atpase; phosphatase	0.0600	0.0352
JW1317	*tpx*	Lipid hydroperoxide peroxidase	-0.0381	0.3479
JW1318	*ycjG*	L-Ala-D/L-Glu epimerase	-0.0163	0.9003
JW1319	*mpaA*	Murein peptide amidase A	-0.1516	0.0005
JW1321	*ycjZ*	Murein peptide degradation regulator	-0.0352	0.4322
JW1322	*mppA*	Murein tripeptide (L-ala-gamma-D-glutamyl-meso-DAP) transporter subunit	-0.0535	0.0264
JW1323	*ynaI*	Low conductance mechanosensitive channel ynai	-0.0145	0.7326
JW1326	*ynaJ*	DUF2534 family putative inner membrane protein	-0.0259	0.2807
JW1327	*uspE*	Stress-induced protein	-0.1056	0.0032
JW1328	*fnr*	Oxygen-sensing anaerobic growth regulon transcriptional regulator FNR; autorepressor	0.1423	0.0143
JW1329	*ogt*	O-6-alkylguanine-DNA:cysteine-protein methyltransferase	-0.0351	0.3261
JW1331	*abgB*	P-aminobenzoyl-glutamate hydrolase, B subunit	0.0942	0.0723
JW1333	*abgR*	Putative DNA-binding transcriptional regulator of abgabt operon	-0.1100	0.0581
JW1334	*ydaL*	DNA endonuclease	-0.0759	0.2982
JW1336	*ydaN*	Putative Zn(II) transporter	0.0084	0.8283
JW1337	*dbpA*	ATP-dependent RNA helicase, specific for 23S rrna	0.0349	0.3160
JW1338	*ydaO*	Trna s(2)C32 thioltransferase, iron–sulfur cluster protein	-0.0366	0.3909
JW1339	*intR*	Rac prophage; integrase	-0.2696	0.0004
JW1341	*ydaC*	DUF1187 family protein, Rac prophage; putative double-strand break reduction protein	-0.0112	0.7825
JW1343	*recT*	Rac prophage; recombination and repair protein	0.0029	0.9581
JW1344	*recE*	Rac prophage; exonuclease VIII, 5' to 3' specific dsdna exonuclease	-0.0146	0.6390
JW1345	*racC*	Rac prophage; uncharacterized protein	-0.0119	0.5398
JW1346	*ydaE*	Conserved protein, Rac prophage	0.0320	0.1448
JW1347	*kil*	Killing protein, Rac prophage; ftsz inhibitor protein	0.0385	0.2431
JW1349	*ydaF*	Uncharacterized protein, Rac prophage	-0.0057	0.8759
JW1352	*ydaS*	Rac prophage; putative DNA-binding transcriptional regulator	0.0112	0.7545
JW1353	*ydaT*	Rac prophage; uncharacterized protein	-0.0285	0.3622

JW1354	*ydaU*	Rac prophage; conserved protein	0.0047	0.9208
JW1355	*ydaV*	Rac prophage; putative DNA replication protein	-0.0261	0.5896
JW1358	*trkG*	Rac prophage; potassium transporter subunit	0.0084	0.7672
JW1359	*ynaK*	Rac prophage; conserved protein	-0.0011	0.9757
JW1360	*ydaY*	Uncharacterized protein	0.0403	0.4029
JW1361	*ynaA*	Rac prophage; pseudogene, tail protein family	0.0953	0.1813
JW1366	*stfR*	Rac prophage; putative tail fiber protein	-0.0512	0.2429
JW1367	*tfaR*	Rac prophage; putative tail fiber assembly protein	-0.0101	0.7984
JW1368	*pinR*	Rac prophage; putative site-specific recombinase	0.0300	0.3706
JW1369	*ynaE*	Cold shock protein, Rac prophage	-0.0391	0.2355
JW1370	*uspF*	Stress-induced protein, ATP-binding protein	-0.0148	0.5473
JW1371	*ompN*	Outer membrane pore protein N, non-specific	-0.1022	0.0119
JW1372	*ydbK*	Pyruvate-flavodoxin oxidoreductase	0.0284	0.5607
JW1374	*hslJ*	Heat-inducible lipoprotein involved in novobiocin resistance	-0.0511	0.1451
JW1375	*ldhA*	Fermentative D-lactate dehydrogenase, NAD-dependent	0.0323	0.4492
JW1376	*ydbH*	Putative membrane-anchored protein, function unknown	-0.0099	0.7726
JW1377	*ynbE*	Lipoprotein	0.0583	0.0277
JW1379	*feaR*	Transcriptional activator for tyna and feab	-0.0731	0.1138
JW1380	*feaB*	Phenylacetaldehyde dehydrogenase	-0.0011	0.9717
JW1381	*tynA*	Tyramine oxidase, copper-requiring	-0.0294	0.4442
JW1382	*maoC*	Oxepin-coa hydrolase and 3-oxo-5,6-dehydrosuberyl-coa semialdehyde dehydrogenase	-0.0049	0.9222
JW1385	*paaC*	Ring 1,2-phenylacetyl-coa epoxidase subunit	0.0614	0.3654
JW1387	*paaE*	Ring 1,2-phenylacetyl-coa epoxidase, NAD(P)H oxidoreductase component	0.0370	0.3962
JW1388	*paaF*	2,3-dehydroadipyl-coa hydratase	0.0554	0.1882
JW1389	*paaG*	1,2-epoxyphenylacetyl-coa isomerase, oxepin-coa-forming	-0.0531	0.0252
JW1390	*paaH*	3-hydroxyadipyl-coa dehydrogenase, NAD+-dependent	-0.0679	0.0289
JW1392	*paaJ*	3-oxoadipyl-coa/3-oxo-5,6-dehydrosuberyl-coa thiolase	-0.0315	0.4184
JW1394	*paaX*	Transcriptional repressor of phenylacetic acid degradation paa operon, phenylacetyl-coa inducer	0.0515	0.3691
JW1395	*paaY*	Thioesterase required for phenylacetic acid degradation; trimeric; phenylacetate regulatory and detoxification protein; hexapeptide repeat protein	-0.0173	0.5388
JW1402	*ydbA*	CP4-44 prophage; putative disrupted hemin or colicin receptor	0.0002	0.9937
JW1403	*ydbC*	Pyridoxine 4-dehydrogenase	0.0310	0.3878
JW1405	*ynbA*	Inner membrane protein	0.0110	0.6868
JW1406	*ynbB*	Putative CDP-diglyceride synthase	-0.0033	0.9247

JW1407	*ynbC*	Putative esterase	0.0362	0.2652
JW1408	*ynbD*	Putative phosphatase inner membrane protein	-0.0809	0.0811
JW1409	*azoR*	NADH-azoreductase, FMN-dependent	0.0198	0.5474
JW1411	*ydcF*	DUF218 superfamily protein, SAM-binding	0.0422	0.1710
JW1412	*aldA*	Aldehyde dehydrogenase A, NAD-linked	0.0250	0.3960
JW1413	*gapC*	CP4-44 prophage; putative disrupted hemin or colicin receptor	-0.0205	0.5373
JW1416	*ydcA*	Putative periplasmic protein	0.0557	0.2708
JW1419	*ydcJ*	Putative metalloenzyme	-0.0110	0.7416
JW1420	*mdoD*	OPG biosynthetic periplasmic protein	0.0851	0.0711
JW1423	*rimL*	Ribosomal-protein-L7/L12-serine acetyltransferase	-0.0426	0.1302
JW1424	*ydcK*	Uncharacterized protein	-0.1063	0.0190
JW1425	*tehA*	Potassium-tellurite ethidium and proflavin transporter	0.0027	0.9417
JW1426	*tehB*	Tellurite, selenium methyltransferase, SAM-dependent; tellurite, selenium resistance protein	0.0151	0.2225
JW1427	*ydcL*	Lipoprotein	-0.0835	0.0274
JW1430	*ydcN*	Putative DNA-binding transcriptional regulator	0.0321	0.3128
JW1431	*ydcP*	Putative peptidase	-0.0217	0.5795
JW1432	*yncJ*	Uncharacterized protein	0.0697	0.0080
JW1433	*ydcQ*	Antitoxin for the hicab toxin-antitoxin system	0.0293	0.4044
JW1434	*ydcR*	Putative DNA-binding transcriptional regulator and putative aminotransferase	0.0282	0.3238
JW1436	*ydcT*	Putative ABC transporter atpase	-0.0125	0.7765
JW1437	*ydcU*	Putative ABC transporter permease	0.0664	0.0410
JW1438	*ydcV*	Putative ABC transporter permease	0.0113	0.4866
JW1439	*ydcW*	Gamma-aminobutyraldehyde dehydrogenase	0.0547	0.3007
JW1441	*ydcY*	DUF2526 family protein	0.0140	0.6414
JW1442	*ydcZ*	DUF606 family inner membrane protein	-0.0006	0.9842
JW1445	*yncC*	Colanic acid and biofilm gene transcriptional regulator, mqsr-controlled	-0.0482	0.0883
JW1446	*yncD*	Putative iron outer membrane transporter	-0.0166	0.7234
JW1447	*yncE*	ATP-binding protein, periplasmic, function unknown	0.0592	0.0005
JW1449	*yncG*	Glutathione S-transferase homolog	-0.0271	0.5693
JW1451	*rhsE*	Pseudogene, Rhs family	0.0168	0.6311
JW1452	*ydcD*	Putative immunity protein for rhse	-0.0833	0.0017
JW1453	*yncI*	CP4-44 prophage; putative disrupted hemin or colicin receptor	-0.0009	0.9789
JW1455	*ydcC*	H repeat-associated putative transposase	0.0782	0.0111
JW1456	*ydcE*	4-oxalocrotonate tautomerase	0.0598	0.6922
JW1457	*yddH*	Flavin reductase like-protein	-0.0732	0.1401
JW1458	*nhoA*	N-hydroxyarylamine O-acetyltransferase	0.0125	0.7433
JW1459	*yddE*	Phzc-phzf family protein	0.0188	0.7041
JW1460	*narV*	Nitrate reductase 2 (NRZ), gamma subunit	-0.0052	0.9255

JW1463	*narZ*	Nitrate reductase 2 (NRZ), alpha subunit	-0.0794	0.1096
JW1464	*narU*	Nitrate/nitrite transporter	-0.0238	0.4654
JW1466	*yddJ*	Uncharacterized protein	0.0331	0.5053
JW1467	*yddK*	Pseudogene, leucine-rich protein	0.0656	0.1600
JW1468	*yddL*	Putative lipoprotein	0.2727	0.0694
JW1469	*yddG*	Aromatic amino acid exporter	0.1695	0.2040
JW1470	*fdnG*	Formate dehydrogenase, nitrate-inducible, major subunit	-0.0289	0.4305
JW1471	*fdnH*	Formate dehydrogenase-N, Fe-S (beta) subunit, nitrate-inducible	0.0120	0.7973
JW1472	*fdnI*	Formate dehydrogenase-N, cytochrome B556 (gamma) subunit, nitrate-inducible	-0.2512	0.0001
JW1474	*adhP*	Ethanol-active dehydrogenase/acetaldehyde-active reductase	0.0724	0.0675
JW1478	*ddpF*	D,D-dipeptide ABC transporter atpase	-0.1044	0.1098
JW1479	*ddpD*	D,D-dipeptide ABC transporter atpase	-0.0077	0.8674
JW1480	*ddpC*	D,D-dipeptide ABC transporter permease	0.0136	0.7085
JW1481	*ddpB*	D,D-dipeptide ABC transporter permease	-0.0683	0.0711
JW1483	*ddpX*	D-ala-D-ala dipeptidase, Zn-dependent	0.0363	0.3180
JW1484	*dos*	Oxygen sensor, c-di-GMP phosphodiesterase, heme-regulated; cold- and stationary phase-induced bioflim regulator	0.0110	0.6895
JW1486	*yddW*	Liprotein, glycosyl hydrolase homolog	0.0486	0.2375
JW1487	*gadC*	Glutamate:gamma-aminobutyric acid antiporter	-0.0232	0.1687
JW1488	*gadB*	Glutamate decarboxylase B, PLP-dependent	0.1821	0.0124
JW1489	*pqqL*	Putative periplasmic M16 family zinc metalloendopeptidase	0.0324	0.5333
JW1490	*yddB*	Putative tonb-dependent outer membrane receptor	0.0136	0.6018
JW1492	*ydeM*	Putative yden-specific sulfatase-maturating enzyme	0.0329	0.4601
JW1494	*ydeO*	UV-inducible global regulator, evga-, gade-dependent	0.0300	0.4783
JW1495	*ydeP*	Putative oxidoreductase	0.0135	0.6889
JW1496	*ydeQ*	Putative fimbrial-like adhesin protein	0.0266	0.4031
JW1497	*ydeR*	Putative fimbrial-like adhesin protein	-0.0543	0.0243
JW1498	*ydeS*	Putative fimbrial-like adhesin protein	0.0352	0.2792
JW1499	*ydeT*	Pseudogene	-0.0245	0.5725
JW1500	*hipA*	Inactivating gltx kinase facilitating persister formation; toxin of hipab TA pair; autokinase	0.0854	0.0542
JW1501	*hipB*	Antitoxin of hipab toxin-antitoxin system	-0.0184	0.6414
JW1502	*ydeU*	Uncharacterized protein	-0.0369	0.4874
JW1503	*ydeK*	Pseudogene, aida homolog	-0.0143	0.6628
JW1504	*ydeV*	Autoinducer-2 (AI-2) kinase	0.1096	0.0080
JW1505	*ydeW*	Lsr operon transcriptional repressor	-0.0399	0.1711
JW1506	*ego*	Autoinducer 2 import ATP-binding protein	-0.0784	0.0321
JW1507	*lsrC*	Autoinducer 2 import system permease protein	0.0613	0.1015

JW1508	*lsrD*	Autoinducer 2 import system permease protein	0.0420	0.4826
JW1509	*lsrB*	Autoinducer 2-binding protein	0.0577	0.1275
JW1510	*lsrF*	Putative autoinducer-2 (AI-2) aldolase	0.0826	0.0694
JW1511	*lsrG*	Autoinducer-2 (AI-2) degrading protein lsrg	-0.0846	0.0617
JW1512	*tam*	Trans-aconitate methyltransferase	-0.0907	0.0402
JW1514	*uxaB*	Altronate oxidoreductase, NAD-dependent	0.0135	0.6875
JW1516	*yneG*	DUF4186 family protein	0.1255	0.3303
JW1517	*yneH*	Glutaminase 2	0.0633	0.1038
JW1519	*yneJ*	Putative DNA-binding transcriptional regulator	-0.0103	0.8398
JW1520	*yneK*	Uncharacterized protein	-0.0374	0.5094
JW1521	*ydeA*	Arabinose efflux transporter, arabinose-inducible	-0.0030	0.9113
JW1522	*marC*	UPF0056 family inner membrane protein	-0.0529	0.0996
JW1525	*marB*	Periplasmic mar operon regulator	-0.0451	0.3165
JW1527	*ydeE*	Putative transporter	0.0585	0.0461
JW1528	*ydeH*	Diguanylate cyclase, zinc-sensing	0.0288	0.2718
JW1529	*ydeI*	Hydrogen peroxide resistance OB fold protein; putative periplasmic protein	-0.0285	0.2972
JW1530	*ydeJ*	Inactive pncc family protein	0.0884	0.0005
JW1531	*dcp*	Dipeptidyl carboxypeptidase II	0.0214	0.5346
JW1532	*ydfG*	NADP-dependent 3-hydroxy acid dehydrogenase; malonic semialdehyde reductase	-0.0039	0.9347
JW1533	*ydfH*	Transcriptional repressor for rspab	-0.0123	0.7938
JW1534	*ydfZ*	Selenoprotein, function unknown	-0.0297	0.5193
JW1535	*ydfI*	Putative NAD-dependent D-mannonate oxidoreductase	0.0294	0.4184
JW1536	*ydfJ*	Pseudogene, MFS transporter family; interrupted by Qin prophage	0.0173	0.5369
JW1537	*ydfK*	Cold shock protein, function unknown, Qin prophage	0.1202	0.0168
JW1538	*pinQ*	Qin prophage; putative site-specific recombinase	-0.0355	0.5170
JW1539	*tfaQ*	Qin prophage; putative tail fibre assembly protein	-0.0237	0.7199
JW1540	*stfQ*	Qin prophage; putative side tail fibre assembly protein	-0.0430	0.0225
JW1541	*nohA*	Pseudogene, Qin prophage; Phage DNA packaging protein Nu1 family	-0.0237	0.6479
JW1545	*ydfP*	Qin prophage; Rz-like protein	-0.0237	0.8452
JW1546	*ydfQ*	Qin prophage; putative lysozyme	0.0029	0.8618
JW1547	*ydfR*	Qin prophage; DUF1327 family protein	-0.0036	0.9384
JW1549	*cspB*	Qin prophage; cold shock protein	-0.0019	0.9508
JW1550	*cspF*	Qin prophage; cold shock protein	-0.1603	0.2667
JW1551	*ydfT*	Qin prophage; putative antitermination protein Q	0.0147	0.6673
JW1553	*rem*	Qin prophage; uncharacterized protein	0.0285	0.5443
JW1554	*hokD*	Qin prophage; small toxic polypeptide	0.1041	0.0000
JW1555	*relE*	Qin prophage; toxin of the rele-relb toxin-antitoxin system	0.0713	0.0198

JW1556	*relB*	Antitoxin of the rele-relb toxin-antitoxin syste; transcriptional repressor	0.0365	0.2847
JW1557	*ydfV*	Qin prophage; uncharacterized protein	-0.0314	0.0765
JW1558	*flxA*	Qin prophage; uncharacterized protein	0.0512	0.0748
JW1559	*ydfW*	Pseudogene, integrase fragment, Qin prophage	0.0100	0.8163
JW1560	*ydfX*	Pseudogene, Qin prophage	0.0852	0.0086
JW1563	*ydfA*	Qin prophage; DUF1391 family protein	-0.0150	0.5992
JW1565	*ydfC*	Qin prophage; uncharacterized protein	-0.0024	0.9254
JW1567	*ydfD*	Qin prophage; DUF1482 family protein	0.0316	0.3731
JW1568	*ydfE*	Qin prophage; pseudogene	0.0524	0.1204
JW1571	*intQ*	Pseudogene, Qin prophage; phage integrase family	0.0415	0.4203
JW1572	*rspB*	Putative Zn-dependent NAD(P)-binding oxidoreductase	0.0583	0.0474
JW1573	*rspA*	Bifunctional D-altronate/D-mannonate dehydratase	-0.0004	0.9924
JW1574	*ynfA*	UPF0060 family inner membrane protein	0.1294	0.0344
JW1575	*ynfB*	UPF0482 family putative periplasmic protein	-0.0525	0.4251
JW1576	*speG*	Spermidine N(1)-acetyltransferase	0.2012	0.0085
JW1581	*ynfG*	Oxidoreductase, Fe-S subunit	0.0410	0.2950
JW1586	*dgsA*	Glucosamine anaerobic growth regulon transcriptional repressor; autorepressor	0.0009	0.0271
JW1587	*ynfL*	Lysr family putative transcriptional regulator	0.0102	0.8536
JW1588	*ynfM*	Putative arabinose efflux transporter	-0.0185	0.6708
JW1590	*ydgD*	Putative peptidase	-0.0015	0.9618
JW1591	*mdtI*	Multidrug efflux system transporter	-0.0246	0.2225
JW1592	*mdtJ*	Multidrug efflux system transporter	-0.0930	0.0048
JW1593	*ydgG*	Pheromone AI-2 transporter	0.0967	0.1715
JW1594	*pntB*	Pyridine nucleotide transhydrogenase, beta subunit	0.0487	0.3965
JW1595	*pntA*	Pyridine nucleotide transhydrogenase, alpha subunit	0.1253	0.0633
JW1596	*ydgH*	DUF1471 family periplasmic protein	0.0510	0.0078
JW1597	*ydgI*	Putative arginine/ornithine antiporter transporter	-0.0242	0.4821
JW1598	*folM*	Dihydromonapterin reductase, NADPH-dependent; dihydrofolate reductase isozyme	0.0337	0.6581
JW1599	*ydgC*	Glpm family inner membrane protein	-0.0237	0.4920
JW1600	*rstA*	Response regulator of rstab two-component system	0.0336	0.4491
JW1601	*rstB*	Sensory histidine kinase of rstab two-component system	-0.0100	0.8834
JW1602	*tus*	Inhibitor of replication at Ter, DNA-binding protein	0.0458	0.4309
JW1603	*fumC*	Fumarate hydratase (fumarase C),aerobic Class II	-0.1626	0.0185
JW1604	*fumA*	Fumarate hydratase (fumarase A), aerobic Class I	0.0781	0.0434
JW1606	*ydgA*	DUF945 family protein	0.0305	0.2488

JW1607	*uidC*	Putative outer membrane porin for beta-glucuronides porin protein	0.0265	0.4313
JW1608	*uidB*	Glucuronide transporter	-0.1070	0.0013
JW1609	*uidA*	Beta-D-glucuronidase	-0.0778	0.1656
JW1610	*uidR*	Transcriptional repressor	-0.0149	0.6095
JW1611	*hdhA*	7-alpha-hydroxysteroid dehydrogenase, NAD-dependent	-0.0661	0.1613
JW1612	*malI*	Transcriptional repressor of Mal regulon	0.0351	0.4235
JW1613	*malX*	Maltose and glucose-specific PTS enzyme IIB component and IIC component	-0.0081	0.8252
JW1614	*malY*	PLP-dependent beta-cystathionase and maltose regulon regulator	0.0143	0.7829
JW1615	*add*	Adenosine deaminase	0.0925	0.0046
JW1617	*ydgT*	Nucleoid-associated oric-binding protein; H-NS and stpa stabilizing factor	0.0341	0.1752
JW1618	*ydgK*	Inner membrane protein ydgk	-0.0302	0.3271
JW1619	*rsxA*	Soxr iron-sulfur cluster reduction factor component; inner membrane protein of electron transport complex	-0.1181	0.0006
JW1620	*rsxB*	Soxr iron-sulfur cluster reduction factor component; putative iron-sulfur protein	-0.0057	0.8759
JW1621	*rsxC*	Soxr iron-sulfur cluster reduction factor component; putative membrane-associated NADH oxidoreductase of electron transport complex	0.0218	0.4098
JW1622	*rsxD*	Soxr iron-sulfur cluster reduction factor component; putative membrane protein of electron transport complex	-0.0520	0.0856
JW1623	*rsxG*	Soxr iron-sulfur cluster reduction factor component; putative membrane protein of electron transport complex	-0.1489	0.0011
JW1624	*rsxE*	Soxr iron-sulfur cluster reduction factor component; electron transport inner membrane NADH-quinone reductase	0.0695	0.0151
JW1625	*nth*	DNA glycosylase and apyrimidinic (AP) lyase (endonuclease III)	0.0778	0.0400
JW1626	*ydgR*	Dipeptide and tripeptide permease A	-0.0309	0.3721
JW1627	*gst*	Glutathionine S-transferase	-0.0418	0.2068
JW1628	*pdxY*	Pyridoxamine kinase	0.0178	0.3505
JW1630	*pdxH*	Pyridoxine 5'-phosphate oxidase	-0.0397	0.0613
JW1631	*ydhA*	Inhibitor of c-type lysozyme, membrane-bound; putative lipoprotein	0.0757	0.0067
JW1632	*ydhH*	Anhydro-N-acetylmuramic acid kinase	-0.0580	0.2660
JW1635	*ydhI*	DUF1656 family putative inner membrane efflux pump associated protein	0.1084	0.0017
JW1636	*ydhJ*	Putative membrane fusion protein (MFP) of ydhjk efflux pump	-0.0274	0.3299
JW1637	*ydhK*	Putative efflux protein (PET) component of ydhjk efflux pump	-0.0414	0.2313
JW1638	*sodC*	Superoxide dismutase, Cu, Zn, periplasmic	-0.1655	0.0181

JW1639	*ydhF*	Putative oxidoreductase	-0.0032	0.8743
JW1642	*nemA*	Chromate reductase, quinone reductase, FMN-linked; N-Ethylmaleimide reductase; old yellow enzyme	0.0461	0.0165
JW1643	*gloA*	Glyoxalase I, Ni-dependent	-0.0256	0.4461
JW1644	*rnt*	Rnase T; exoribonuclease T; structured DNA 3' exonuclease; RNA processing; DNA repair	-0.3221	0.0000
JW1645	*lhr*	Putative ATP-dependent helicase	-0.0167	0.7110
JW1646	*ydhD*	Glutaredoxin-4	-0.2656	0.0001
JW1648	*sodB*	Superoxide dismutase, Fe	0.0569	0.4067
JW1649	*ydhP*	Putative MFS transporter, inner membrane protein	0.1055	0.0083
JW1650	*purR*	Transcriptional repressor, hypoxanthine-binding	0.1169	0.0283
JW1651	*ydhB*	Lysr family putative transcriptional regulator	0.0131	0.7844
JW1652	*ydhC*	Putative arabinose efflux transporter	0.0202	0.7670
JW1653	*cfa*	Cyclopropane fatty acyl phospholipid synthase, SAM-dependent	-0.0208	0.4369
JW1655	*mdtK*	Multidrug efflux system transporter	-0.0050	0.8553
JW1656	*ydhQ*	Autotransporter adhesin-related protein	0.0797	0.0880
JW1657	*ydhR*	Putative monooxygenase	-0.1842	0.0016
JW1658	*ydhS*	Uncharacterized protein	0.0272	0.3298
JW1659	*ydhT*	FNR, Nar, narp-regulated protein; putative subunit of ydhyvwxut oxidoreductase complex	0.0117	0.6389
JW1662	*ydhW*	FNR, Nar, narp-regulated protein; putative subunit of ydhyvwxut oxidoreductase complex	0.0235	0.6200
JW1664	*ydhY*	Putative 4Fe-4S ferridoxin-type protein; FNR, Nar, narp-regulated protein; putative subunit of ydhyvwxut oxidoreductase complex	0.0404	0.2439
JW1665	*ydhZ*	Fumarase D	-0.0258	0.5199
JW1666	*pykF*	Pyruvate kinase I	0.1693	0.0035
JW1667	*lpp*	Murein lipoprotein	-0.0109	0.6795
JW1668	*ynhG*	Murein L,D-transpeptidase	0.0576	0.2273
JW1669	*sufE*	Sulfur acceptor protein	-0.1495	0.0136
JW1670	*sufS*	Cysteine desulfurase, stimulated by sufe; selenocysteine lyase, PLP-dependent	-0.1426	0.0087
JW1671	*sufD*	Component of sufbcd Fe-S cluster assembly scaffold	-0.0666	0.1020
JW1672	*sufC*	Sufbcd Fe-S cluster assembly scaffold protein, ATP-binding protein	-0.0780	0.0365
JW1674	*sufA*	Fe-S cluster assembly protein	0.0031	0.8984
JW1675	*ydiH*	Uncharacterized protein	-0.0089	0.8021
JW1676	*ydiI*	1,4-dihydroxy-2-naphthoyl-coa hydrolase	-0.0251	0.5955
JW1677	*ydiJ*	Putative FAD-linked oxidoreductase	-0.0445	0.1759
JW1678	*ydiK*	UPF0118 family inner membrane protein	-0.0394	0.3928
JW1680	*ydiM*	Inner membrane transport protein ydim	-0.0125	0.7358
JW1682	*ydiB*	CP4-44 prophage; putative disrupted hemin or colicin receptor	0.0851	0.0477

JW1683	*aroD*	3-dehydroquinate dehydratase	-0.0724	0.0343
JW1686	*ydiP*	Putative DNA-binding transcriptional regulator	0.0235	0.3558
JW1688	*ydiR*	Putative electron transfer flavoprotein, FAD-binding subunit	0.1014	0.0346
JW1689	*ydiS*	Putative oxidoreductase	0.0446	0.2340
JW1690	*ydiT*	Ferredoxin-like protein ydit	-0.1302	0.1236
JW1692	*pps*	Phosphoenolpyruvate synthase	-0.0892	0.0716
JW1693	*ydiA*	PEP synthase kinase and PEP synthase pyrophosphorylase	-0.0125	0.8013
JW1695	*ydiE*	Hemin uptake protein hemp homolog	-0.0273	0.4701
JW1696	*ydiU*	UPF0061 family protein	0.1131	0.0503
JW1697	*ydiV*	Anti-flhd4c2 factor, inactive EAL family phosphodiesterase	0.0525	0.1490
JW1699	*btuD*	Vitamin B12 ABC transporter atpase	0.0629	0.0391
JW1700	*btuE*	Glutathione peroxidase	-0.0507	0.2978
JW1701	*btuC*	Vitamin B12 ABC transporter permease	-0.0391	0.2788
JW1702	*ihfA*	Integration host factor (IHF), DNA-binding protein, alpha subunit	-0.0256	0.5730
JW1705	*pheM*	Phenylalanyl-trna synthetase operon leader peptide	0.0240	0.5791
JW1707	*rpmI*	50S ribosomal protein L35	0.0053	0.8239
JW1710	*arpB*	CP4-44 prophage; putative disrupted hemin or colicin receptor	-0.0944	0.0165
JW1711	*ydiY*	Acid-inducible putative outer membrane protein	-0.0157	0.5364
JW1713	*ydiZ*	Uncharacterized protein	0.0542	0.1233
JW1714	*yniA*	Fructosamine kinase family protein	0.0861	0.0301
JW1715	*yniB*	Putative inner membrane protein	-0.0018	0.9556
JW1716	*yniC*	Hexitol phosphatase B; 2-deoxyglucose-6-P phosphatase	0.0728	0.0880
JW1718	*ydjN*	Putative transporter	-0.0509	0.1327
JW1719	*ydjO*	Uncharacterized protein	-0.0127	0.7854
JW1721	*katE*	Catalase HPII, heme d-containing	0.0869	0.0622
JW1722	*chbG*	Chito-oligosaccharide deacetylase	0.0152	0.6754
JW1723	*chbF*	Phospho-chitobiase; general 6-phospho-beta-glucosidase activity	0.1122	0.0481
JW1724	*chbR*	Repressor of chb operon for N,N'-diacetylchitobiose utilization	-0.0435	0.4242
JW1725	*chbA*	N,N'-diacetylchitobiose-specific enzyme IIA component of PTS	0.0264	0.3828
JW1726	*chbC*	N,N'-diacetylchitobiose-specific enzyme IIC component of PTS	-0.0801	0.0884
JW1727	*chbB*	N,N'-diacetylchitobiose-specific enzyme IIB component of PTS	0.0196	0.5544
JW1730	*ydjQ*	Endonuclease of nucleotide excision repair	0.0636	0.2695
JW1731	*ydjR*	Cold- and stress-inducible protein	0.0256	0.4089
JW1732	*spy*	Periplasmic ATP-independent protein refolding chaperone, stress-induced	-0.0154	0.5393
JW1733	*astE*	Succinylglutamate desuccinylase	-0.0507	0.3962

JW1734	*astB*	Succinylarginine dihydrolase	0.0310	0.4681
JW1736	*astA*	Arginine succinyltransferase	0.0313	0.3080
JW1737	*astC*	Succinylornithine transaminase, PLP-dependent	-0.0562	0.1087
JW1738	*xthA*	Exonuclease III	0.0578	0.1686
JW1739	*ydjX*	TVP38/TMEM64 family inner membrane protein	0.0827	0.0196
JW1741	*ydjZ*	TVP38/TMEM64 family inner membrane protein	-0.0292	0.2951
JW1742	*ynjA*	Carboxymuconolactone decarboxylase family protein	0.1221	0.0090
JW1747	*ynjF*	CDP-alcohol phosphatidyltransferase family inner membrane protein	-0.0565	0.3031
JW1748	*nudG*	CTP pyrophosphohydrolase; also hydrolyzes 2-hydroxy-datp, 8-hydroxy-dgtp, 5-hydroxy-CTP, dctp and 5-methyl-dctp	-0.0612	0.2189
JW1749	*ynjH*	DUF1496 family protein	-0.0473	0.0470
JW1750	*gdhA*	Glutamate dehydrogenase, NADP-specific	0.1497	0.0679
JW1752	*topB*	DNA topoisomerase III	-0.0174	0.6203
JW1753	*selD*	Selenophosphate synthase	-0.0018	0.9648
JW1754	*ydjA*	Putative oxidoreductase	-0.0116	0.7243
JW1755	*sppA*	Protease IV (signal peptide peptidase)	0.0402	0.3145
JW1756	*ansA*	Cytoplasmic L-asparaginase 1	0.0509	0.0772
JW1757	*pncA*	Nicotinamidase/pyrazinamidase	-0.0639	0.0754
JW1758	*ydjE*	Putative MFS sugar transporter, membrane protein	-0.1059	0.0461
JW1759	*ydjF*	Putative DNA-binding transcriptional regulator	0.1066	0.0157
JW1760	*ydjG*	Methylglyoxal reductase, NADH-dependent	-0.0589	0.0990
JW1762	*ydjI*	Putative aldolase	-0.0083	0.7903
JW1763	*ydjJ*	Putative Zn-dependent NAD(P)-binding oxidoreductase	0.0746	0.1494
JW1765	*ydjL*	Putative Zn-dependent NAD(P)-binding oxidoreductase	0.0356	0.2807
JW1766	*yeaC*	DUF1315 family protein	-0.0006	0.9855
JW1767	*yeaA*	Methionine sulfoxide reductase B	0.0530	0.1710
JW1769	*yeaD*	D-hexose-6-phosphate epimerase-like protein	-0.0377	0.4061
JW1770	*yeaE*	Aldo-keto reductase, methylglyoxal to acetol, NADPH-dependent	0.0252	0.5374
JW1771	*mipA*	Scaffolding protein for murein synthesizing machinery	-0.0194	0.8016
JW1772	*yeaG*	Protein kinase, endogenous substrate unidentified; autokinase	-0.0144	0.6906
JW1773	*yeaH*	UPF0229 family protein	0.0490	0.0084
JW1774	*yeaI*	Inactive diguanylate cyclase	0.0816	0.1006
JW1776	*yeaK*	Aminoacyl-trna editing domain protein	0.0137	0.7622
JW1778	*yeaL*	UPF0756 family putative inner membrane protein	-0.0240	0.1346
JW1779	*yeaM*	Putative DNA-binding transcriptional regulator	-0.0263	0.4888
JW1780	*yeaN*	Putative MFS transporter, inner membrane protein	-0.0332	0.4319
JW1781	*yeaO*	DUF488 family protein	0.0149	0.6899

JW1782	*yoaF*	DUF333 family outer membrane lipoprotein	-0.0039	0.9121
JW1784	*yeaQ*	UPF0410 family protein	-0.0132	0.6418
JW1786	*yeaR*	DUF1971 family protein, nitrate-inducible	-0.0255	0.4652
JW1787	*yeaS*	Leucine efflux protein	-0.0620	0.1525
JW1789	*yeaU*	D-malate oxidase, NAD-dependent; putative tartrate dehydrogenase	-0.4181	0.0004
JW1793	*rnd*	Ribonuclease D	-0.0656	0.1277
JW1794	*fadD*	Acyl-coa synthetase (long-chain-fatty-acid--coa ligase)	0.0058	0.8617
JW1795	*yeaY*	Slp family lipoprotein, rpoe-regulated	0.0127	0.7361
JW1797	*yoaA*	Putative ATP-dependent helicase, ding family	0.0082	0.8647
JW1800	*yoaH*	UPF0181 protein yoah	0.0093	0.6912
JW1801	*pabB*	Aminodeoxychorismate synthase, subunit I	-0.0686	0.0028
JW1802	*yeaB*	Putative coa pyrophosphohydrolase, weak 3-phosphohydroxypyruvate phosphatase	0.0138	0.5863
JW1803	*sdaA*	L-serine dehydratase 1	-0.0313	0.3834
JW1804	*yoaD*	Putative membrane-anchored cyclic-di-GMP phosphodiesterase, regulator of cellulose production	0.0440	0.3380
JW1805	*yoaE*	Putative membrane protein/conserved protein	0.0478	0.1018
JW1806	*manX*	Fused mannose-specific PTS enzymes: IIA component/IIB component	-0.0230	0.4807
JW1807	*manY*	Mannose-specific enzyme IIC component of PTS	0.0817	0.0013
JW1808	*manZ*	Mannose-specific enzyme IID component of PTS	-0.0486	0.2751
JW1809	*yobD*	UPF0266 family inner membrane protein	-0.0919	0.0168
JW1811	*rrmA*	23S rrna m(1)G745 methyltransferase, SAM-dependent	0.1332	0.0001
JW1812	*cspC*	Stress protein, member of the cspa-family	0.0015	0.0408
JW1813	*yobF*	DUF2527 family heat-induced protein	-0.0115	0.8337
JW1814	*yebO*	Putative inner membrane protein	0.0142	0.6508
JW1815	*yobG*	Regulatory peptide for phopq, feedback inhibition	-0.0605	0.1150
JW1816	*kdgR*	KDG regulon transcriptional repressor	-0.0317	0.4548
JW1818	*htpX*	Putative endopeptidase	0.0335	0.4153
JW1819	*prc*	Carboxy-terminal protease for penicillin-binding protein 3	0.0925	0.0359
JW1821	*yebR*	Free methionine-(R)-sulfoxide reductase	0.0343	0.1492
JW1822	*yebS*	Inner membrane subunit of the putative yebst transporter; pqia domain protein	-0.0062	0.8625
JW1827	*pphA*	Serine/threonine-specific protein phosphatase 1	-0.1978	0.0000
JW1828	*yebY*	DUF2511 family protein	0.0446	0.2308
JW1829	*yebZ*	Inner membrane protein	0.0091	0.8247
JW1830	*yobA*	Copc family protein	0.0409	0.1912
JW1831	*holE*	DNA polymerase III, theta subunit	0.0674	0.0092
JW1832	*yobB*	C-N hydrolase family protein	0.0414	0.0913
JW1833	*exoX*	Exodeoxyribonuclease 10; DNA exonuclease X	-0.0903	0.1903

JW1834	*ptrB*	Protease II	0.0644	0.2264
JW1835	*yebE*	DUF533 family inner membrane protein	0.0390	0.2160
JW1836	*yebF*	Extracellular Colicin M immunity family protein	-0.2683	0.0000
JW1837	*yebG*	DNA damage-inducible protein regulated by lexa	-0.1268	0.0331
JW1838	*purT*	Phosphoribosylglycinamide formyltransferase 2	-0.2156	0.0009
JW1839	*eda*	KHG/KDPG aldolase; 2-dehydro-3-deoxy-phosphogluconate/4-hydroxy-2-oxoglutarate aldolase	-0.0377	0.5030
JW1840	*edd*	6-phosphogluconate dehydratase	-0.0214	0.6641
JW1841	*zwf*	Glucose-6-phosphate 1-dehydrogenase	-0.0314	0.4993
JW1842	*yebK*	Putative DNA-binding transcriptional regulator	0.0172	0.7604
JW1843	*pykA*	Pyruvate kinase II	-0.0427	0.2399
JW1847	*znuC*	Zinc ABC transporter atpase	-0.3609	0.0008
JW1848	*znuB*	Zinc ABC transporter permease	-0.0589	0.1908
JW1849	*ruvB*	ATP-dependent DNA helicase, component of ruvabc resolvasome	0.0022	0.9715
JW1850	*ruvA*	Component of ruvabc resolvasome, regulatory subunit	-0.0839	0.2911
JW1852	*ruvC*	Component of ruvabc resolvasome, endonuclease	-0.1836	0.0059
JW1853	*yebC*	UPF0082 family protein	-0.1362	0.0236
JW1854	*nudB*	Dihydroneopterin triphosphate pyrophosphatase	-0.1214	0.0675
JW1857	*yecE*	UPF0759 family protein	0.0015	0.9691
JW1859	*yecO*	Carboxy-SAM synthase	0.0853	0.2814
JW1860	*yecP*	Trna U34 carboxymethyltransferase	0.0562	0.3839
JW1861	*torZ*	Trimethylamine N-oxide reductase system III, catalytic subunit	-0.0282	0.3289
JW1862	*torY*	TMAO reductase III (toryz), cytochrome c-type subunit	-0.0195	0.6679
JW1863	*cutC*	Putative copper homeostasis protein	-0.0371	0.0980
JW1867	*flhE*	Proton seal during flagellar secretion	0.0539	0.1178
JW1868	*flhA*	Putative flagellar export pore protein	0.0073	0.8689
JW1869	*flhB*	Flagellin export apparatus, substrate specificity protein	-0.0750	0.0751
JW1870	*cheZ*	Chemotaxis regulator, protein phosphatase for chey	-0.0217	0.6429
JW1871	*cheY*	Chemotaxis regulator transmitting signal to flagellar motor component	0.0481	0.0099
JW1872	*cheB*	Fused chemotaxis regulator: protein-glutamate methylesterase in two-component regulatory system with chea	0.1551	0.0361
JW1873	*cheR*	Chemotaxis regulator, protein-glutamate methyltransferase	-0.0747	0.1379
JW1875	*tar*	Methyl-accepting chemotaxis protein II	0.0233	0.2533
JW1876	*cheW*	Purine-binding chemotaxis protein	0.0550	0.0575
JW1877	*cheA*	Fused chemotactic sensory histidine kinase in two-component regulatory system with cheb and chey: sensory histidine kinase/signal sensing protein	0.0481	0.1281

JW1880	*flhC*	Flagellar class II regulon transcriptional activator, with flhd	0.0203	0.3861
JW1881	*flhD*	Flagellar class II regulon transcriptional activator, with flhc	-0.0400	0.2642
JW1884	*yecG*	Universal stress protein	-0.0499	0.3630
JW1887	*araH*	L-arabinose ABC transporter permease	-0.0064	0.8622
JW1888	*araG*	L-arabinose ABC transporter atpase	-0.0032	0.9495
JW1889	*araF*	L-arabinose ABC transporter periplasmic binding protein	0.0281	0.3733
JW1890	*yecI*	Ferritin B, putative ferrous iron reservoir	0.0289	0.3868
JW1891	*yecJ*	DUF2766 family protein	0.0311	0.5885
JW1892	*yecR*	Lipoprotein, function unknown	-0.0740	0.0060
JW1893	*ftn*	Ferritin iron storage protein (cytoplasmic)	-0.0008	0.9835
JW1894	*yecH*	DUF2492 family protein	-0.1310	0.0141
JW1895	*tyrP*	Tyrosine transporter	-0.0044	0.8626
JW1896	*yecA*	UPF0149 family protein	-0.1144	0.0055
JW1898	*uvrC*	Excinuclease uvrabc, endonuclease subunit	0.0296	0.3681
JW1899	*uvrY*	Response regulator in two-component regulatory system with bara	0.0573	0.0648
JW1900	*yecF*	DUF2594 family protein	0.0898	0.0169
JW1901	*sdiA*	Quorum-sensing transcriptional activator	0.0430	0.3306
JW1902	*yecC*	Putative ABC transporter atpase	0.0052	0.8689
JW1903	*yecS*	ABC family putative inner membrane permease	0.0358	0.6318
JW1906	*fliZ*	Rpos antagonist; putative regulator of flia activity	-0.0554	0.1624
JW1907	*fliA*	RNA polymerase, sigma 28 (sigma F) factor	-0.0126	0.7819
JW1912	*amyA*	Cytoplasmic alpha-amylase	0.0361	0.1818
JW1913	*yedD*	Lipoprotein	0.0395	0.1096
JW1914	*yedE*	UPF0394 family sulphur transport domain-containing inner membrane protein	0.0580	0.3690
JW1915	*yedF*	Putative tusa family sulfurtransferase	0.0297	0.4380
JW1916	*yedK*	DUF159 family protein	-0.0666	0.0144
JW1917	*yedL*	GNAT family putative N-acetyltransferase	-0.0240	0.6986
JW1918	*yedN*	CP4-44 prophage; putative disrupted hemin or colicin receptor	0.1009	0.0033
JW1920	*yedM*	Uncharacterized protein	0.0851	0.0009
JW1921	*fliE*	Flagellar basal-body component	0.0785	0.2618
JW1923	*fliG*	Flagellar motor switching and energizing component	-0.2349	0.0023
JW1924	*fliH*	Negative regulator of flii atpase activity	-0.0636	0.2452
JW1925	*fliI*	Flagellum-specific ATP synthase	0.1322	0.0001
JW1926	*fliJ*	Flagellar protein	-0.0734	0.0229
JW1932	*fliP*	Flagellar biosynthesis protein	-0.0183	0.6253
JW1934	*fliR*	Flagellar export pore protein	-0.1252	0.0157
JW1935	*rcsA*	Transcriptional regulator of colanic acid capsular biosynthesis	0.0001	0.9965
JW1936	*dsrB*	Uncharacterized protein	-0.0746	0.0150

JW1938	*yedP*	Putative mannosyl-3-phosphoglycerate phosphatase	-0.1072	0.0043
JW1940	*yodC*	Uncharacterized protein	-0.0327	0.5073
JW1941	*yedI*	DUF808 family inner membrane protein	-0.0670	0.2978
JW1942	*yedA*	Amino acid exporter for phenylalanine, threonine	-0.0563	0.0988
JW1943	*vsr*	DNA mismatch endonuclease of very short patch repair	0.1165	0.1017
JW1944	*dcm*	DNA cytosine methyltransferase	0.0057	0.8224
JW1945	*yedJ*	Putative HD superfamily phosphohydrolase	0.0219	0.6468
JW1946	*yedR*	Inner membrane protein	-0.0046	0.8924
JW1948	*yedS*	CP4-44 prophage; putative disrupted hemin or colicin receptor	-0.0021	0.9659
JW1949	*yedS*	CP4-44 prophage; putative disrupted hemin or colicin receptor	-0.0017	0.9554
JW1950	*hchA*	Glyoxalase III and Hsp31 molecular chaperone	0.0609	0.2698
JW1951	*yedV*	Putative sensory kinase in two-component regulatory system with yedw	0.0146	0.3008
JW1953	*yedX*	Hydroxyisourate hydrolase	0.0605	0.1725
JW1954	*yedY*	Membrane-anchored, periplasmic TMAO, DMSO reductase	-0.0658	0.1305
JW1955	*yedZ*	Inner membrane heme subunit for periplasmic yedyz reductase	-0.0443	0.3151
JW1956	*yodA*	Zinc and cadmium binding protein, periplasmic	0.1104	0.0010
JW1958	*yeeI*	Anti-repressor for dgsa(Mlc)	-0.0072	0.7660
JW1961	*yeeL*	CP4-44 prophage; putative disrupted hemin or colicin receptor	0.0416	0.1946
JW1962	*shiA*	Shikimate transporter	0.0023	0.9472
JW1963	*amn*	AMP nucleosidase	-0.0812	0.0083
JW1964	*yeeN*	UPF0082 family protein	0.0032	0.8716
JW1965	*yeeO*	Putative multdrug exporter, MATE family	0.0349	0.4064
JW1966	*cbl*	Ssueadcb/tauabcd operon transcriptional activator	-0.0182	0.7097
JW1967	*nac*	Nitrogen assimilation regulon transcriptional regulator; autorepressor	-0.0070	0.8794
JW1968	*erfK*	L,D-transpeptidase linking Lpp to murein	-0.0886	0.0373
JW1969	*cobT*	Nicotinate-nucleotide--dimethylbenzimidazole phosphoribosyltransferase	0.0476	0.1931
JW1970	*cobS*	Cobalamin synthase	-0.0151	0.6815
JW1971	*cobU*	Cobinamide kinase and cobinamide phosphate guanylyltransferase	-0.0139	0.6458
JW1980	*yoeE*	Uncharacterized protein	-0.0558	0.1724
JW1982	*flu*	CP4-44 prophage; antigen 43 (Ag43) phase-variable biofilm formation autotransporter	0.0024	0.9567
JW1983	*yeeR*	CP4-44 prophage; putative membrane protein	-0.0664	0.1041
JW1984	*yeeS*	CP4-44 prophage; putative DNA repair protein	-0.0012	0.9691
JW1985	*yeeT*	CP4-44 prophage; uncharacterized protein	-0.0592	0.1904
JW1986	*yeeU*	CP4-44 prophage; cytoskeleton bundling-enhancing factor A; cbta antitoxin	-0.0723	0.0465

JW1987	*yeeV*	CP4-44 prophage; toxin of the yeev-yeeu toxin-antitoxin system	-0.0896	0.0173
JW1988	*yeeW*	CP4-44 prophage; uncharacterized protein	0.0121	0.5383
JW1989	*yeeX*	UPF0265 family protein	-0.0502	0.0750
JW1990	*yeeA*	Putative transporter, FUSC family inner membrane protein	-0.0097	0.8152
JW1991	*sbmC*	DNA gyrase inhibitor	-0.0618	0.1100
JW1993	*sbcB*	Exodeoxyribonuclease I; exonuclease I	-0.0017	0.9652
JW1994	*yeeD*	Putative tusa family sulfurtransferase	-0.1018	0.0537
JW1995	*yeeE*	UPF0394 family inner membrane protein	-0.0161	0.7144
JW1998	*yeeZ*	Putative epimerase	-0.0687	0.1569
JW2000	*hisL*	His operon leader peptide	-0.0006	0.9881
JW2001	*hisG*	ATP phosphoribosyltransferase	0.0524	0.4006
JW2002	*hisD*	Bifunctional histidinal dehydrogenase/ histidinol dehydrogenase	0.1163	0.0040
JW2003	*hisC*	Histidinol-phosphate aminotransferase	0.1589	0.0252
JW2005	*hisH*	Imidazole glycerol phosphate synthase, glutamine amidotransferase subunit	0.0451	0.2574
JW2006	*hisA*	N-(5'-phospho-L-ribosyl-formimino)-5-amino-1-(5'-phosphoribosyl)-4-imidazolecarboxamide isomerase	-0.0331	0.2197
JW2007	*hisF*	Imidazole glycerol phosphate synthase, catalytic subunit with hish	0.0402	0.2759
JW2008	*hisI*	Phosphoribosyl-AMP cyclohydrolase and phosphoribosyl-ATP pyrophosphatase	-0.0783	0.0112
JW2010	*ugd*	UDP-glucose 6-dehydrogenase	0.0850	0.1171
JW2011	*gnd*	6-phosphogluconate dehydrogenase, decarboxylating	0.0195	0.4585
JW2012	*wbbL*	CP4-44 prophage; putative disrupted hemin or colicin receptor	-0.3725	0.0242
JW2016	*wbbL*	Rhamnosyltransferase wbbl	0.0329	0.3468
JW2017	*wbbK*	Lipopolysaccharide biosynthesis protein	0.0067	0.9111
JW2018	*wbbJ*	Putative lipopolysaccharide biosynthesis O-acetyl transferase	-0.0126	0.2535
JW2019	*wbbI*	D-Galf:alpha-d-Glc beta-1,6-galactofuranosyltransferase	0.0288	0.4159
JW2020	*wbbH*	O-antigen polymerase	-0.0119	0.6937
JW2023	*rfbC*	Dtdp-4-deoxyrhamnose-3,5-epimerase	-0.0114	0.7379
JW2025	*rfbD*	Dtdp-L-rhamnose synthase, NAD(P)-dependent dtdp-4-dehydrorhamnose reductase subunit	0.0347	0.6717
JW2026	*rfbB*	Dtdp-glucose 4,6 dehydratase, NAD(P)-binding	0.0018	0.9568
JW2027	*galF*	Putative regulatory subunit for galu	0.0192	0.3766
JW2028	*wcaM*	Colanic acid biosynthesis protein	0.0580	0.0870
JW2029	*wcaL*	Putative glycosyl transferase	-0.0626	0.1005
JW2030	*wcaK*	Colanic acid biosynthesis protein	0.0367	0.3361
JW2031	*wzxC*	Putative colanic acid exporter	-0.0057	0.8842
JW2032	*wcaJ*	Colanic biosynthesis UDP-glucose lipid carrier transferase	-0.0199	0.6645

JW2033	*cpsG*	Phosphomannomutase	0.2045	0.0001
JW2034	*cpsB*	Mannose-1-phosphate guanyltransferase	-0.0003	0.5767
JW2037	*fcl*	Bifunctional GDP-fucose synthetase: GDP-4-dehydro-6-deoxy-D-mannose epimerase/ GDP-4-dehydro-6-L-deoxygalactose reductase	0.0820	0.0549
JW2038	*gmd*	GDP-D-mannose dehydratase, NAD(P)-binding	0.0337	0.5044
JW2039	*wcaF*	Putative acyl transferase	0.0394	0.0180
JW2040	*wcaE*	Putative glycosyl transferase	-0.0205	0.7406
JW2041	*wcaD*	Putative colanic acid polymerase	-0.0437	0.1403
JW2042	*wcaC*	Putative glycosyl transferase	-0.0357	0.1662
JW2043	*wcaB*	Putative acyl transferase	0.0979	0.0700
JW2045	*wzc*	Colanic acid production tyrosine-protein kinase; autokinase; Ugd phosphorylase	-0.0387	0.3830
JW2046	*wzb*	Colanic acid production protein-tyrosine-phosphatase; Wzc-P dephosphorylase	-0.0424	0.3735
JW2047	*wza*	Colanic acid export protein; outer membrane auxillary lipoprotein	-0.0183	0.7798
JW2049	*asmA*	Suppressor of ompf assembly mutants; putative outer membrane protein assembly factor; inner membrane-anchored periplasmic protein	-0.0108	0.8049
JW2050	*dcd*	Deoxycytidine triphosphate deaminase; dctp deaminase	-0.0604	0.1526
JW2051	*udk*	Uridine-cytidine kinase	-0.0345	0.3900
JW2052	*yegE*	Putative diguanylate cyclase	0.0026	0.9186
JW2053	*alkA*	3-methyl-adenine DNA glycosylase II	-0.0394	0.3240
JW2054	*yegD*	Hsp70 chaperone family protein	0.0892	0.0821
JW2055	*yegI*	Protein kinase-related putative non-specific DNA-binding protein	-0.0003	0.9906
JW2056	*yegJ*	DUF2314 family protein	-0.1285	0.0016
JW2057	*yegK*	Ser/thr phosphatase-related protein	-0.0723	0.0573
JW2058	*yegL*	VMA domain protein	0.0191	0.5571
JW2060	*mdtB*	Multidrug efflux system, subunit B	-0.0141	0.7252
JW2061	*mdtC*	Multidrug efflux system, subunit C	0.0342	0.5168
JW2062	*mdtD*	Putative citrate/iron-citrate/zinc-citrate efflux transporter	-0.0710	0.2059
JW2063	*baeS*	Sensory histidine kinase in two-component regulatory system with baer	-0.0798	0.1587
JW2064	*baeR*	Response regulator in two-component regulatory system with baes	-0.0962	0.0208
JW2066	*yegQ*	Putative peptidase	0.0486	0.2375
JW2067	*ogrK*	Orphan Ogr protein, positive regulator of P2 growth	0.0668	0.0197
JW2070	*yegS*	Phosphatidylglycerol kinase, metal-dependent	0.0024	0.9387
JW2074	*gatR*	CP4-44 prophage; putative disrupted hemin or colicin receptor	-0.0209	0.6527
JW2075	*gatD*	Galactitol-1-phosphate dehydrogenase, Zn-dependent and NAD(P)-binding	-0.0087	0.7983

JW2076	*gatC*	Pseudogene, galactitol-specific enzyme IIC component of PTS	0.0300	0.3643
JW2077	*gatB*	Galactitol-specific enzyme IIB component of PTS	0.0219	0.3965
JW2082	*gatZ*	D-tagatose 1,6-bisphosphate aldolase 2, subunit	0.0130	0.6951
JW2085	*yegT*	Nucleoside transporter, low affinity	-0.0382	0.5287
JW2086	*yegU*	ADP-ribosylglycohydrolase family protein	-0.0140	0.7009
JW2087	*yegV*	Putative kinase	0.0150	0.4858
JW2088	*yegW*	Putative DNA-binding transcriptional regulator	-0.0250	0.5361
JW2090	*thiD*	Hydroxy-methylpyrimidine kinase and hydroxy-phosphomethylpyrimidine kinase	0.0906	0.0088
JW2091	*thiM*	Hydroxyethylthiazole kinase	-0.0258	0.3268
JW2092	*yohL*	Transcriptional repressor of rcna	-0.0244	0.4525
JW2095	*yehA*	Putative fimbrial-like adhesin protein	-0.0471	0.2481
JW2096	*yehB*	Putative outer membrane protein	0.0337	0.4039
JW2097	*yehC*	Putative periplasmic pilin chaperone	0.0265	0.3615
JW2098	*yehD*	Putative fimbrial-like adhesin protein	0.0370	0.0219
JW2099	*yehE*	DUF2574 family protein	-0.0219	0.3200
JW2100	*mrp*	Antiporter inner membrane protein	-0.0031	0.9449
JW2102	*molR*	CP4-44 prophage; putative disrupted hemin or colicin receptor	0.0487	0.1458
JW2105	*yehI*	Uncharacterized protein	0.0675	0.0099
JW2106	*yehK*	Uncharacterized protein	0.0061	0.7993
JW2108	*yehM*	Uncharacterized protein	0.0225	0.4507
JW2110	*yehQ*	Pseudogene	-0.0150	0.5213
JW2112	*yehS*	DUF1456 family protein	-0.0581	0.1319
JW2115	*mlrA*	Transcriptional activator of csgd and csgba	-0.0411	0.2893
JW2116	*yehW*	Putative ABC transporter permease	-0.0506	0.0937
JW2117	*yehX*	Putative ABC transporter atpase	0.0077	0.8320
JW2119	*yehZ*	Putative ABC transporter periplasmic binding protein	0.0706	0.0019
JW2124	*yohD*	Deda family inner membrane protein	-0.0119	0.6595
JW2125	*yohF*	Putative oxidoreductase	0.0362	0.5235
JW2128	*dusC*	Trna-dihydrouridine synthase C	0.0352	0.4194
JW2129	*yohJ*	UPF0299 family inner membrane protein	-0.0977	0.0684
JW2130	*yohK*	Lrgb family inner membrane protein	0.0372	0.3335
JW2131	*cdd*	Cytidine/deoxycytidine deaminase	0.0198	0.6295
JW2132	*sanA*	DUF218 superfamily vancomycin high temperature exclusion protein	0.1193	0.0004
JW2133	*yeiT*	Dihydropyrimidine dehydrogenase, NADH-dependent, subunit N	0.0342	0.1042
JW2134	*yeiA*	Dihydropyrimidine dehydrogenase, NADH-dependent, subunit C	0.0607	0.0531
JW2135	*mglC*	Methyl-galactoside transporter subunit	0.0044	0.8796
JW2136	*mglA*	Methyl-galactoside ABC transporter atpase	-0.0500	0.2400
JW2137	*mglB*	Methyl-galactoside transporter subunit	0.0032	0.9229

JW2139	*yeiB*	DUF418 family putative inner membrane protein	-0.0670	0.0586
JW2141	*yeiG*	S-formylglutathione hydrolase	-0.0086	0.7509
JW2143	*lysP*	Lysine transporter	-0.0567	0.0790
JW2144	*yeiE*	Putative DNA-binding transcriptional regulator	-0.0088	0.8074
JW2145	*yeiH*	UPF0324 family inner membrane protein	0.0243	0.2679
JW2146	*nfo*	Endonuclease IV with intrinsic 3'-5' exonuclease activity	0.0580	0.0461
JW2147	*yeiI*	Putative kinase	-0.0193	0.7850
JW2148	*yeiJ*	Nucleoside permease	0.0530	0.2976
JW2149	*rihB*	Ribonucleoside hydrolase 2	0.0555	0.4261
JW2150	*yeiL*	Nitrogen starvation viability factor; putative cola sensitivity effector; putative Crp-Fnr family transcriptional regulator	0.0047	0.8781
JW2151	*yeiM*	Putative nucleoside transporter	-0.0555	0.0979
JW2152	*yeiN*	Pseudouridine 5'-phosphate glycosidase	0.0587	0.0436
JW2153	*yeiC*	Pseudouridine kinase	0.0226	0.6854
JW2154	*fruA*	Fused fructose-specific PTS enzymes: iibcomponent/IIC components	-0.0335	0.2688
JW2155	*fruK*	Fructose-1-phosphate kinase	0.0060	0.8802
JW2156	*fruB*	Fused fructose-specific PTS enzymes: IIA component/hpr component	0.0146	0.7427
JW2157	*setB*	Lactose/glucose efflux system	-0.1023	0.0269
JW2160	*yeiQ*	Putative NAD-dependent D-mannonate oxidoreductase	-0.0239	0.5261
JW2161	*yeiR*	Zn-stimulated gtpase involved in zinc homeostasis; mutants are cadmium and EDTA sensitive; Zn(2+) binding protein	-0.1363	0.0097
JW2162	*yeiU*	Lipid A 1-diphosphate synthase; undecaprenyl pyrophosphate:lipid A 1-phosphate phosphotransferase	0.0185	0.6284
JW2164	*rtn*	Resistance protein for phages lambda and N4, putative membrane-anchored cyclic-di-GMP phosphodiesterase	0.0479	0.2868
JW2165	*yejA*	Microcin C ABC transporter periplasmic binding protein	0.0633	0.1277
JW2166	*yejB*	Microcin C ABC transporter permease	-0.0042	0.8963
JW2167	*yejE*	Microcin C ABC transporter permease	-0.0426	0.1904
JW2168	*yejF*	Microcin C ABC transporter atpase	0.1291	0.0819
JW2169	*yejG*	Uncharacterized protein	-0.0595	0.1915
JW2171	*rsuA*	16S rrna pseudouridine(516) synthase	-0.0028	0.8935
JW2172	*yejH*	Putative ATP-dependent DNA or RNA helicase	0.0099	0.8167
JW2173	*rplY*	50S ribosomal subunit protein L25	0.0541	0.1488
JW2174	*yejK*	Nucleoid-associated protein yejk	-0.0565	0.1021
JW2175	*yejL*	UPF0352 family protein	-0.0321	0.2603
JW2181	*narP*	Response regulator in two-component regulatory system with narq	-0.0688	0.1245
JW2182	*ccmH*	Heme lyase, ccmh subunit	0.1168	0.0615

JW2184	*ccmF*	Heme lyase, ccmf subunit	0.1294	0.0031
JW2185	*ccmE*	Periplasmic heme chaperone	-0.0506	0.1968
JW2186	*ccmD*	Heme exporter protein D	0.0242	0.3143
JW2187	*ccmC*	Heme exporter protein C	-0.0867	0.0098
JW2188	*ccmB*	Heme export ABC transporter permease	-0.0027	0.9235
JW2190	*napC*	Quinol dehydrogenase, electron source for napab	-0.0246	0.2464
JW2192	*napH*	Ferredoxin-type protein	-0.0408	0.1862
JW2193	*napG*	Ferredoxin-type protein	-0.0070	0.8470
JW2194	*napA*	Nitrate reductase, periplasmic, large subunit	-0.0071	0.8267
JW2195	*napD*	Assembly protein for periplasmic nitrate reductase	0.0292	0.4540
JW2196	*napF*	Ferredoxin-type protein, role in electron transfer to periplasmic nitrate reductase napa	-0.0973	0.0339
JW2197	*eco*	Ecotin, a serine protease inhibitor	-0.0130	0.6974
JW2198	*mqo*	Malate dehydrogenase, FAD/NAD(P)-binding domain	-0.0396	0.5094
JW2199	*yojI*	Microcin J25 efflux ABC transporter permease/atpase	-0.0312	0.3485
JW2200	*alkB*	Oxidative demethylase of N1-methyladenine or N3-methylcytosine DNA lesions	-0.0339	0.3900
JW2203	*ompC*	Outer membrane porin protein C	0.2658	0.0000
JW2204	*rcsD*	Phosphotransfer intermediate protein in two-component regulatory system with rcsbc	0.0404	0.2606
JW2205	*rcsB*	Response regulator in two-component regulatory system with rcsc and yojn	0.0007	0.9873
JW2213	*atoS*	Sensory histidine kinase in two-component regulatory system with atoc	-0.0100	0.8805
JW2214	*atoC*	Fused response regulator of ato operon, in two-component system with atos: response regulator/sigma54 interaction protein	-0.0725	0.1221
JW2215	*atoD*	Acetyl-coa:acetoacetyl-coa transferase, alpha subunit	0.1274	0.0015
JW2216	*atoA*	Acetyl-coa:acetoacetyl-coa transferase, beta subunit	0.2461	0.0000
JW2217	*atoE*	Short chain fatty acid transporter	-0.0643	0.2363
JW2218	*atoB*	Acetyl-coa acetyltransferase	-0.0078	0.7334
JW2219	*yfaP*	DUF2135 family protein, putative host defense protein	0.0005	0.0005
JW2220	*yfaQ*	Tandem DUF2300 domain protein, putative host defense protein	-0.1597	0.0011
JW2221	*yfaS*	CP4-44 prophage; putative disrupted hemin or colicin receptor	-0.0612	0.3596
JW2222	*yfaS*	CP4-44 prophage; putative disrupted hemin or colicin receptor	-0.0106	0.8044
JW2223	*yfaT*	DUF1175 family protein, putative host defense protein	0.0187	0.5324
JW2224	*yfaA*	DUF2138 family protein, putative host defense protein	0.0185	0.5870

JW2226	*ubiG*	Bifunctional 3-demethylubiquinone-9 3-methyltransferase/ 2-octaprenyl-6-hydroxy phenol methylase	-0.2647	0.0092
JW2230	*yfaE*	Ferredoxin involved with ribonucleotide reductase diferric-tyrosyl radical (Y*) cofactor maintenance	0.0508	0.2781
JW2231	*inaA*	Acid-inducible Kdo/waap family putative kinase	-0.0365	0.3612
JW2232	*yfaH*	Pseudogene	-0.0477	0.0451
JW2233	*glpQ*	Periplasmic glycerophosphodiester phosphodiesterase	0.0075	0.8980
JW2234	*glpT*	Sn-glycerol-3-phosphate transporter	-0.0302	0.1406
JW2235	*glpA*	Anaerobic sn-glycerol-3-phosphate dehydrogenase, large FAD/NAD(P)-binding subunit	-0.0011	0.9753
JW2236	*glpB*	Anaerobic sn-glycerol-3-phosphate dehydrogenase membrane anchor subunit	0.0042	0.8150
JW2237	*glpC*	Anaerobic sn-glycerol-3-phosphate dehydrogenase, C subunit, 4Fe-4S iron-sulfur cluster	0.0007	0.0312
JW2238	*yfaD*	Transposase_31 family protein	-0.0240	0.6103
JW2239	*yfaU*	2-keto-3-deoxy-L-rhamnonate aldolase	0.0502	0.1384
JW2240	*yfaV*	Putative L-rhamnonate transporter	-0.0390	0.2826
JW2241	*yfaW*	L-rhamnonate dehydratase	-0.1182	0.0488
JW2242	*yfaX*	Putative DNA-binding transcriptional regulator for the rhm operon	-0.0262	0.2378
JW2243	*yfaY*	Inactive pncc family protein	0.0288	0.6315
JW2245	*yfaO*	Nucleoside triphosphatase	0.0110	0.5950
JW2246	*ais*	Putative LPS core heptose(II)-phosphate phosphatase	-0.0133	0.7101
JW2248	*yfbF*	Undecaprenyl phosphate-L-Ara4FN transferase	-0.0142	0.7757
JW2249	*yfbG*	Fused UDP-L-Ara4N formyltransferase/UDP-glca C-4'-decarboxylase	0.0369	0.4987
JW2250	*yfbH*	Undecaprenyl phosphate-alpha-L-ara4fn deformylase	-0.0625	0.0833
JW2251	*arnT*	4-amino-4-deoxy-L-arabinose transferase	-0.0449	0.2763
JW2252	*yfbW*	Undecaprenyl phosphate-alpha-L-ara4n exporter; flippase arnef subunit	-0.0226	0.6162
JW2254	*pmrD*	Inactive two-component system connector protein	0.1058	0.0420
JW2255	*menE*	O-succinylbenzoate-coa ligase	0.0601	0.2071
JW2256	*menC*	O-succinylbenzoyl-coa synthase	0.0434	0.4222
JW2257	*menB*	Dihydroxynaphthoic acid synthetase	0.0010	0.9818
JW2258	*yfbB*	2-succinyl-6-hydroxy-2,4-cyclohexadiene-1-carboxylate synthase	0.0893	0.0171
JW2260	*menF*	Isochorismate synthase 2	0.0258	0.3723
JW2261	*elaB*	Putative membrane-anchored DUF883 family ribosome-binding protein	-0.1328	0.0011
JW2263	*elaC*	Rnase BN, trna processing enzyme	-0.0700	0.0423
JW2265	*yfbK*	Von Willebrand factor domain putative lipoprotein	0.0645	0.0609

JW2267	*yfbM*	DUF1877 family protein	0.0216	0.6338
JW2269	*yfbO*	Uncharacterized protein	-0.0134	0.7134
JW2270	*yfbP*	TPR-like repeats-containing protein	-0.0282	0.5340
JW2271	*nuoN*	NADH:ubiquinone oxidoreductase, membrane subunit N	0.0424	0.2184
JW2272	*nuoM*	NADH:ubiquinone oxidoreductase, membrane subunit M	0.0027	0.9197
JW2273	*nuoL*	NADH:ubiquinone oxidoreductase, membrane subunit L	-0.0117	0.8094
JW2274	*nuoK*	NADH:ubiquinone oxidoreductase, membrane subunit K	-0.2468	0.0005
JW2275	*nuoJ*	NADH:ubiquinone oxidoreductase, membrane subunit J	0.0363	0.2882
JW2276	*nuoI*	NADH:ubiquinone oxidoreductase, chain I	0.1234	0.0047
JW2277	*nuoH*	NADH:ubiquinone oxidoreductase, membrane subunit H	-0.0142	0.6402
JW2278	*nuoG*	NADH:ubiquinone oxidoreductase, chain G	-0.0279	0.4603
JW2279	*nuoF*	NADH:ubiquinone oxidoreductase, chain F	0.0696	0.0857
JW2280	*nuoE*	NADH:ubiquinone oxidoreductase, chain E	0.0817	0.0601
JW2283	*nuoA*	NADH:ubiquinone oxidoreductase, membrane subunit A	-0.0096	0.7643
JW2284	*lrhA*	Transcriptional repressor of flagellar, motility and chemotaxis genes	-0.0019	0.9477
JW2287	*yfbQ*	Glutamate-pyruvate aminotransferase; glutamic-pyruvic transaminase (GPT); alanine transaminase	0.0217	0.5036
JW2288	*yfbR*	5'-nucleotidase	0.0445	0.0631
JW2289	*yfbS*	Putative transporter	-0.0863	0.0119
JW2291	*yfbU*	UPF0304 family protein	0.0428	0.2498
JW2292	*yfbV*	UPF0208 family inner membrane protein	0.0640	0.1380
JW2293	*ackA*	Acetate kinase A and propionate kinase 2	0.1550	0.0567
JW2295	*yfcC*	Putative inner membrane transporter; C4-dicarboxylate anaerobic carrier family protein	-0.0431	0.0118
JW2296	*yfcD*	Putative NUDIX hydrolase	0.0649	0.0716
JW2298	*yfcF*	Glutathione S-transferase	-0.0442	0.1852
JW2299	*yfcG*	GSH-dependent disulfide bond oxidoreductase	0.0008	0.9795
JW2300	*folX*	D-erythro-7,8-dihydroneopterin triphosphate 2'-epimerase and dihydroneopterin aldolase	-0.0525	0.0674
JW2301	*yfcH*	Putative NAD-dependent nucleotide-sugar epimerase	0.0103	0.8108
JW2303	*hisP*	Histidine ABC transporter atpase	0.0013	0.9697
JW2304	*hisM*	Histidine ABC transporter permease	-0.0377	0.2304
JW2305	*hisQ*	Histidine ABC transporter permease	0.0056	0.7696
JW2306	*hisJ*	Histidine ABC transporter periplasmic binding protein	0.0872	0.0196
JW2307	*argT*	Lysine/arginine/ornithine transporter subunit	0.0314	0.5674
JW2308	*ubiX*	3-octaprenyl-4-hydroxybenzoate carboxy-lyase	-0.0372	0.3006
JW2309	*purF*	Amidophosphoribosyltransferase	-0.0369	0.4296

JW2310	*cvpA*	Colicin V production protein	0.0754	0.1130
JW2314	*dedA*	Deda family inner membrane protein	0.0379	0.5486
JW2315	*truA*	Trna pseudouridine(38-40) synthase	0.0982	0.0394
JW2316	*usg*	Putative semialdehyde dehydrogenase	0.0278	0.4513
JW2317	*pdxB*	Erythronate-4-phosphate dehydrogenase	-0.0471	0.2800
JW2318	*flk*	Putative flagella assembly protein	-0.0599	0.1300
JW2319	*yfcJ*	Putative arabinose efflux transporter	-0.0679	0.0447
JW2322	*yfcL*	Uncharacterized protein	-0.0333	0.4099
JW2324	*yfcA*	Taue/TSUP family inner membrane protein	-0.0027	0.9404
JW2325	*mepA*	Murein DD-endopeptidase	0.0075	0.8603
JW2326	*aroC*	Chorismate synthase	0.0602	0.7458
JW2328	*yfcN*	Putative DNA endonuclease	0.0198	0.6211
JW2329	*yfcO*	DUF2544 family putative outer membrane protein	0.0787	0.1179
JW2330	*yfcP*	Putative fimbrial-like adhesin protein	-0.0172	0.7756
JW2331	*yfcQ*	Putative fimbrial-like adhesin protein	0.0091	0.8877
JW2332	*yfcR*	Putative fimbrial-like adhesin protein	-0.0903	0.2022
JW2333	*yfcS*	Putative periplasmic pilin chaperone	-0.0704	0.2501
JW2335	*yfcU*	CP4-44 prophage; putative disrupted hemin or colicin receptor	0.0254	0.3645
JW2337	*sixA*	Phosphohistidine phosphatase	0.1489	0.0255
JW2338	*yfcX*	Enoyl-coa hydratase/epimerase and isomerase/3-hydroxyacyl-coa dehydrogenase	-0.0039	0.9186
JW2339	*yfcY*	Beta-ketoacyl-coa thiolase, anaerobic, subunit	0.0117	0.8037
JW2340	*yfcZ*	UPF0381 family protein	0.0058	0.9092
JW2341	*fadL*	Long-chain fatty acid outer membrane transporter	-0.0102	0.8148
JW2343	*vacJ*	ABC transporter maintaining OM lipid asymmetry, OM lipoprotein component	0.0176	0.5937
JW2344	*yfdC*	Inner membrane protein	-0.0117	0.7675
JW2345	*intS*	CPS-53 (kple1) prophage; putative prophage CPS-53 integrase	0.0514	0.2075
JW2346	*yfdG*	CPS-53 (kple1) prophage; bactoprenol-linked glucose translocase/flippase	0.0194	0.6073
JW2347	*yfdH*	CPS-53 (kple1) prophage; bactoprenol glucosyl transferase	-0.0156	0.6605
JW2350	*yfdK*	CPS-53 (kple1) prophage; conserved protein	-0.0289	0.5576
JW2352	*yfdM*	CPS-53 (kple1) prophage; putative methyltransferase	0.0180	0.6359
JW2355	*yfdO*	Pseudogene, CPS-53 (kple1) prophage; bacteriophage replication protein O family	0.0606	0.0454
JW2356	*yfdP*	CPS-53 (kple1) prophage; uncharacterized protein	0.0704	0.0020
JW2357	*yfdQ*	CPS-53 (kple1) prophage; uncharacterized protein	-0.0173	0.7052
JW2358	*yfdR*	CPS-53 (kple1) prophage; conserved protein	0.0245	0.5290
JW2359	*yfdS*	CPS-53 (kple1) prophage; uncharacterized protein	-0.0007	0.9663
JW2362	*dsdX*	D-serine transporter	-0.0548	0.3020
JW2363	*dsdA*	D-serine dehydratase	-0.1358	0.0221
JW2364	*emrY*	Putative multidrug efflux system	0.0413	0.4121

JW2365	*emrK*	Multidrug resistance efflux pump membrane fusion protein	-0.0019	0.9682
JW2366	*evgA*	Response regulator in two-component regulatory system with evgs	-0.1664	0.0003
JW2367	*evgS*	Hybrid sensory histidine kinase in two-component regulatory system with evga	0.0758	0.0213
JW2368	*yfdE*	Acetyl-coa:oxalate coa-transferase	-0.0429	0.2476
JW2369	*yfdV*	Putative transporter	0.1601	0.0124
JW2370	*oxc*	Oxalyl coa decarboxylase, thdp-dependent	0.5186	0.0004
JW2371	*frc*	Formyl-coa transferase, NAD(P)-binding	-0.0236	0.6319
JW2372	*yfdX*	Uncharacterized protein	0.0448	0.0357
JW2373	*ypdI*	Putative lipoprotein involved in colanic acid biosynthesis	0.0121	0.7596
JW2374	*yfdY*	DUF2545 family putative inner membrane protein	-0.0543	0.0853
JW2375	*ddg*	Palmitoleoyl-acyl carrier protein (ACP)-dependent acyltransferase	0.1176	0.0158
JW2376	*yfdZ*	Glutamate-pyruvate aminotransferase; glutamic-pyruvic transaminase (GPT); alanine transaminase	0.0111	0.7891
JW2378	*ypdB*	Response regulator activating yhjx; pyruvate-responsive ypdab two-component system	0.0310	0.6281
JW2379	*ypdC*	Putative DNA-binding protein	0.1044	0.1785
JW2381	*ypdE*	Aminopeptidase	-0.0367	0.4365
JW2382	*ypdF*	Xaa-Pro aminopeptidase	-0.0075	0.8239
JW2383	*ypdG*	Putative enzyme IIC component of PTS	-0.0132	0.6082
JW2385	*glk*	Glucokinase	-0.0114	0.7158
JW2386	*yfeO*	Putative ion channel protein	0.0168	0.5333
JW2387	*ypeC*	DUF2502 family putative periplasmic protein	-0.0678	0.0361
JW2388	*mntH*	Manganese/divalent cation transporter	-0.0936	0.1565
JW2389	*nupC*	Nucleoside (except guanosine) transporter	0.0702	0.1122
JW2393	*yfeC*	DUF1323 family putative DNA-binding protein	0.0044	0.8830
JW2394	*yfeD*	DUF1323 family putative DNA-binding protein	-0.0214	0.6095
JW2397	*xapB*	Xanthosine transporter	-0.0187	0.6757
JW2398	*xapA*	Purine nucleoside phosphorylase 2; nicotinamide 1-beta-D-riboside synthase	0.0370	0.3119
JW2399	*yfeN*	Putative outer membrane protein	-0.0242	0.4228
JW2400	*yfeR*	Transcriptional regulator of yefh	0.0848	0.0059
JW2406	*cysZ*	Sulfate transporter, sulfite inhibited	-0.0204	0.5065
JW2407	*cysK*	Cysteine synthase A, O-acetylserine sulfhydrolase A subunit	-0.0187	0.7887
JW2408	*ptsH*	Phosphocarrier protein hpr	-0.0361	0.4920
JW2409	*ptsI*	PEP-protein phosphotransferase of PTS system (enzyme I)	0.0621	0.0325
JW2410	*crr*	Glucose-specific enzyme IIA component of PTS	-0.0206	0.5440
JW2412	*yfeK*	Uncharacterized protein	-0.1309	0.0204
JW2413	*yfeS*	WGR domain protein	-0.0982	0.0587

JW2414	*cysM*	Cysteine synthase B (O-acetylserine sulfhydrolase B)	-0.1349	0.0016
JW2415	*cysA*	Sulfate/thiosulfate transporter subunit	0.1148	0.1438
JW2416	*cysW*	Sulfate/thiosulfate ABC transporter permease	0.0782	0.0452
JW2417	*cysU*	Sulfate/thiosulfate ABC transporter permease	0.3619	0.0001
JW2418	*cysP*	Thiosulfate-binding protein	0.1327	0.0009
JW2420	*yfeT*	Repressor for murpq, murnac 6-P inducible	-0.2366	0.1325
JW2421	*yfeU*	N-acetylmuramic acid 6-phosphate (murnac-6-P) etherase	0.0646	0.0837
JW2422	*murP*	N-acetylmuramic acid permease, EIIBC component, PTS system	-0.0511	0.2360
JW2424	*yfeX*	Porphyrinogen oxidase, cytoplasmic	0.0418	0.2525
JW2425	*yfeY*	Rpoe-regulated lipoprotein	-0.0128	0.5821
JW2426	*yfeZ*	Inner membrane protein	-0.0131	0.7615
JW2427	*ypeA*	GNAT family putative N-acetyltransferase	-0.0462	0.3861
JW2429	*hemF*	Oxygen-dependent coproporphyrinogen-III oxidase	-0.0126	0.7434
JW2430	*yfeG*	Eut operon transcriptional activator, arac family	0.0070	0.8138
JW2431	*yffI*	Putative ethanol utilization carboxysome structural protein	0.0260	0.4564
JW2432	*eutL*	Putative ethanol utilization carboxysome structural protein	-0.0079	0.6729
JW2433	*eutC*	Ethanolamine ammonia-lyase, small subunit (light chain)	-0.0113	0.7513
JW2434	*eutB*	Ethanolamine ammonia-lyase, large subunit, heavy chain	-0.0186	0.7199
JW2435	*eutA*	Reactivating factor for ethanolamine ammonia lyase	0.0336	0.4470
JW2436	*eutH*	Ethanolamine transporter	0.1235	0.0267
JW2437	*eutG*	Ethanol dehydrogenase involved in ethanolamine utilization; aldehyde reductase	0.0017	0.9558
JW2438	*eutJ*	Ethanolamine utilization protein, HSP70/actin superfamily protein	0.1915	0.0000
JW2439	*eutE*	Aldehyde oxidoreductase, ethanolamine utilization protein	0.0067	0.8603
JW2440	*cchB*	Ethanolamine catabolic microcompartment shell protein	-0.0173	0.7868
JW2441	*cchA*	Ethanolamine utilization protein, putative carboxysome structural protein	-0.0019	0.9538
JW2442	*eutI*	Phosphate acetyltransferase	0.0458	0.1561
JW2443	*eutT*	Cobalamin adenosyltransferase involved in ethanolamine utilization	0.0684	0.2399
JW2444	*eutQ*	Rmlc-like cupin domain protein	0.0200	0.6135
JW2445	*eutP*	Putative P-loop ntpase ethanolamine utilization protein	-0.0883	0.0321
JW2446	*ypfE*	Putative ethanol utilization carboxysome structural protein	0.0206	0.3951
JW2447	*maeB*	Malic enzyme: putative oxidoreductase/phosphotransacetylase	0.1338	0.0664
JW2448	*talA*	Transaldolase A	-0.0055	0.8633

JW2449	*tktB*	Transketolase 2, thiamine triphosphate-binding	0.0332	0.3452
JW2450	*ypfG*	DUF1176 family protein	0.0181	0.7001
JW2451	*yffH*	GDP-mannose pyrophosphatase	0.0013	0.9604
JW2452	*aegA*	Putative oxidoreductase, fes binding subunit/NAD/FAD-binding subunit	-0.0470	0.1026
JW2453	*narQ*	Sensory histidine kinase in two-component regulatory system with narp	-0.0525	0.0839
JW2454	*acrD*	Aminoglycoside/multidrug efflux system	0.0491	0.4576
JW2455	*yffB*	Putative arsc family reductase	-0.0381	0.3772
JW2457	*ypfN*	Putative membrane protein, UPF0370 family	0.0053	0.9040
JW2459	*ypfI*	Elongator methionine trna (ac4c34) acetyltransferase	-0.1053	0.0123
JW2460	*ypfJ*	Putative neutral zinc metallopeptidase	0.0478	0.1879
JW2461	*purC*	Phosphoribosylaminoimidazole-succinocarboxamide synthetase	0.0324	0.0025
JW2462	*nlpB*	Bamabcde complex OM biogenesis lipoprotein	-0.0273	0.5722
JW2464	*gcvR*	Transcriptional repressor, regulatory protein accessory to gcva	0.0970	0.0379
JW2466	*hyfA*	Hydrogenase 4, 4Fe-4S subunit	0.0249	0.5790
JW2467	*hyfB*	Hydrogenase 4, membrane subunit	-0.0030	0.9517
JW2468	*hyfC*	Hydrogenase 4, membrane subunit	-0.0098	0.8053
JW2469	*hyfD*	Hydrogenase 4, membrane subunit	-0.0532	0.1147
JW2470	*hyfE*	Hydrogenase 4, membrane subunit	0.1170	0.0010
JW2471	*hyfF*	Hydrogenase 4, membrane subunit	-0.0069	0.8161
JW2472	*hyfG*	Hydrogenase 4, subunit	0.0139	0.7451
JW2473	*hyfH*	Hydrogenase 4, Fe-S subunit	0.0180	0.5318
JW2475	*hyfJ*	Putative processing element hydrogenase 4	-0.0450	0.4183
JW2476	*hyfR*	Hydrogenase-4 transcriptional activator	0.0785	0.2247
JW2477	*focB*	Putative formate transporter	0.1031	0.0643
JW2478	*yfgO*	Putative UPF0118 family inner membrane permease	-0.0038	0.9215
JW2479	*yfgC*	OM protein maintenance and assembly metalloprotease and chaperone, periplasmic	-0.0256	0.2593
JW2480	*yfgD*	Putative oxidoreductase	-0.0023	0.9450
JW2482	*uraA*	Uracil permease	0.0279	0.3663
JW2483	*upp*	Uracil phosphoribosyltransferase	-0.0130	0.6525
JW2484	*purM*	Phosphoribosylaminoimidazole synthetase	0.0068	0.8360
JW2485	*purN*	Phosphoribosylglycinamide formyltransferase 1	-0.0378	0.1875
JW2486	*ppk*	Polyphosphate kinase, component of RNA degradosome	0.0683	0.2897
JW2487	*ppx*	Exopolyphosphatase	-0.0676	0.1970
JW2488	*yfgF*	Cyclic-di-GMP phosphodiesterase, anaerobic	-0.0904	0.1128
JW2490	*yfgI*	Nalidixic acid resistance protein, putative periplasmic protein	0.0329	0.2107
JW2491	*guaA*	GMP synthetase (glutamine aminotransferase)	-0.0132	0.0606
JW2493	*xseA*	Exonuclease VII, large subunit	0.0037	0.8947

JW2496	*yfgL*	Bamabcde complex OM biogenesis lipoprotein	0.0074	0.8131
JW2497	*yfgM*	Ancillary secyeg translocon subunit; putative anti-rcsb factor	-0.1036	0.0151
JW2500	*yfgA*	Mreb assembly cytoskeletal protein	0.1294	0.0040
JW2501	*yfgB*	Dual specificity 23S rrna m(2)A2503, trna m(2)A37 methyltransferase, SAM-dependent	-0.0212	0.3603
JW2502	*ndk*	Multifunctional nucleoside diphosphate kinase and apyrimidinic endonuclease and 3'-phosphodiesterase	0.0318	0.2765
JW2503	*pbpC*	Penicillin-insensitive murein repair transglycosylase; inactive transpeptidase domain protein	-0.0003	0.9927
JW2504	*yfhM*	Bacterial alpha2-macroglobulin colonization factor ECAM; anti-host protease defense factor; periplasmic inner membrane-anchored lipoprotein	0.0401	0.3254
JW2507	*pepB*	Aminopeptidase B	0.0408	0.5576
JW2508	*yfhJ*	Fe(2+) donor and activity modulator for cysteine desulfurase	0.0992	0.2066
JW2509	*fdx*	[2Fe-2S] ferredoxin	-0.1676	0.0028
JW2510	*hscA*	Dnak-like molecular chaperone specific for iscu	0.0009	0.9811
JW2511	*hscB*	Hsca co-chaperone, J domain-containing protein Hsc56; iscu-specific chaperone hscab	-0.0126	0.6180
JW2512	*iscA*	Fes cluster assembly protein	0.1163	0.0006
JW2513	*iscU*	Iron-sulfur cluster assembly scaffold protein	-0.0066	0.8983
JW2514	*iscS*	Cysteine desulfurase (trna sulfurtransferase), PLP-dependent	0.0000	0.9993
JW2515	*iscR*	Isc operon transcriptional repressor; suf operon transcriptional activator; oxidative stress- and iron starvation-inducible; autorepressor	-0.0779	0.0980
JW2518	*yfhR*	Putative S9 family prolyl oligopeptidase	0.0226	0.5000
JW2520	*hcaT*	Putative 3-phenylpropionic transporter	0.0172	0.7007
JW2521	*hcaR*	Hca operon transcriptional regulator	-0.0203	0.7028
JW2523	*hcaF*	3-phenylpropionate dioxygenase, small (beta) subunit	0.1350	0.0017
JW2524	*hcaC*	3-phenylpropionate dioxygenase, ferredoxin subunit	0.1019	0.0354
JW2525	*hcaB*	2,3-dihydroxy-2,3-dihydrophenylpropionate dehydrogenase	0.0583	0.0071
JW2526	*hcaD*	Phenylpropionate dioxygenase, ferredoxin reductase subunit	0.0341	0.2850
JW2527	*yphA*	Doxx family inner membrane protein	0.0515	0.0676
JW2528	*yphB*	Mutarotase superfamily protein, yphb family	0.0232	0.4330
JW2530	*yphD*	Putative sugar ABC transporter permease	-0.0584	0.2279
JW2531	*yphE*	Putative sugar ABC transporter atpase	0.0277	0.5579
JW2532	*yphF*	ABC transporter periplasmic-binding protein yphf	0.0434	0.3434
JW2535	*glyA*	Serine hydroxymethyltransferase	-0.0831	0.2113

JW2536	*hmp*	Fused nitric oxide dioxygenase/dihydropteridine reductase 2	0.1290	0.0042
JW2537	*glnB*	Regulatory protein P-II for glutamine synthetase	0.0884	0.3707
JW2538	*yfhA*	Response regulator regulating glmy srna in two-component system with sensor protein glrk	0.0426	0.1597
JW2539	*yfhG*	Putative outer membrane protein modulating the qseef response	0.0622	0.1388
JW2541	*purL*	Phosphoribosylformyl-glycineamide synthetase	-0.0618	0.1779
JW2542	*yfhD*	Membrane-bound lytic transglycosylase F, murein hydrolase	-0.0288	0.6128
JW2545	*yfhH*	Putative DNA-binding transcriptional regulator	-0.0412	0.2593
JW2546	*yfhL*	Putative 4Fe-4S cluster-containing protein	-0.0329	0.5653
JW2548	*pdxJ*	Pyridoxine 5'-phosphate synthase	0.0041	0.9272
JW2549	*recO*	Gap repair protein	-0.0345	0.4591
JW2553	*lepA*	Back-translocating elongation factor EF4, gtpase	0.0447	0.0449
JW2554	*rseC*	Soxr iron-sulfur cluster reduction factor component	0.0196	0.4769
JW2555	*rseB*	Anti-sigma E factor, binds rsea	0.0481	0.1343
JW2556	*rseA*	Anti-Sigma-E factor	-0.2692	0.0009
JW2558	*nadB*	Quinolinate synthase, L-aspartate oxidase (B protein) subunit	0.0648	0.2283
JW2560	*srmB*	ATP-dependent RNA helicase	-0.0270	0.5188
JW2561	*yfiE*	Putative DNA-binding transcriptional regulator	0.0678	0.1808
JW2562	*yfiK*	Cysteine and O-acetylserine exporter	-0.0385	0.2636
JW2563	*yfiD*	Autonomous glycyl radical cofactor	0.0199	0.7073
JW2564	*ung*	Uracil-DNA-glycosylase	0.0283	0.4322
JW2565	*yfiF*	Putative methyltransferase	0.0318	0.5326
JW2566	*trxC*	Thioredoxin 2	0.0420	0.3412
JW2570	*yfiM*	Putative lipoprotein	-0.0535	0.2594
JW2571	*kgtP*	Alpha-ketoglutarate transporter	0.0190	0.6870
JW2573	*clpB*	Chaperone protein clpb	0.0033	0.9240
JW2576	*rluD*	23S rrna pseudouridine(1911,1915,1917) synthase	-0.0734	0.0068
JW2578	*yfiA*	Cold shock protein associated with 30S ribosomal subunit	0.0443	0.2199
JW2579	*pheL*	Phea gene leader peptide	-0.0632	0.2667
JW2580	*pheA*	Chorismate mutase and prephenate dehydratase, P-protein	0.0640	0.0039
JW2581	*tyrA*	Fused chorismate mutase T/prephenate dehydrogenase	0.1346	0.0123
JW2582	*aroF*	Phospho-2-dehydro-3-deoxyheptonate aldolase, Tyr-sensitive	0.2357	0.0086
JW2584	*yfiR*	Putative periplasmic inhibitor of yfin activity	0.0361	0.3432
JW2585	*yfiN*	Putative membrane-anchored diguanylate cyclase	0.0793	0.0022
JW2592	*ypjD*	Cytochrome c assembly protein family inner membrane protein	-0.0245	0.4869
JW2598	*smpA*	Lipoprotein component of bamabcde OM biogenesis complex	-0.1055	0.0442

JW2599	*yfjF*	UPF0125 family protein	-0.2679	0.0005
JW2600	*yfjG*	Toxic UPF0083 family protein inhibitor of 70S ribosome formation	-0.0068	0.8891
JW2601	*smpB*	Tmrna-binding trans-translation protein	0.0386	0.3821
JW2602	*intA*	CP4-57 prophage; integrase	-0.0105	0.7638
JW2603	*yfjH*	CP4-57 prophage; uncharacterized protein	0.0305	0.4240
JW2604	*alpA*	CP4-57 prophage; DNA-binding transcriptional activator	-0.0365	0.3408
JW2605	*yfjI*	CP4-57 prophage; uncharacterized protein	-0.0330	0.1780
JW2607	*yfjJ*	CP4-57 prophage; uncharacterized protein	0.0219	0.3878
JW2608	*yfjK*	Radiation resistance protein; DEAD/H helicase-like protein; CP4-57 putative defective prophage	0.0105	0.7021
JW2609	*yfjL*	CP4-57 putative defective prophage, DUF4297/DUF1837 polymorphic toxin family protein	-0.0318	0.3101
JW2610	*yfjM*	CP4-57 prophage; uncharacterized protein	0.0633	0.2491
JW2611	*yfjN*	CP4-57 prophage; rnase LS	-0.0126	0.6442
JW2614	*yfjQ*	CP4-57 prophage; uncharacterized protein	-0.0329	0.4331
JW2615	*yfjR*	CP4-57 prophage; putative DNA-binding transcriptional regulator	0.0512	0.0333
JW2618	*yfjT*	CP4-57 prophage; putative periplasmic protein	0.0260	0.5657
JW2619	*yfjU*	CP4-44 prophage; putative disrupted hemin or colicin receptor	-0.0034	0.9204
JW2623	*yfjW*	CP4-57 prophage; putative inner membrane protein	0.0745	0.0825
JW2624	*yfjX*	CP4-57 prophage; putative antirestriction protein	-0.1898	0.0517
JW2625	*yfjY*	CP4-57 prophage; putative DNA repair protein	-0.0472	0.3210
JW2626	*yfjZ*	CP4-57 prophage; antitoxin of the ypjf-yfjz toxin-antitoxin system	0.0336	0.4296
JW2627	*ypjF*	CP4-57 prophage; toxin of the ypjf-yfjz toxin-antitoxin system	-0.0294	0.3146
JW2629	*ypjB*	Pseudogene	-0.0212	0.6136
JW2631	*ygaR*	CP4-44 prophage; putative disrupted hemin or colicin receptor	-0.0194	0.4543
JW2633	*yqaD*	CP4-44 prophage; putative disrupted hemin or colicin receptor	-0.0225	0.5743
JW2635	*ygaF*	L-2-hydroxyglutarate oxidase	0.1146	0.0313
JW2636	*gabD*	Succinate-semialdehyde dehydrogenase I, NADP-dependent	-0.0478	0.1939
JW2637	*gabT*	4-aminobutyrate aminotransferase, PLP-dependent	-0.0998	0.0053
JW2638	*gabP*	Gamma-aminobutyrate transporter	-0.0476	0.3364
JW2639	*csiR*	Transcriptional repressor of csid	0.0700	0.2309
JW2640	*ygaU*	Uncharacterized protein	0.0815	0.0741
JW2641	*yqaE*	UPF0057 membrane protein yqae	0.0851	0.0490
JW2642	*ygaV*	Tributyltin-inducible repressor of ygavp	-0.0934	0.0457
JW2643	*ygaP*	DUF2892 family inner membrane rhodanese	-0.0040	0.9347
JW2644	*stpA*	DNA binding protein, nucleoid-associated	0.0001	0.9979

JW2645	*ygaW*	Alanine exporter, alanine-inducible, stress-responsive	-0.0385	0.5726
JW2646	*ygaC*	Uncharacterized protein	-0.0002	0.9952
JW2647	*ygaM*	Putative membrane-anchored DUF883 family ribosome-binding protein	0.0384	0.2014
JW2648	*nrdH*	Hydrogen donor for nrdef electron transport system; glutaredoxin-like protein	-0.0126	0.7366
JW2649	*nrdI*	Nrdef cluster assembly flavodoxin	-0.0348	0.4463
JW2650	*nrdE*	Ribonucleoside-diphosphate reductase 2, alpha subunit	-0.0090	0.8083
JW2651	*nrdF*	Ribonucleoside-diphosphate reductase 2, beta subunit, ferritin-like protein	-0.0733	0.0432
JW2652	*proV*	Glycine betaine/proline ABC transporter periplasmic binding protein	0.0767	0.0286
JW2653	*proW*	Glycine betaine/proline ABC transporter permease	-0.0562	0.3131
JW2654	*proX*	Glycine betaine/proline ABC transporter periplasmic binding protein; cold shock protein	-0.1650	0.2619
JW2655	*ygaX*	Pseudogene, major facilitator transporter superfamily	-0.0131	0.7260
JW2657	*ygaZ*	Putative L-valine exporter, norvaline resistance protein	0.0515	0.2581
JW2658	*ygaH*	Putative L-valine exporter, norvaline resistance protein	0.0602	0.1080
JW2659	*mprA*	Transcriptional repressor of microcin B17 synthesis and multidrug efflux	0.0030	0.9245
JW2660	*emrA*	Multidrug efflux system	0.0166	0.4190
JW2661	*emrB*	Multidrug efflux system protein	-0.0263	0.5089
JW2662	*luxS*	S-ribosylhomocysteine lyase	-0.0545	0.1309
JW2663	*gshA*	Glutamate-cysteine ligase	-0.1646	0.0002
JW2664	*yqaA*	COG1238 family inner membrane protein	0.0555	0.0295
JW2665	*yqaB*	Fructose-1-phosphate phosphatase yqab	0.0445	0.2108
JW2667	*alaS*	Alanyl-trna synthetase	0.0630	0.0685
JW2668	*recX*	Regulatory protein for reca	0.0651	0.0248
JW2669	*recA*	DNA recombination and repair protein; ssdna-dependent atpase; synaptase; ssdna and dsdna binding protein; ATP-dependent homologous DNA strand exchanger; recombinase A; lexa autocleavage cofactor	-0.3091	0.0034
JW2670	*ygaD*	Nicotinamide-nucleotide amidohydrolase; NMN amidohydrolase	0.0828	0.0286
JW2671	*mltB*	Membrane-bound lytic murein transglycosylase B	-0.0261	0.4851
JW2673	*srlB*	Glucitol/sorbitol-specific enzyme IIA component of PTS	-0.0469	0.4082
JW2674	*srlD*	Sorbitol-6-phosphate dehydrogenase	-0.0090	0.7525
JW2675	*gutM*	Sorbitol=responsive srl operon transcriptional activator	0.0535	0.1679
JW2676	*srlR*	Sorbitol-inducible srl operon transcriptional repressor	-0.0862	0.1303
JW2680	*norV*	Anaerobic nitric oxide reductase flavorubredoxin	0.0545	0.2316

JW2681	*norW*	NADH:flavorubredoxin oxidoreductase	-0.1069	0.0134
JW2683	*hydN*	Formate dehydrogenase-H, [4Fe-4S] ferredoxin subunit	0.2491	0.0000
JW2686	*ascB*	Cryptic 6-phospho-beta-glucosidase	0.0538	0.2070
JW2687	*hycI*	Protease involved in processing C-terminal end of hyce	-0.0029	0.9314
JW2688	*hycH*	Hydrogenase 3 maturation protein	0.0782	0.0027
JW2689	*hycG*	Hydrogenase 3 and formate hydrogenase complex, hycg subunit	0.0003	0.0351
JW2690	*hycF*	Formate hydrogenlyase complex iron-sulfur protein	0.0424	0.2997
JW2691	*hycE*	Hydrogenase 3, large subunit	-0.0671	0.1198
JW2692	*hycD*	Hydrogenase 3, membrane subunit	-0.0515	0.0811
JW2693	*hycC*	Hydrogenase 3, membrane subunit	0.0696	0.1588
JW2694	*hycB*	Hydrogenase 3, Fe-S subunit	0.0220	0.6175
JW2695	*hycA*	Regulator of the transcriptional regulator fhla	-0.0231	0.4232
JW2696	*hypA*	Protein involved in nickel insertion into hydrogenases 3	0.0317	0.5362
JW2697	*hypB*	GTP hydrolase involved in nickel liganding into hydrogenases	0.0316	0.4814
JW2698	*hypC*	Hydrogenase maturation protein	-0.0041	0.8465
JW2699	*hypD*	Hydrogenase maturation protein	0.1448	0.0275
JW2700	*hypE*	Carbamoyl dehydratase, hydrogenases 1,2,3 maturation protein	0.0134	0.7693
JW2701	*fhlA*	Formate hydrogenlyase transcriptional activator	0.0260	0.2524
JW2702	*ygbA*	Uncharacterized protein	0.0200	0.4663
JW2703	*mutS*	Methyl-directed mismatch repair protein	-0.0070	0.8219
JW2704	*pphB*	Serine/threonine-specific protein phosphatase 2	0.0212	0.4275
JW2705	*ygbI*	Deor family putative transcriptional regulator	0.0471	0.4489
JW2706	*ygbJ*	Putative dehydrogenase	-0.0097	0.8679
JW2707	*ygbK*	Flia-regulated DUF1537 family protein	0.0005	0.9890
JW2708	*ygbL*	Putative class II aldolase	-0.0371	0.4045
JW2709	*ygbM*	Putative hydroxypyruvate isomerase	-0.0114	0.6943
JW2710	*ygbN*	Putative transporter	0.0255	0.6103
JW2712	*nlpD*	Activator of amic murein hydrolase activity, lipoprotein	0.1503	0.0177
JW2713	*pcm*	L-isoaspartate protein carboxylmethyltransferase type II	0.0165	0.6681
JW2714	*surE*	Broad specificity 5'(3')-nucleotidase and polyphosphatase	0.0114	0.7167
JW2715	*truD*	Trna(Glu) pseudouridine(13) synthase	-0.0237	0.5832
JW2719	*ygbE*	DUF3561 family inner membrane protein	0.0477	0.2328
JW2720	*cysC*	Adenosine 5'-phosphosulfate kinase	0.0022	0.9434
JW2721	*cysN*	Sulfate adenylyltransferase, subunit 1	0.0562	0.0888
JW2722	*cysD*	Sulfate adenylyltransferase, subunit 2	-0.0526	0.1426
JW2723	*iap*	Aminopeptidase in alkaline phosphatase isozyme conversion	0.0872	0.0764

JW2725	*ygbT*	Multifunctional endonuclease Cas1, CRISPR adaptation protein; DNA repair enzyme	0.0641	0.0319
JW2726	*ygcH*	CRISPR RNA precursor cleavage enzyme; CRISP RNA (crrna) containing Cascade antiviral complex protein	0.0213	0.6384
JW2728	*ygcJ*	CRISP RNA (crrna) containing Cascade antiviral complex protein	0.0355	0.2601
JW2729	*ygcK*	CRISP RNA (crrna) containing Cascade antiviral complex protein	0.0094	0.8274
JW2730	*ygcL*	CRISP RNA (crrna) containing Cascade antiviral complex protein	-0.0560	0.2611
JW2731	*ygcB*	Cascade complex anti-viral R-loop helicase-annealase Cas3	0.0120	0.8167
JW2732	*cysH*	Phosphoadenosine phosphosulfate reductase; PAPS reductase, thioredoxin dependent	0.0358	0.1030
JW2733	*cysI*	Sulfite reductase, beta subunit, NAD(P)-binding, heme-binding	0.0000	0.9991
JW2734	*cysJ*	Sulfite reductase, alpha subunit, flavoprotein	0.0181	0.4699
JW2735	*ygcM*	6-pyruvoyl tetrahydrobiopterin synthase (PTPS)	-0.0595	0.0382
JW2736	*ygcN*	Putative oxidoreductase	-0.0216	0.6457
JW2737	*ygcO*	Putative 4Fe-4S cluster-containing protein	-0.0158	0.6343
JW2738	*ygcP*	Putative antiterminator regulatory protein	-0.0465	0.1225
JW2746	*yqcE*	Putative MFS transporter, inner membrane protein	-0.0093	0.7754
JW2748	*ygcF*	7-carboxy-7-deazaguanine synthase; queosine biosynthesis	-0.0045	0.8793
JW2752	*mazG*	Nucleoside triphosphate pyrophosphohydrolase	0.0257	0.4659
JW2753	*chpA*	Mrna interferase toxin, antitoxin is maze	-0.0039	0.9392
JW2755	*relA*	(p)ppgpp synthetase I/GTP pyrophosphokinase	0.0418	0.1676
JW2756	*rumA*	23S rrna m(5)U1939 methyltransferase, SAM-dependent	-0.0441	0.1709
JW2757	*barA*	Hybrid sensory histidine kinase, in two-component regulatory system with uvry	-0.0724	0.1453
JW2758	*gudD*	D-glucarate dehydratase 1	0.0644	0.0644
JW2759	*gudX*	Glucarate dehydratase-related protein, substrate unknown	0.0520	0.2032
JW2760	*gudP*	Putative D-glucarate transporter	-0.0934	0.0079
JW2761	*yqcA*	Short-chain flavodoxin, FMN-binding	-0.0620	0.0032
JW2762	*yqcB*	Trna(Ile1,Asp) pseudouridine(65) synthase	0.0342	0.3596
JW2763	*yqcC*	DUF446 family protein	-0.0157	0.6183
JW2764	*syd*	Secy-interacting protein	0.0212	0.4908
JW2765	*yqcD*	7-cyano-7-deazaguanine reductase (NADPH-dependent)	0.0891	0.1307
JW2766	*ygdH*	Pyrimidine/purine nucleotide 5'-monophosphate nucleosidase	0.1002	0.0008
JW2767	*sdaC*	Putative serine transporter	0.1326	0.0014
JW2768	*sdaB*	L-serine dehydratase 2	0.0422	0.1163
JW2770	*fucO*	L-1,2-propanediol oxidoreductase	0.0743	0.0779
JW2771	*fucA*	L-fuculose-1-phosphate aldolase	0.0736	0.1091

JW2772	*fucP*	L-fucose transporter	0.0941	0.0003
JW2773	*fucI*	L-fucose isomerase	0.0374	0.2573
JW2774	*fucK*	L-fuculokinase	0.0218	0.5427
JW2775	*fucU*	L-fucose mutarotase	0.0437	0.3357
JW2776	*fucR*	L-fucose operon activator	-0.0818	0.1068
JW2777	*ygdE*	23S rrna C2498 2'-O-ribose methyltransferase, SAM-dependent	-0.0039	0.9243
JW2778	*ygdD*	UPF0382 family inner membrane protein	0.0825	0.0164
JW2779	*gcvA*	Glycine cleavage system transcriptional activator; autorepressor	0.0101	0.6913
JW2781	*csdA*	Cysteine sulfinate desulfinase	0.0503	0.2882
JW2782	*ygdK*	Csda-binding activator; Fe-S protein	0.0129	0.5507
JW2783	*ygdL*	Trna threonylcarbamoyladenosine dehydratase	0.0354	0.3484
JW2784	*mltA*	Membrane-bound lytic murein transglycosylase A	-0.0282	0.4071
JW2786	*argA*	Amino acid N-acetyltransferase and inactive acetylglutamate kinase	0.0654	0.1168
JW2787	*recD*	Exonuclease V (recbcd complex), alpha chain	-0.1991	0.0012
JW2788	*recB*	Exonuclease V (recbcd complex), beta subunit	-0.0227	0.4877
JW2789	*ptr*	Protease 3	0.1460	0.0009
JW2790	*recC*	Exonuclease V (recbcd complex), gamma chain	-0.0324	0.3326
JW2791	*ppdC*	Putative prepilin peptidase-dependent protein	-0.0120	0.6783
JW2794	*ppdA*	Putative prepilin peptidase-dependent protein	0.0393	0.0917
JW2795	*thyA*	Thymidylate synthetase	0.0009	0.0065
JW2797	*ptsP*	PEP-protein phosphotransferase enzyme I; GAF domain containing protein	-0.0199	0.6737
JW2798	*nudH*	RNA pyrophosphohydrolase	0.0161	0.6190
JW2799	*mutH*	Methyl-directed mismatch repair protein	-0.0799	0.0588
JW2800	*ygdQ*	UPF0053 family inner membrane protein	0.0631	0.0477
JW2801	*ygdR*	DUF903 family verified lipoprotein	-0.1561	0.0154
JW2802	*tas*	Putative NADP(H)-dependent aldo-keto reductase	-0.0602	0.0601
JW2803	*ygeD*	Lysophospholipid transporter	0.0059	0.8986
JW2804	*aas*	Fused 2-acylglycerophospho-ethanolamine acyl transferase/acyl-acyl carrier protein synthetase	0.0624	0.0019
JW2805	*galR*	Galactose-inducible d-galactose regulon transcriptional repressor; autorepressor	-0.1186	0.0161
JW2806	*lysA*	Diaminopimelate decarboxylase, PLP-binding	0.0246	0.6348
JW2807	*lysR*	Transcriptional activator of lysa; autorepressor	-0.1195	0.1635
JW2808	*ygeA*	Asp/Glu_racemase family protein	-0.0397	0.3680
JW2809	*araE*	Arabinose transporter	0.0396	0.1543
JW2810	*kduD*	2-dehydro-3-deoxy-D-gluconate 5-dehydrogenase; KDG oxidoreductase; 20-ketosteroid reductase	-0.0552	0.1988
JW2811	*kduI*	Hexuronate isomerase	-0.0947	0.0529
JW2813	*yqeG*	Putative transporter	-0.0385	0.5636

JW2815	*yqeI*	Putative transcriptional regulator	0.0290	0.2946
JW2817	*yqeK*	Uncharacterized protein	-0.0273	0.5006
JW2818	*ygeF*	Pseudogene	-0.0295	0.2901
JW2819	*ygeG*	Sycd-like chaperone family TPR-repeat-containing protein	0.0659	0.0463
JW2820	*ygeH*	Putative transcriptional regulator	-0.0140	0.6600
JW2824	*ygeL*	CP4-44 prophage; putative disrupted hemin or colicin receptor	-0.0571	0.1548
JW2831	*ygeP*	Pseudogene, glycosyl hydrolase family 15, part of T3SS PAI ETT2 remnant	0.0654	0.0827
JW2833	*ygeR*	Lysm domain-containing M23 family putative peptidase; septation lipoprotein	-0.0677	0.0938
JW2835	*xdhB*	Xanthine dehydrogenase, FAD-binding subunit	-0.0932	0.0127
JW2836	*xdhC*	Xanthine dehydrogenase, Fe-S binding subunit	-0.0055	0.8956
JW2837	*ygeV*	Putative sigma-54-interacting transcriptional activator	-0.0806	0.0068
JW2839	*ygeX*	2,3-diaminopropionate ammonia lyase, PLP-dependent	-0.0033	0.9488
JW2840	*ygeY*	Putative peptidase	-0.0354	0.2497
JW2842	*yqeA*	Putative amino acid kinase	-0.0185	0.5182
JW2843	*yqeB*	Xdhc-coxi family protein with NAD(P)-binding Rossman fold	0.0367	0.0183
JW2845	*ygfJ*	CTP:molybdopterin cytidylyltransferase	0.0284	0.4855
JW2848	*ygfM*	Putative oxidoreductase	-0.0979	0.0474
JW2849	*xdhD*	Putative hypoxanthine oxidase, molybdopterin-binding/Fe-S binding	-0.0281	0.5463
JW2850	*ygfO*	Xanthine permease	-0.0102	0.8406
JW2857	*idi*	Isopentenyl diphosphate isomerase	-0.0386	0.1705
JW2858	*lysS*	Lysine trna synthetase, constitutive	-0.0735	0.1835
JW2860	*recJ*	Ssdna exonuclease, 5' --> 3'-specific	0.0372	0.2632
JW2862	*xerD*	Site-specific tyrosine recombinase	0.1678	0.0000
JW2863	*fldB*	Flavodoxin 2	0.0366	0.3536
JW2864	*ygfX*	Inner membrane protein ygfx	-0.0136	0.7049
JW2865	*ygfY*	Flavinator of succinate dehydrogenase; antitoxin of cptab toxin-antitoxin pair	0.0460	0.1489
JW2866	*ygfZ*	Iron-sulfur cluster repair protein, plumbagin resistance	-0.3730	0.0000
JW2868	*yqfB*	UPF0267 family protein	0.0019	0.9600
JW2869	*bglA*	6-phospho-beta-glucosidase A	0.0314	0.2791
JW2870	*ygfF*	Putative NAD(P)-dependent oxidoreductase	-0.0331	0.4277
JW2871	*gcvP*	Glycine decarboxylase, PLP-dependent, subunit P of glycine cleavage complex	-0.0779	0.0850
JW2872	*gcvH*	Glycine cleavage system lipoylprotein H, methylamine group carrier	-0.0115	0.8554
JW2874	*visC*	2-octaprenylphenol hydroxylase, FAD-dependent	0.0100	0.7810
JW2875	*ubiH*	2-octaprenyl-6-methoxyphenol hydroxylase, FAD/NAD(P)-binding	-0.0917	0.0122
JW2878	*zapA*	Ftsz stabilizer	-0.0149	0.7094

JW2879	*ygfA*	5-formyltetrahydrofolate cyclo-ligase family protein	-0.0500	0.2057
JW2880	*serA*	D-3-phosphoglycerate dehydrogenase	0.0418	0.2773
JW2882	*yqfE*	Pseudogene, lysr family	0.0026	0.9376
JW2883	*argP*	Transcriptional regulator for arginine transport and DNA replication genes; replication initiation inhibitor	0.0480	0.3303
JW2885	*argK*	Membrane atpase/protein kinase	-0.1024	0.0286
JW2886	*ygfG*	Methylmalonyl-coa decarboxylase, biotin-independent	-0.0044	0.8645
JW2887	*ygfH*	Propionyl-coa:succinate coa transferase	-0.0236	0.6028
JW2889	*yggE*	Oxidative stress defense protein	0.0302	0.4667
JW2891	*mscS*	Mechanosensitive channel protein, small conductance	0.0407	0.1936
JW2894	*epd*	D-erythrose 4-phosphate dehydrogenase	-0.0767	0.0296
JW2895	*yggC*	Putative pank family P-loop kinase	0.0364	0.4825
JW2896	*yggD*	Fumarase E; mtlr family putative transcriptional repressor	0.1363	0.0087
JW2897	*yggF*	Fructose 1,6 bisphosphatase isozyme	0.0525	0.3046
JW2900	*cmtA*	Putative mannitol-specific PTS IIB and IIC components	-0.0161	0.4705
JW2901	*cmtB*	Putative mannitol-specific enzyme IIA component of PTS	-0.0528	0.3874
JW2903	*yggG*	Phe-Phe periplasmic metalloprotease, OM lipoprotein; low salt-inducible; Era-binding heat shock protein	0.0236	0.6649
JW2904	*speB*	Agmatinase	0.0366	0.1061
JW2905	*speA*	Biosynthetic arginine decarboxylase, PLP-binding	0.0027	0.9491
JW2906	*yqgB*	Uncharacterized protein	-0.0059	0.8495
JW2907	*yqgC*	Uncharacterized protein	-0.0052	0.8036
JW2910	*galP*	D-galactose transporter	-0.0829	0.0000
JW2911	*sprT*	Zn-dependent metalloprotease-related protein	0.0109	0.7658
JW2912	*endA*	DNA-specific endonuclease I	0.0977	0.1298
JW2913	*yggJ*	16S rrna m(3)U1498 methyltransferase, SAM-dependent	0.0000	0.9990
JW2914	*gshB*	Glutathione synthetase	-0.1027	0.0119
JW2915	*yqgE*	Uncharacterized protein	0.0261	0.3972
JW2917	*yggR*	Putative pilt family AAA+ atpase	-0.0221	0.6135
JW2918	*yggS*	UPF0001 family protein, PLP-binding	-0.0099	0.7370
JW2919	*yggT*	Putative inner membrane protein; compensates for loss in K+ uptake	-0.0619	0.1593
JW2921	*yggV*	Ditp/XTP pyrophosphatase	0.0120	0.8294
JW2922	*yggW*	Hemn family putative oxidoreductase	0.0679	0.1092
JW2924	*ansB*	Periplasmic L-asparaginase 2	0.2037	0.0009
JW2925	*yggN*	DUF2884 family putative periplasmic protein	-0.0387	0.2298
JW2926	*yggL*	DUF469 family protein	0.0058	0.8814
JW2927	*yggH*	Trna m(7)G46 methyltransferase, SAM-dependent	0.0354	0.4313

JW2928	*mutY*	Adenine DNA glycosylase	-0.0839	0.0580
JW2929	*yggX*	Oxidative damage protective factor for iron-sulfur proteins	0.0818	0.0056
JW2932	*nupG*	Nucleoside transporter	0.0332	0.4163
JW2934	*yqgA*	DUF554 family putative inner membrane protein	0.1192	0.0394
JW2935	*yghD*	Putative membrane-anchored secretion pathway M-type protein	-0.0109	0.6928
JW2938	*yghG*	Secretin (gspdbeta) OM localization lipoprotein pilotin	0.0294	0.4830
JW2939	*pppA*	Bifunctional prepilin leader peptidase/ methylase	-0.0708	0.0548
JW2942	*yghK*	Glycolate transporter	0.0803	0.1016
JW2943	*glcB*	Malate synthase G	0.0331	0.2720
JW2944	*glcG*	DUF336 family protein	-0.0476	0.1107
JW2946	*glcD*	Glycolate oxidase subunit, FAD-linked	0.0468	0.1648
JW2947	*glcC*	Glc operon transcriptional activator	-0.0162	0.5518
JW2952	*yghR*	Putative ATP-binding protein	-0.0035	0.9402
JW2954	*yghT*	Putative ATP-binding protein	-0.0673	0.0382
JW2955	*pitB*	Phosphate transporter	0.0323	0.3495
JW2956	*gss*	Glutathionylspermidine amidase and glutathionylspermidine synthetase	0.0172	0.6429
JW2958	*hybG*	Hydrogenase 2 accessory protein	0.0143	0.8070
JW2960	*hybE*	Hydrogenase 2-specific chaperone	-0.0398	0.1900
JW2961	*hybD*	Maturation protease for hydrogenase 2	-0.0631	0.0796
JW2962	*hybC*	Hydrogenase 2, large subunit	-0.0639	0.1417
JW2964	*hybA*	Hydrogenase 2 4Fe-4S ferredoxin-type component	-0.0213	0.6286
JW2965	*hybO*	Hydrogenase 2, small subunit	-0.0275	0.5059
JW2970	*yghZ*	L-glyceraldehyde 3-phosphate reductase	0.0326	0.2921
JW2971	*yqhA*	UPF0114 family putative inner membrane protein	-0.0733	0.0083
JW2972	*yghA*	Putative oxidoreductase	0.0454	0.3553
JW2973	*exbD*	Membrane spanning protein in tonb-exbb-exbd complex	-0.1472	0.0095
JW2974	*exbB*	Membrane spanning protein in tonb-exbb-exbd complex	0.0922	0.0123
JW2975	*metC*	Cystathionine beta-lyase, PLP-dependent	-0.3050	0.0131
JW2976	*yghB*	General envelope maintenance protein; deda family inner membrane protein	0.0060	0.8520
JW2978	*yqhD*	Aldehyde reductase, NADPH-dependent	-0.0735	0.1225
JW2985	*sufI*	Cell division protein ftsp	0.0746	0.0821
JW2987	*parC*	DNA topoisomerase IV, subunit A	0.0258	0.4515
JW2990	*ygiU*	GCU-specific mrna interferase toxin of the mqsr-mqsa toxin-antitoxin system; biofilm/motility regulator; anti-repressor	-0.0600	0.2159
JW2992	*ygiW*	Hydrogen peroxide and cadmium resistance periplasmic protein; stress-induced OB-fold protein	-0.0404	0.3417

JW2993	*qseB*	Quorum sensing DNA-binding response regulator in two-component regulatory system with qsec	-0.0560	0.2588
JW2994	*qseC*	Quorum sensing sensory histidine kinase in two-component regulatory system with qseb	-0.0369	0.2829
JW2995	*ygiZ*	Inner membrane protein	0.0604	0.1961
JW2996	*mdaB*	NADPH quinone reductase	-0.0062	0.8745
JW2997	*ygiN*	Quinol monooxygenase	-0.0461	0.3249
JW2999	*yqiA*	Acyl coa esterase	-0.0246	0.4464
JW3000	*cpdA*	3',5' camp phosphodiesterase	-0.0009	0.9836
JW3001	*yqiB*	DUF1249 protein yqib	-0.0290	0.3382
JW3002	*nudF*	ADP-ribose pyrophosphatase	-0.0508	0.0715
JW3006	*ygiC*	ATP-Grasp family atpase	0.1246	0.0112
JW3007	*zupT*	4,5- DOPA-extradiol-dioxygenase	0.0139	0.5829
JW3008	*ygiE*	Zinc transporter	0.0012	0.0090
JW3011	*ygiL*	Putative fimbrial-like adhesin protein	0.0677	0.0498
JW3021	*glgS*	Motility and biofilm regulator	0.0068	0.8846
JW3022	*yqiJ*	DUF1449 family inner membrane protein	0.0558	0.1012
JW3023	*yqiK*	PHB family membrane protein, function unknown	-0.0356	0.2950
JW3024	*rfaE*	Heptose 7-phosphate kinase and heptose 1-phosphate adenyltransferase	-0.0827	0.0009
JW3025	*glnE*	Fused deadenylyltransferase/adenylyltransferase for glutamine synthetase	0.0120	0.6934
JW3026	*ygiF*	Inorganic triphosphatase	-0.0236	0.4560
JW3027	*htrG*	SH3 domain protein	0.0191	0.6793
JW3029	*bacA*	Undecaprenyl pyrophosphate phosphatase	-0.0266	0.5152
JW3030	*folB*	Dihydroneopterin aldolase and dihydroneopterin triphosphate 2'-epimerase	-0.0993	0.0782
JW3031	*ygiH*	Putative glycerol-3-phosphate acyltransferase	0.0573	0.1545
JW3032	*ygiP*	Transcriptional activator of ttdabt	-0.0455	0.3893
JW3033	*ttdA*	L-tartrate dehydratase, alpha subunit	0.1274	0.0005
JW3034	*ttdB*	L-tartrate dehydratase, beta subunit	0.1063	0.4171
JW3035	*ygjE*	L-tartrate/succinate antiporte	-0.0181	0.6538
JW3037	*rpsU*	30S ribosomal subunit protein S21	-0.0606	0.0503
JW3038	*dnaG*	DNA primase	0.0454	0.2622
JW3039	*rpoD*	RNA polymerase, sigma 70 (sigma D) factor	-0.0038	0.9323
JW3040	*ygjF*	G/U mismatch-specific DNA glycosylase; xanthine DNA glycosylase	0.0156	0.7953
JW3041	*yqjH*	Putative siderophore interacting protein	-0.0148	0.6678
JW3042	*yqjI*	Padr family putative transcriptional regulator	-0.0217	0.5174
JW3043	*aer*	Fused signal transducer for aerotaxis sensory component/methyl accepting chemotaxis component	-0.0592	0.0615
JW3045	*ygjH*	Putative trna binding protein; putative trna corner chaperone	0.0912	0.0411
JW3046	*ebgR*	HTH-type transcriptional regulator ebgr	0.0299	0.3419

JW3048	*ebgC*	Evolved beta-D-galactosidase, beta subunit; cupin superfamily	-0.0291	0.5647
JW3050	*ygjJ*	Putative periplasmic protein	0.0644	0.2021
JW3053	*ygjM*	Antitoxinof the higb-higa toxin-antitoxin system	0.0486	0.1376
JW3054	*ygjN*	Mrna interferase toxin of the higb-higa toxin-antitoxin system	0.0390	0.5998
JW3057	*ygjQ*	DUF218 superfamily protein	-0.1216	0.0050
JW3058	*ygjR*	Putative NAD(P)-dependent dehydrogenase	0.0420	0.3418
JW3060	*sstT*	Serine/threonine transporter sstt	-0.0289	0.3995
JW3061	*ygjV*	Imp-ygjv family inner membrane protein	-0.0294	0.3660
JW3062	*uxaA*	Altronate hydrolase	-0.0271	0.5398
JW3063	*uxaC*	Uronate isomerase	0.1135	0.0093
JW3064	*exuT*	Hexuronate transporter	0.0416	0.1370
JW3065	*exuR*	Hexuronate regulon transcriptional repressor; autorepressor	-0.0116	0.6038
JW3066	*yqjA*	General envelope maintenance protein; deda family inner membrane protein	0.1375	0.0243
JW3067	*yqjB*	Modulator protein mzra	-0.0214	0.3571
JW3069	*yqjD*	Membrane-anchored ribosome-binding protein	-0.0364	0.2541
JW3071	*yqjK*	Uncharacterized protein	-0.1244	0.0132
JW3073	*yqjG*	Putative S-transferase	0.0546	0.0043
JW3074	*yhaH*	DUF805 family inner membrane protein,	0.1422	0.0310
JW3075	*yhaI*	DUF805 family inner membrane protein	-0.0399	0.3502
JW3076	*yhaJ*	Transcription regulator of quinol-like compound degradation	0.0243	0.3060
JW3077	*yhaK*	Dinitrotoluene degradation protein, redox-sensitive bicupin	0.0820	0.3697
JW3087	*tdcC*	L-threonine/L-serine transporter	0.0347	0.2832
JW3088	*tdcB*	L-threonine dehydratase, catabolic	0.0653	0.0294
JW3089	*tdcA*	Tdc operon transcriptional activator	0.1611	0.1689
JW3091	*yhaB*	Uncharacterized protein	-0.0181	0.5710
JW3092	*yhaC*	Pentapetide repeats-related protein	0.0486	0.2508
JW3093	*garK*	Glycerate kinase I	-0.1727	0.0000
JW3095	*garL*	Alpha-dehydro-beta-deoxy-D-glucarate aldolase	-0.0205	0.6723
JW3096	*garP*	Putative (D)-galactarate transporter	0.0600	0.0820
JW3097	*garD*	D-galactarate dehydrogenase	-0.0706	0.0702
JW3098	*sohA*	Antitoxin of the soha(prlf)-yhav toxin-antitoxin system	-0.0923	0.0475
JW3099	*yhaV*	Toxin of the sohb(prlf)-yhav toxin-antitoxin system	0.0039	0.8878
JW3100	*agaR*	Transcriptional repressor of the aga regulon	0.0768	0.2044
JW3101	*kbaZ*	Tagatose 6-phosphate aldolase 1, kbaz subunit	-0.0451	0.3227
JW3102	*agaV*	N-acetylgalactosamine-specific enzyme IIB component of PTS	0.0649	0.0295
JW3103	*agaW*	CP4-44 prophage; putative disrupted hemin or colicin receptor	0.0344	0.1711
JW3105	*agaS*	Tagatose-6-phosphate ketose/aldose isomerase	-0.0100	0.8071

JW3106	*kbaY*	Tagatose 6-phosphate aldolase 1, kbay subunit	0.0250	0.3419
JW3107	*agaB*	N-acetylgalactosamine-specific enzyme IIB component of PTS	0.0278	0.4742
JW3108	*agaC*	N-acetylgalactosamine-specific enzyme IIC component of PTS	0.0079	0.8600
JW3109	*agaD*	N-acetylgalactosamine-specific enzyme IID component of PTS	0.0468	0.3521
JW3110	*agaI*	Galactosamine-6-phosphate isomerase	-0.0995	0.0173
JW3111	*yraH*	Putative fimbrial-like adhesin protein	0.0769	0.0806
JW3112	*yraI*	Putative periplasmic pilin chaperone	0.1796	0.0002
JW3113	*yraJ*	Putative outer membrane protein	0.0519	0.0258
JW3114	*yraK*	Putative fimbrial-like adhesin protein	0.0202	0.6032
JW3117	*yraN*	UPF0102 family protein	0.0519	0.0717
JW3118	*yraO*	Dnaa initiator-associating factor for replication initiation	-0.0423	0.1762
JW3119	*yraP*	Outer membrane lipoprotein	-0.0328	0.5629
JW3120	*yraQ*	Putative inner membrane permease	0.0282	0.3538
JW3124	*yhbQ*	GIY-YIG nuclease superfamily protein	0.0213	0.1612
JW3125	*yhbS*	GNAT family putative N-acetyltransferase	-0.0207	0.4914
JW3126	*yhbT*	SCP2 domain-containing protein yhbt	0.0425	0.0998
JW3127	*yhbU*	U32 peptidase family protein	0.0355	0.4160
JW3129	*yhbW*	Putative luciferase-like monooxygenase	0.0858	0.0031
JW3130	*mtr*	Tryptophan transporter of high affinity	0.0513	0.1335
JW3134	*rpsO*	30S ribosomal subunit protein S15	0.0266	0.1710
JW3136	*rbfA*	30s ribosome binding factor	-0.3504	0.0000
JW3140	*argG*	Argininosuccinate synthetase	-0.0436	0.1072
JW3143	*glmM*	Phosphoglucosamine mutase	0.0138	0.7560
JW3144	*folP*	7,8-dihydropteroate synthase	0.0626	0.0897
JW3147	*yhbY*	RNA binding protein associated with pre-50S ribosomal subunits	-0.0349	0.3449
JW3148	*greA*	Transcript cleavage factor	-0.0522	0.1285
JW3149	*dacB*	D-alanyl-D-alanine carboxypeptidase	-0.0984	0.0067
JW3151	*yhbE*	Eama family inner membrane putative transporter	0.0163	0.6477
JW3155	*sfsB*	Malpq operon transcriptional activator	-0.0701	0.1454
JW3157	*yrbA*	Acid stress protein; putative bola family transcriptional regulator	0.0963	0.0824
JW3159	*yrbC*	ABC transporter maintaining OM lipid asymmetry, periplasmic binding protein	-0.2489	0.0605
JW3160	*yrbD*	OM lipid asymmetry maintenance protein; membrane-anchored ABC family periplasmic binding protein	0.0933	0.0172
JW3161	*yrbE*	ABC transporter maintaining OM lipid asymmetry, inner membrane permease protein	0.0763	0.1184
JW3162	*yrbF*	ABC transporter maintaining OM lipid asymmetry, ATP-binding protein	0.0523	0.3222
JW3163	*yrbG*	Putative calcium/sodium:proton antiporter	-0.0014	0.9433

JW3164	*kdsD*	D-arabinose 5-phosphate isomerase	-0.0396	0.2582
JW3165	*kdsC*	3-deoxy-D-manno-octulosonate 8-phosphate phosphatase	0.0746	0.1027
JW3168	*yhbG*	Lipopolysaccharide export ABC transporter atpase	0.0514	0.0816
JW3169	*rpoN*	RNA polymerase, sigma 54 (sigma N) factor	0.0010	0.0256
JW3170	*yhbH*	Ribosome hibernation promoting factor HPF; stabilizes 100S dimers	0.0341	0.4452
JW3171	*ptsN*	Sugar-specific enzyme IIA component of PTS	0.0635	0.0146
JW3172	*yhbJ*	Adaptor protein for glmz/glmy srna decay, glucosamine-6-phosphate-regulated; ntpase	-0.0819	0.0745
JW3173	*npr*	Phosphohistidinoprotein-hexose phosphotransferase component of N-regulated PTS system (Npr)	-0.0232	0.6514
JW3174	*yrbL*	Mg(2+)-starvation-stimulated protein	-0.0512	0.1476
JW3176	*elbB*	Isoprenoid biosynthesis protein with amidotransferase-like domain	-0.0546	0.0547
JW3178	*yhcC*	Putative Fe-S oxidoreductase, Radical SAM superfamily protein	0.0666	0.0061
JW3179	*gltB*	Glutamate synthase, large subunit	-0.0143	0.7487
JW3180	*gltD*	Glutamate synthase, 4Fe-4S protein, small subunit	0.0098	0.7487
JW3181	*gltF*	Periplasmic protein	0.0271	0.6361
JW3182	*yhcA*	Putative periplasmic chaperone protein	0.0116	0.7868
JW3183	*yhcD*	Putative outer membrane fimbrial subunit usher protein	0.0608	0.3292
JW3184	*yhcE*	CP4-44 prophage; putative disrupted hemin or colicin receptor	-0.0322	0.4351
JW3187	*yhcE*	CP4-44 prophage; putative disrupted hemin or colicin receptor	-0.0990	0.0074
JW3188	*yhcF*	Putative transcriptional regulator	0.0762	0.1871
JW3189	*yhcG*	DUF1016 family protein in the PD-(D/E)XK nuclease superfamily	-0.0385	0.0850
JW3190	*yhcH*	DUF386 family protein, cupin superfamily	0.0076	0.8679
JW3192	*nanE*	Putative N-acetylmannosamine-6-P epimerase	0.0349	0.4583
JW3193	*nanT*	Sialic acid transporter	0.0209	0.4632
JW3195	*nanR*	Sialic acid-inducible nan operon repressor	0.0531	0.2287
JW3196	*dcuD*	Putative transporter	-0.0835	0.0113
JW3197	*sspB*	Clpxp protease specificity enhancing factor	-0.0030	0.9352
JW3198	*sspA*	Stringent starvation protein A, phage P1 late gene activator, RNAP-associated acid-resistance protein, inactive glutathione S-transferase homolog	-0.2143	0.0002
JW3201	*yhcM*	Divisome atpase	-0.0218	0.5379
JW3203	*degQ*	Serine endoprotease, periplasmic	0.0516	0.1802
JW3205	*mdh*	Malate dehydrogenase, NAD(P)-binding	-0.2868	0.0004
JW3206	*argR*	L-arginine-responsive arginine metabolism regulon transcriptional regulator	-0.1021	0.1984
JW3208	*yhcO*	Putative barnase inhibitor	0.0560	0.1942

JW3209	*aaeB*	P-hydroxybenzoic acid efflux system component	-0.1066	0.4324
JW3210	*aaeA*	P-hydroxybenzoic acid efflux system component	0.0531	0.3872
JW3212	*aaeR*	Transcriptional regulator for aaexab operon	0.0520	0.0813
JW3213	*tldD*	Putative peptidase	-0.0173	0.7311
JW3216	*rng*	Ribonuclease G	0.0097	0.8038
JW3217	*yhdE*	Dttp/UTP pyrophosphatase; m(5)UTP/m(5)CTP/pseudo-UTP pyrophosphatase	-0.0503	0.1996
JW3221	*yhdA*	Targeting factor for csrbc srna degradation	0.0526	0.0569
JW3222	*yhdH*	Putative acryloyl-coa reductase	-0.0624	0.0115
JW3225	*yhdT*	DUF997 family putative inner membrane protein	-0.0129	0.6565
JW3226	*panF*	Pantothenate:sodium symporter	-0.0704	0.3051
JW3227	*prmA*	Methyltransferase for 50S ribosomal subunit protein L11	-0.0318	0.3076
JW3228	*dusB*	Trna-dihydrouridine synthase B	-0.0225	0.6458
JW3229	*fis*	Global DNA-binding transcriptional dual regulator	-0.0206	0.7916
JW3231	*yhdU*	Putative membrane protein	0.0741	0.1218
JW3232	*envR*	Acrab operon transcriptional repressor	0.0355	0.3275
JW3233	*acrE*	Cytoplasmic membrane lipoprotein	-0.0046	0.8529
JW3234	*acrF*	Multidrug efflux system protein	-0.0358	0.4058
JW3235	*yhdV*	Putative outer membrane protein	-0.0583	0.2374
JW3236	*yhdW*	Pseudogene, ABC transporter periplasmic binding protein family	0.0145	0.6289
JW3239	*yhdZ*	Putative amino acid ABC transporter atpase	0.0589	0.0103
JW3241	*yrdB*	DUF1488 family protein	0.0119	0.7179
JW3242	*aroE*	Dehydroshikimate reductase, NAD(P)-binding	-0.0208	0.6470
JW3245	*smg*	DUF494 family putative periplasmic protein	-0.0635	0.0090
JW3250	*rsmB*	16S rrna m(5)C967 methyltransferase, SAM-dependent	-0.0588	0.2699
JW3251	*trkA*	NAD-binding component of trk potassium transporter	-0.1374	0.0026
JW3252	*mscL*	Mechanosensitive channel protein, high conductance	-0.0377	0.1792
JW3253	*yhdL*	Alternate ribosome-rescue factor A	-0.0448	0.3058
JW3254	*zntR*	Znta gene transcriptional activator	0.0151	0.7407
JW3255	*yhdN*	DUF1992 family protein	0.0521	0.0910
JW3261	*rpmJ*	50S ribosomal subunit protein L36	-0.1077	0.0976
JW3284	*pioO*	Part of gsp divergon involved in type II protein secretion	-0.1514	0.0027
JW3285	*gspA*	General secretory pathway component, cryptic	-0.1990	0.0070
JW3288	*gspE*	General secretory pathway component, cryptic	-0.0001	0.9987
JW3289	*gspF*	General secretory pathway component, cryptic	0.0094	0.7666
JW3290	*gspG*	Pseudopilin, cryptic, general secretion pathway	-0.0619	0.1478
JW3291	*gspH*	Putative general secretory pathway component, cryptic	0.0339	0.2964
JW3293	*gspJ*	Putative general secretory pathway component, cryptic	-0.0170	0.5611

JW3294	*gspK*	General secretory pathway component, cryptic	-0.0808	0.0205
JW3297	*gspO*	Bifunctional prepilin leader peptidase/ methylase	0.0169	0.6660
JW3298	*bfr*	Bacterioferritin, iron storage and detoxification protein	-0.1702	0.0076
JW3299	*bfd*	Bacterioferritin-associated ferredoxin	0.0107	0.7571
JW3300	*chiA*	Periplasmic endochitinase	-0.0771	0.0640
JW3301	*tufA*	Translation elongation factor EF-Tu 1	0.0433	0.3303
JW3305	*yheL*	Mnm(5)-s(2)U34-trna synthesis 2-thiolation protein	-0.0960	0.0035
JW3306	*yheM*	Mnm(5)-s(2)U34-trna synthesis 2-thiolation protein	-0.0018	0.9684
JW3307	*yheN*	Sulfurtransferase for 2-thiolation step of mnm(5)-s(2)U34-trna synthesis	-0.0054	0.8679
JW3309	*fkpA*	FKBP-type peptidyl-prolyl cis-trans isomerase (rotamase)	0.0333	0.3514
JW3310	*slyX*	Phi X174 lysis protein	0.0601	0.0136
JW3311	*slyD*	FKBP-type peptidyl prolyl cis-trans isomerase (rotamase)	-0.2730	0.0025
JW3312	*yheV*	DUF2387 family putative metal-binding protein	0.0314	0.4316
JW3313	*kefB*	Potassium:proton antiporter	0.0592	0.0207
JW3315	*yheS*	ABC-F family protein predicted regulatory atpase	0.0416	0.0859
JW3316	*yheT*	UPF0017 family putative hydrolase	-0.0365	0.4646
JW3317	*yheU*	UPF0270 family protein	0.0111	0.7572
JW3318	*prkB*	Putative phosphoribulokinase	0.0329	0.0959
JW3319	*yhfA*	Osmc family protein	0.0120	0.6338
JW3322	*argD*	Bifunctional acetylornithine aminotransferase and succinyldiaminopimelate aminotransferase	0.1200	0.0438
JW3323	*pabA*	Aminodeoxychorismate synthase, subunit II	-0.2237	0.0000
JW3324	*fic*	Stationary phase-induced protein, putative toxin	0.0135	0.6733
JW3325	*yhfG*	Putative antitoxin for Fic	0.0151	0.6320
JW3326	*ppiA*	Peptidyl-prolyl cis-trans isomerase A (rotamase A)	0.0085	0.7084
JW3327	*tsgA*	Putative transporter	-0.1586	0.1887
JW3328	*nirB*	Nitrite reductase, large subunit, NAD(P)H-binding	-0.0588	0.0150
JW3329	*nirD*	Nitrite reductase (NADH) small subunit	0.0442	0.0424
JW3330	*nirC*	Nitrite transporter	0.0638	0.1358
JW3331	*cysG*	Fused siroheme synthase 1,3-dimethyluroporphyriongen III dehydrogenase and siroheme ferrochelatase/uroporphyrinogen methyltransferase	-0.0877	0.0026
JW3332	*yhfL*	Small lipoprotein	-0.0710	0.0639
JW3333	*frlA*	Putative fructoselysine transporter	-0.0154	0.7479
JW3337	*frlD*	Fructoselysine 6-kinase	0.0173	0.6910
JW3340	*yhfT*	Inner membrane protein	-0.0106	0.7484
JW3342	*php*	Phosphotriesterase homology protein	0.1252	0.1693

JW3343	*yhfW*	Phosphopentomutase-related metalloenzyme superfamily protein	-0.0144	0.6436
JW3344	*yhfX*	Putative pyridoxal 5'-phosphate binding protein	0.0207	0.6729
JW3348	*gph*	Phosphoglycolate phosphatase	0.1349	0.0005
JW3349	*rpe*	D-ribulose-5-phosphate 3-epimerase	-0.1172	0.0008
JW3350	*dam*	DNA adenine methyltransferase	0.0636	0.1320
JW3351	*damX*	Cell division protein that binds to the septal ring	0.1387	0.0000
JW3352	*aroB*	3-dehydroquinate synthase	0.1112	0.0260
JW3354	*hofQ*	DNA catabolic putative fimbrial transporter	0.0475	0.4329
JW3356	*yrfB*	DNA catabolic protein	0.0598	0.0436
JW3357	*yrfC*	DNA catabolic putative fimbrial assembly protein	0.0094	0.7177
JW3359	*mrcA*	Penicillin-binding protein 1a, murein transglycosylase and transpeptidase	-0.0457	0.0930
JW3360	*nudE*	Adenosine nucleotide hydrolase; Ap3A/Ap2A/ADP-ribose/NADH hydrolase	-0.0093	0.7194
JW3363	*hslR*	Ribosome-associated heat shock protein Hsp15	-0.0898	0.0186
JW3365	*yhgE*	DUF4153 family putative inner membrane protein	0.0902	0.0746
JW3366	*pck*	Phosphoenolpyruvate carboxykinase [ATP]	-0.0800	0.0886
JW3367	*envZ*	Sensory histidine kinase in two-component regulatory system with ompr	-0.2475	0.0695
JW3368	*ompR*	Response regulator in two-component regulatory system with envz	0.0862	0.1170
JW3369	*greB*	Transcript cleavage factor	0.0325	0.0675
JW3370	*yhgF*	Putative transcriptional accessory factor; ionizing radiation survival protein; putative nucleic acid-binding protein	-0.0288	0.0347
JW3371	*feoA*	Ferrous iron transporter, protein A	-0.0315	0.4575
JW3372	*feoB*	Ferrous iron transporter protein B and GTP-binding protein; membrane protein	-0.0410	0.2954
JW3373	*yhgG*	Putative DNA-binding transcriptional regulator	-0.0411	0.3061
JW3374	*yhgA*	Transposase_31 family protein	-0.1253	0.0383
JW3375	*bioH*	Pimeloyl-ACP methyl ester carboxylesterase	0.1937	0.0003
JW3377	*gntY*	Fe/S biogenesis protein, scaffold/chaperone protein	-0.0887	0.0037
JW3379	*malQ*	4-alpha-glucanotransferase (amylomaltase)	-0.0227	0.5882
JW3381	*malT*	Mal regulon transcriptional activator	-0.0200	0.4877
JW3384	*rtcB*	RNA-splicing ligase	-0.0048	0.8797
JW3385	*rtcR*	Sigma 54-dependent transcriptional regulator of rtcba expression	-0.0405	0.0316
JW3386	*glpR*	Pseudogene, DNA-binding transcriptional repressor	-0.0041	0.9010
JW3388	*glpE*	Thiosulfate:cyanide sulfurtransferase (rhodanese)	0.1070	0.0064
JW3389	*glpD*	Sn-glycerol-3-phosphate dehydrogenase, aerobic, FAD/NAD(P)-binding	0.0423	0.4555
JW3390	*yzgL*	Pseudogene, periplasmic solute binding protein homology	-0.0078	0.8515
JW3392	*glgA*	Glycogen synthase	0.0143	0.7996

JW3393	*glgC*	Glucose-1-phosphate adenylyltransferase	-0.1134	0.0232
JW3394	*glgX*	Glycogen debranching enzyme	-0.1233	0.0099
JW3395	*glgB*	1,4-alpha-glucan branching enzyme	-0.0293	0.1904
JW3397	*yhgN*	UPF0056 family inner membrane protein	0.0148	0.7062
JW3400	*gntK*	Gluconate kinase 2	0.0015	0.9529
JW3402	*yhhW*	Quercetinase activity in vitro	-0.0412	0.4314
JW3405	*yhhY*	L-amino acid N-acetyltransferase; aminoacyl nucleotide detoxifying acetyltransferase	0.0791	0.0283
JW3406	*yhhZ*	Putative Hcp1 family polymorphic toxin protein; putative colicin-like dnase/trnase activity	0.0030	0.8868
JW3411	*yrhB*	Stable heat shock chaperone	0.0020	0.8714
JW3412	*ggt*	Gamma-glutamyltranspeptidase	0.0264	0.2163
JW3413	*yhhA*	DUF2756 family protein	0.0944	0.0520
JW3414	*ugpQ*	Glycerophosphodiester phosphodiesterase, cytosolic	0.0220	0.2756
JW3415	*ugpC*	Sn-glycerol-3-phosphate ABC transporter atpase	-0.2898	0.0000
JW3416	*ugpE*	Sn-glycerol-3-phosphate ABC transporter permease	0.0968	0.3218
JW3417	*ugpA*	Sn-glycerol-3-phosphate ABC transporter permease	0.0693	0.1625
JW3418	*ugpB*	Sn-glycerol-3-phosphate-binding periplasmic protein ugpb	0.1001	0.0048
JW3419	*livF*	Branched-chain amino acid ABC transporter atpase	-0.0161	0.6035
JW3420	*livG*	Branched-chain amino acid ABC transporter atpase	0.0633	0.0916
JW3421	*livM*	Branched-chain amino acid ABC transporter permease	0.1273	0.0095
JW3422	*livH*	Branched-chain amino acid ABC transporter permease	-0.0115	0.8237
JW3423	*livK*	Leucine transporter subunit	-0.0337	0.2434
JW3424	*yhhK*	Pand autocleavage accelerator, panothenate synthesis	0.0762	0.2615
JW3425	*livJ*	Branched-chain amino acid ABC transporter periplasmic binding protein	0.0499	0.0395
JW3432	*yhhM*	DUF2500 family protein	-0.0286	0.3845
JW3433	*yhhN*	TMEM86 family putative inner membrane protein	-0.0177	0.6688
JW3434	*zntA*	Zinc, cobalt and lead efflux system	-0.0588	0.3069
JW3435	*yhhP*	Mnm(5)-s(2)U34-trna 2-thiolation sulfurtransferase	-0.0503	0.0956
JW3440	*acpT*	4'-phosphopantetheinyl transferase	-0.0522	0.1470
JW3441	*nikA*	Nickel/heme ABC transporter periplasmic binding protein	-0.0148	0.6728
JW3442	*nikB*	Nickel ABC transporter permease	0.0662	0.0223
JW3443	*nikC*	Nickel ABC transporter permease	0.0047	0.9317
JW3444	*nikD*	Nickel ABC transporter atpase	-0.0878	0.0316
JW3445	*nikE*	Nickel ABC transporter atpase	0.0533	0.1222
JW3446	*nikR*	Transcriptional repressor, Ni-binding	0.0236	0.3363

JW3449	*yhhH*	Putative NTF2 fold immunity protein for polymorphic toxin rhsb	-0.0999	0.0458
JW3451	*yhhI*	Putative transposase	-0.1571	0.0099
JW3454	*yhiI*	Putative membrane fusion protein (MFP) of efflux pump	-0.0920	0.0160
JW3455	*yhiJ*	DUF4049 family protein	0.0553	0.1644
JW3457	*yhiL*	Uncharacterized protein	0.0291	0.5483
JW3459	*yhiN*	Putative oxidoreductase	0.0774	0.0061
JW3460	*pitA*	Phosphate transporter, low-affinity; tellurite importer	-0.0532	0.1787
JW3461	*yhiO*	Universal stress (ethanol tolerance) protein B	-0.0241	0.1737
JW3462	*uspA*	Universal stress global response regulator	0.0364	0.3743
JW3463	*yhiP*	Dipeptide and tripeptide permease B	0.0691	0.4542
JW3465	*prlC*	Oligopeptidase A	-0.0695	0.1069
JW3467	*gor*	Glutathione oxidoreductase	0.1080	0.0265
JW3468	*arsR*	Arsenical resistance operon transcriptional repressor; autorepressor	0.0472	0.2944
JW3469	*arsB*	Arsenite/antimonite transporter	-0.0479	0.3716
JW3470	*arsC*	Arsenate reductase	-0.0598	0.0145
JW3471	*yhiS*	Pseudogene	0.1193	0.1116
JW3474	*slp*	Outer membrane lipoprotein	0.0447	0.1131
JW3478	*hdeA*	Stress response protein acid-resistance protein	0.0121	0.7821
JW3479	*hdeD*	Acid-resistance membrane protein	0.0074	0.7825
JW3480	*gadE*	Gad regulon transcriptional activator	0.0622	0.1526
JW3481	*mdtE*	Anaerobic multidrug efflux transporter, arca-regulated	0.0386	0.1676
JW3482	*mdtF*	Anaerobic multidrug efflux transporter, arca-regulated	0.0447	0.2706
JW3483	*gadW*	Transcriptional activator of gada and gadbc; repressor of gadx	-0.0446	0.1002
JW3484	*gadX*	Acid resistance regulon transcriptional activator; autoactivator	0.0900	0.1959
JW3485	*gadA*	Glutamate decarboxylase A, PLP-dependent	-0.0278	0.4039
JW3486	*yhjA*	Cytochrome c peroxidase	-0.0374	0.5712
JW3487	*treF*	Cytoplasmic trehalase	-0.0986	0.0236
JW3489	*yhjC*	Lysr family putative transcriptional regulator	0.0877	0.0392
JW3491	*yhjE*	Putative MFS transporter; membrane protein	0.0225	0.6694
JW3492	*yhjG*	Putative inner membrane-anchored periplasmic asma family protein	-0.0200	0.4744
JW3495	*yhjJ*	Putative periplasmic M16 family chaperone	0.0223	0.6556
JW3496	*dctA*	C4-dicarboxylic acid, orotate and citrate transporter	0.0517	0.1876
JW3499	*bcsZ*	Endo-1,4-D-glucanase	-0.0453	0.3791
JW3503	*yhjR*	DUF2629 family protein	0.0178	0.6229
JW3504	*bcsE*	Cellulose production protein	-0.1841	0.0000
JW3506	*bcsG*	DUF3260 family cellulose production inner membrane protein	-0.0469	0.2552

JW3508	*yhjV*	Putative transporter	0.0256	0.5442
JW3509	*dppF*	Dipeptide/heme ABC transporter atpas	0.0557	0.1363
JW3510	*dppD*	Dipeptide/heme ABC transporter atpas	0.0633	0.0366
JW3511	*dppC*	Dipeptide transport system permease protein dppc	-0.0596	0.0639
JW3512	*dppB*	Dipeptide/heme ABC transporter permease	0.0442	0.1196
JW3513	*dppA*	Dipeptide/heme ABC transporter periplasmic binding protein; dipeptide chemotaxis receptor	-0.0382	0.4897
JW3516	*yhjX*	Putative MFS antiporter, pyruvate-inducible	-0.0667	0.0530
JW3518	*tag*	3-methyl-adenine DNA glycosylase I, constitutive	-0.0269	0.5064
JW3519	*yiaC*	GNAT family putative N-acetyltransferase	-0.0992	0.0712
JW3524	*yiaG*	HTH_CROC1 family putative transcriptional regulator	0.0105	0.7993
JW3525	*cspA*	RNA chaperone and antiterminator, cold-inducible	-0.0147	0.6899
JW3526	*hokA*	Toxic polypeptide, small	-0.1809	0.0018
JW3530	*glyS*	Glycine trna synthetase, beta subunit	-0.0542	0.2198
JW3532	*ysaB*	Uncharacterized protein	-0.0693	0.1759
JW3533	*yiaH*	O-acetyltransferase for enterobacterial common antigen (ECA)	0.0281	0.4688
JW3534	*yiaA*	Yiaab family inner membrane protein, tandem domains	-0.0185	0.5352
JW3536	*xylB*	Xylulokinase	-0.1032	0.0037
JW3537	*xylA*	D-xylose isomerase	0.0146	0.5030
JW3538	*xylF*	D-xylose transporter subunit	0.0967	0.0686
JW3539	*xylG*	D-xylose ABC transporter dual domain atpase	-0.0615	0.0236
JW3540	*xylH*	D-xylose ABC transporter permease	-0.0362	0.2468
JW3541	*xylR*	Xylose divergent operon transcriptional activator	-0.0050	0.9014
JW3543	*malS*	Alpha-amylase	0.0947	0.1578
JW3545	*yiaI*	Putative hydrogenase, 4Fe-4S ferredoxin-type component	0.0405	0.1763
JW3546	*yiaJ*	Transcriptional repressor for the yiaklmno-lyxk-sgbhue operon	-0.0788	0.4546
JW3547	*yiaK*	2,3-diketo-L-gulonate reductase, NADH-dependent	-0.0272	0.4780
JW3548	*yiaL*	DUF386 family protein	-0.0089	0.7135
JW3549	*yiaM*	2,3-diketo-L-gulonate TRAP transporter small permease protein	-0.0022	0.9374
JW3551	*yiaO*	2,3-diketo-L-gulonate-binding periplasmic protein	0.5996	0.0047
JW3552	*lyxK*	L-xylulose kinase	-0.0476	0.2286
JW3553	*sgbH*	3-keto-L-gulonate 6-phosphate decarboxylase	0.0247	0.4148
JW3555	*sgbE*	L-ribulose-5-phosphate 4-epimerase	-0.0497	0.2148
JW3557	*yiaU*	Putative DNA-binding transcriptional regulator	-0.0081	0.8394
JW3558	*yiaV*	Signal-anchored membrane fusion protein (MFP) component of efflux pump	0.0310	0.6892
JW3559	*yiaW*	DUF3302 family inner membrane protein	-0.0111	0.7257

JW3561	*aldB*	Aldehyde dehydrogenase B	0.0392	0.2789
JW3563	*selB*	Selenocysteinyl-trna-specific translation factor	0.0997	0.0384
JW3564	*selA*	Selenocysteine synthase	0.0192	0.1173
JW3565	*yibF*	Glutathione S-transferase homolog	-0.0286	0.4798
JW3566	*rhsA*	Rhs protein with putative toxin 55 domain; putative polysaccharide synthesis/export protein; putative neighboring cell growth inhibitor	-0.0031	0.9326
JW3568	*yibA*	Putative immunity protein for polymorphic toxin rhsa; HEAT-domain protein; lethality reduction protein	0.0237	0.6415
JW3570	*yibG*	TPR-like repeat protein	-0.0047	0.8991
JW3571	*yibH*	Putative membrane fusion protein (MFP) component of efflux pump	-0.0570	0.0877
JW3572	*yibI*	DUF3302 family inner membrane protein	-0.0366	0.3645
JW3573	*mtlA*	Mannitol-specific PTS enzyme: IIA, IIB and IIC components	-0.0813	0.0707
JW3574	*mtlD*	Mannitol-1-phosphate dehydrogenase, NAD-dependent	-0.0640	0.1163
JW3575	*mtlR*	Mannitol operon repressor	0.0834	0.0035
JW3576	*yibT*	Uncharacterized protein	0.0445	0.2938
JW3577	*yibL*	Ribosome-associated DUF2810 family protein	0.0148	0.6692
JW3578	*lldP*	L-lactate permease	0.0677	0.1802
JW3579	*lldR*	Dual role activator/repressor for lldprd operon	-0.0068	0.8286
JW3580	*lldD*	L-lactate dehydrogenase, FMN-linked	-0.1387	0.0772
JW3581	*yibK*	Trna Leu mc34,mu34 2'-O-methyltransferase, SAM-dependent	0.0924	0.0366
JW3582	*cysE*	Serine acetyltransferase	-0.0349	0.1363
JW3584	*secB*	Protein export chaperone	-0.0321	0.1263
JW3585	*grxC*	Glutaredoxin 3	0.1020	0.0129
JW3586	*yibN*	Putative rhodanese-related sulfurtransferase	-0.0041	0.9085
JW3587	*gpmI*	Phosphoglycero mutase III, cofactor-independent	0.1302	0.0062
JW3590	*yibD*	LPS(hepiii)-glucuronic acid glycosyltransferase	0.0149	0.7919
JW3591	*tdh*	L-threonine 3-dehydrogenase, NAD(P)-binding	0.1607	0.0025
JW3592	*kbl*	Glycine C-acetyltransferase	0.0501	0.1865
JW3596	*rfaC*	ADP-heptose:LPS heptosyl transferase I	0.2006	0.0000
JW3599	*rfaZ*	Lipopolysaccharide kdoiii transferase; lipopolysaccharide core biosynthesis protein	0.0517	0.0593
JW3601	*rfaJ*	Lipopolysaccharide 1,2-glucosyltransferase; UDP-glucose:(glucosyl)LPS alpha-1,2-glucosyltransferase	-0.0025	0.9609
JW3602	*rfaI*	UDP-D-galactose:(glucosyl)lipopolysaccharide-alpha-1,3-D-galactosyltransferase	-0.0232	0.2438
JW3603	*rfaB*	Lipopolysaccharide 1,6-galactosyltransferase; UDP-D-galactose:(glucosyl)lipopolysaccharide-1,6-D-galactosyltransferase	-0.0183	0.4291
JW3604	*rfaS*	Lipopolysaccharide rhamnose:kdoiii transferase; lipopolysaccharide core biosynthesis protein	-0.0072	0.8461

JW3610	*mutM*	Formamidopyrimidine/5-formyluracil/ 5-hydroxymethyluracil DNA glycosylase	-0.0049	0.8940
JW3611	*rpmG*	50S ribosomal subunit protein L33	0.0209	0.6020
JW3617	*pyrE*	Orotate phosphoribosyltransferase	-0.0167	0.7466
JW3618	*rph*	Ribonuclease PH (defective)	-0.0467	0.2720
JW3619	*yicC*	UPF0701 family protein	-0.0871	0.0405
JW3620	*dinD*	DNA damage-inducible protein	0.0239	0.3924
JW3621	*yicG*	UPF0126 family inner membrane protein	-0.0092	0.8309
JW3622	*ligB*	DNA ligase, NAD(+)-dependent	-0.0001	0.9955
JW3624	*rpoZ*	RNA polymerase, omega subunit	-0.1039	0.0047
JW3626	*trmH*	Trna mg18-2'-O-methyltransferase, SAM-dependent	-0.0267	0.4914
JW3627	*recG*	ATP-dependent DNA helicase	-0.0017	0.9618
JW3628	*gltS*	Glutamate transporter	0.0393	0.3754
JW3629	*yicE*	Xanthine permease	0.1217	0.0561
JW3630	*yicH*	Putative inner membrane-anchored periplasmic asma family protein	-0.0092	0.7860
JW3631	*yicI*	Putative alpha-glucosidase	0.0611	0.0076
JW3633	*setC*	Putative arabinose efflux transporter	0.0162	0.7136
JW3634	*yicL*	Eama family inner membrane putative transporter	-0.0068	0.7636
JW3635	*nlpA*	Cytoplasmic membrane lipoprotein-28	0.0469	0.1388
JW3641	*uhpT*	Hexose phosphate transporter	0.0389	0.3091
JW3642	*uhpC*	Membrane protein regulates uhpt expression	0.0214	0.4066
JW3643	*uhpB*	Sensory histidine kinase in two-component regulatory sytem with uhpa	0.0146	0.6425
JW3644	*uhpA*	Response regulator in two-component regulatory system wtih uhpb	0.0383	0.4334
JW3645	*ilvN*	Acetolactate synthase 1 small subunit	0.0302	0.3962
JW3646	*ilvB*	Acetolactate synthase 2 large subunit	0.1549	0.0068
JW3647	*ivbL*	Ilvb operon leader peptide	-0.0244	0.6146
JW3650	*yidF*	Putative Cys-type oxidative yidj-maturating enzyme	0.0285	0.1511
JW3651	*yidG*	Inner membrane protein	-0.0034	0.9248
JW3652	*yidH*	DUF202 family inner membrane protein	-0.0037	0.9000
JW3653	*yidI*	Inner membrane protein	0.0081	0.7891
JW3654	*yidJ*	Sulfatase/phosphatase superfamily protein	0.0575	0.1523
JW3656	*yidL*	Arac family putative transcriptional regulator	-0.0034	0.8777
JW3658	*glvG*	CP4-44 prophage; putative disrupted hemin or colicin receptor	0.0474	0.1081
JW3659	*glvB*	Pseudogene, arbutin specific enzyme IIC component of PTS	-0.0537	0.1641
JW3660	*glvC*	Putative permease IIC component glvc	0.0335	0.2694
JW3661	*yidP*	UTRA domain-containing gntr family putative transcriptional regulator	0.0139	0.6474
JW3662	*yidE*	Putative transporter	0.0108	0.8294
JW3663	*ibpB*	Heat shock chaperone	-0.0337	0.3791

JW3664	*ibpA*	Heat shock chaperone	-0.0573	0.2489
JW3670	*dgoK*	2-oxo-3-deoxygalactonate kinase	0.0182	0.6966
JW3675	*yidB*	DUF937 family protein	-0.0473	0.0889
JW3677	*recF*	Gap repair protein	-0.0922	0.0000
JW3682	*yidD*	Membrane protein insertion efficiency factor, UPF0161 family inner membrane protein	-0.0402	0.0750
JW3684	*trmE*	Trna U34 5-methylaminomethyl-2-thiouridine modification gtpase	-0.0495	0.1445
JW3685	*tnaC*	Tryptophanase leader peptide	-0.0028	0.9295
JW3686	*tnaA*	Tryptophanase/L-cysteine desulfhydrase, PLP-dependent	-0.0219	0.5292
JW3688	*mdtL*	Multidrug efflux system protein	0.0231	0.4654
JW3689	*yidZ*	Putative DNA-binding transcriptional regulator	-0.0455	0.3723
JW3690	*yieE*	Phosphopantetheinyl transferase superfamily protein	-0.0446	0.1952
JW3691	*yieF*	Chromate reductase, Class I, flavoprotein	-0.0590	0.1701
JW3692	*yieG*	Adenine permease, high affinity; adenine:H+ symporter	0.0475	0.1973
JW3693	*yieH*	Phosphoenolpyruvate and 6-phosphogluconate phosphatase	0.1707	0.0187
JW3694	*yieI*	PRK09823 family inner membrane protein, crebc regulon	-0.0608	0.1569
JW3698	*bglH*	Carbohydrate-specific outer membrane porin, cryptic	-0.0325	0.3908
JW3699	*bglB*	Cryptic phospho-beta-glucosidase B	0.0637	0.1191
JW3700	*bglF*	Fused beta-glucoside-specific PTS enzymes: IIA component/IIB component/IIC component	0.0262	0.4161
JW3702	*phoU*	Negative regulator of phor/phob two-component regulator	-0.0712	0.0509
JW3703	*pstB*	Phosphate ABC transporter atpase	0.1196	0.0035
JW3704	*pstA*	Phosphate ABC transporter permease	0.0832	0.0195
JW3705	*pstC*	Phosphate ABC transporter permease	-0.0760	0.0833
JW3706	*pstS*	Phosphate ABC transporter periplasmic binding protein	0.0452	0.2963
JW3709	*atpC*	F1 sector of membrane-bound ATP synthase, epsilon subunit	-0.0768	0.1486
JW3710	*atpD*	F1 sector of membrane-bound ATP synthase, beta subunit	-0.0515	0.0688
JW3711	*atpG*	F1 sector of membrane-bound ATP synthase, gamma subunit	0.0114	0.7050
JW3712	*atpA*	F1 sector of membrane-bound ATP synthase, alpha subunit	-0.0791	0.1252
JW3713	*atpH*	F1 sector of membrane-bound ATP synthase, delta subunit	0.1756	0.0004
JW3714	*atpF*	F0 sector of membrane-bound ATP synthase, subunit b	-0.0422	0.3118
JW3715	*atpE*	F0 sector of membrane-bound ATP synthase, subunit c	0.1721	0.0067
JW3716	*atpB*	F0 sector of membrane-bound ATP synthase, subunit a	0.0744	0.0012

JW3718	*gidB*	16S rrna m(7)G527 methyltransferase, SAM-dependent; glucose-inhibited cell-division protein	-0.0333	0.5285
JW3719	*gidA*	5-methylaminomethyl-2-thiouridine modification at trna U34	0.0358	0.2999
JW3720	*mioC*	FMN-binding protein mioc	-0.0710	0.0825
JW3721	*asnC*	Transcriptional activator of asna; autorepressor	-0.0489	0.3924
JW3725	*yieN*	Hexameric AAA+ moxr family atpase, putative molecular chaperone	-0.0055	0.8549
JW3728	*rbsA*	D-ribose ABC transporter atpase	0.0889	0.0617
JW3729	*rbsC*	D-ribose ABC transporter permease	0.0595	0.0514
JW3730	*rbsB*	D-ribose ABC transporter periplasmic binding protein; ribose chemotaxis receptor	0.0156	0.5165
JW3731	*rbsK*	Ribokinase	0.0717	0.0147
JW3732	*rbsR*	Transcriptional repressor of ribose metabolism	-0.0272	0.3559
JW3733	*hsrA*	Putative multidrug or homocysteine efflux system	0.0496	0.2035
JW3737	*yifE*	UPF0438 family protein	-0.0067	0.8457
JW3738	*yifB*	Magnesium chelatase family protein and putative transcriptional regulator	0.0214	0.6396
JW3739	*ilvL*	Ilvg operon leader peptide	0.0871	0.0608
JW3740	*ilvG*	CP4-44 prophage; putative disrupted hemin or colicin receptor	0.0568	0.2308
JW3741	*ilvG*	CP4-44 prophage; putative disrupted hemin or colicin receptor	-0.0837	0.0315
JW3745	*ilvA*	L-threonine dehydratase, biosynthetic; also known as threonine deaminase	0.0638	0.1947
JW3746	*ilvY*	Transcriptional activator of ilvc; autorepressor	0.2151	0.0008
JW3747	*ilvC*	Ketol-acid reductoisomerase, NAD(P)-binding	-0.0552	0.0399
JW3748	*ppiC*	Peptidyl-prolyl cis-trans isomerase C (rotamase C)	0.1401	0.0051
JW3749	*yifO*	Pemk toxin family pseudogene	0.0670	0.0703
JW3750	*yifN*	CP4-44 prophage; putative disrupted hemin or colicin receptor	-0.0547	0.0435
JW3753	*rhlB*	ATP-dependent RNA helicase	-0.0462	0.4034
JW3755	*rhoL*	Putative rho operon leader peptide	-0.0289	0.5327
JW3756	*rho*	Transcription termination factor	-0.0172	0.7728
JW3758	*rfe*	UDP-glcnac:undecaprenylphosphate glcnac-1-phosphate transferase	0.1181	0.0577
JW3765	*rffA*	Dtdp-4-amino-4,6-dideoxygalactose transaminase	0.0458	0.2098
JW3766	*wzxE*	O-antigen translocase	0.0223	0.6144
JW3774	*hemY*	Putative protoheme IX synthesis protein	0.0287	0.2985
JW3775	*hemX*	Putative uroporphyrinogen III methyltransferase	-0.0124	0.8001
JW3778	*cyaA*	Adenylate cyclase	0.0309	0.2885
JW3779	*cyaY*	Iron-dependent inhibitor of iron-sulfur cluster formation; frataxin; iron-binding and oxidizing protein	-0.0124	0.6638
JW3780	*yzcX*	Uncharacterized protein	-0.0081	0.7195
JW3781	*yifL*	Putative lipoprotein	0.0372	0.0345

JW3783	*yigA*	DUF484 family protein	0.0208	0.6932
JW3784	*xerC*	Site-specific tyrosine recombinase	-0.0047	0.8756
JW3789	*corA*	Magnesium/nickel/cobalt transporter	-0.0037	0.9214
JW3794	*pldA*	Outer membrane phospholipase A	-0.0150	0.7208
JW3803	*yigM*	Putative inner membrane eama-like transporter	-0.0203	0.5049
JW3804	*metR*	Methionine biosynthesis regulon transcriptional regulator	0.3722	0.0037
JW3805	*metE*	5-methyltetrahydropteroyltriglutamate-homocysteine S-methyltransferase	0.0551	0.4043
JW3808	*udp*	Uridine phosphorylase	0.0569	0.1373
JW3809	*rmuC*	DNA recombination protein	0.1378	0.0268
JW3813	*tatA*	Tatabce protein translocation system subunit	0.0897	0.0768
JW3815	*tatC*	Tatabce protein translocation system subunit	-0.0234	0.4162
JW3818	*rfaH*	Transcription antitermination protein	0.0692	0.0451
JW3820	*fre*	NAD(P)H-flavin reductase	0.0614	0.0681
JW3822	*fadB*	Fused 3-hydroxybutyryl-coa epimerase/delta(3)-cis-delta(2)-trans-enoyl-coa isomerase/enoyl-coa hydratase/3-hydroxyacyl-coa dehydrogenase	0.0519	0.0055
JW3823	*pepQ*	Proline dipeptidase	-0.1378	0.0046
JW3829	*mobA*	Molybdopterin-guanine dinucleotide synthase	0.1393	0.0011
JW3830	*yihD*	DUF1040 protein yihd	-0.0418	0.3687
JW3831	*yihE*	Cpx stress response Thr/Ser protein kinase; mazf antagonist protein	0.0622	0.0213
JW3834	*yihG*	Inner membrane protein, inner membrane acyltransferase	-0.0614	0.1657
JW3835	*polA*	5' to 3' DNA polymerase and 3' to 5'/5' to 3' exonuclease	0.0850	0.0532
JW3837	*yihI*	Activator of Der gtpase	0.0768	0.0085
JW3838	*hemN*	Coproporphyrinogen III oxidase, SAM and NAD(P)H dependent, oxygen-independent	0.1067	0.0018
JW3839	*glnG*	Fused DNA-binding response regulator in two-component regulatory system with glnl: response regulator/sigma54 interaction protein	-0.0223	0.6510
JW3840	*glnL*	Sensory histidine kinase in two-component regulatory system with glng	0.0173	0.7921
JW3841	*glnA*	Glutamine synthetase	-0.0282	0.5898
JW3843	*yihL*	Putative DNA-binding transcriptional regulator	-0.0631	0.1071
JW3844	*yihM*	Putative sugar phosphate isomerase	-0.0008	0.9828
JW3848	*yihP*	Putative 2,3-dihydroxypropane-1-sulphonate exporter, membrane protein	-0.0269	0.3606
JW3849	*yihQ*	Putative sulpholipid alpha-glucosidase; alpha-glucosyl fluoride glucosidase	0.0423	0.3851
JW3850	*yihR*	Putative sulphoquinovose mutarotase	0.1159	0.0045
JW3852	*yihT*	6-deoxy-6-sulphofructose-1-phosphate aldolase	0.0276	0.4382
JW3853	*yihU*	3-sulpholactaldehyde (SLA) reductase, NADH-dependent; gamma-hydroxybutyrate dehydrogenase, NADH-dependent	0.0180	0.5926

JW3857	*rbn*	Brkb family putative transporter, inner membrane protein	0.0356	0.1535
JW3858	*dtd*	D-tyr-trna(Tyr) deacylase	-0.0223	0.7202
JW3859	*yiiD*	GNAT family putative N-acetyltransferase	-0.0823	0.0916
JW3862	*fdhE*	Formate dehydrogenase formation protein	-0.0029	0.9597
JW3863	*fdoI*	Formate dehydrogenase-O, cytochrome b556 subunit	0.0602	0.0255
JW3864	*fdoH*	Formate dehydrogenase-O, Fe-S subunit	0.0560	0.0541
JW3865	*fdoG*	Formate dehydrogenase-O, large subunit	-0.0324	0.3957
JW3866	*fdhD*	Formate dehydrogenase formation protein	-0.0230	0.6456
JW3867	*yiiG*	DUF3829 family lipoprotein	-0.0434	0.3389
JW3868	*frvR*	Putative frv operon regulator; contains a PTS EIIA domain	-0.0004	0.9911
JW3869	*frvX*	Putative peptidase	0.0218	0.4914
JW3872	*yiiL*	L-rhamnose mutarotase	0.0294	0.5575
JW3876	*rhaS*	Transcriptional activator of rhabad and rhat	0.0306	0.1960
JW3877	*rhaR*	Transcriptional activator of rhasr	0.0016	0.9681
JW3878	*rhaT*	L-rhamnose:proton symporter	0.0425	0.3272
JW3879	*sodA*	Superoxide dismutase, Mn	0.0604	0.0747
JW3882	*cpxA*	Sensory histidine kinase in two-component regulatory system with cpxr	0.0264	0.5253
JW3883	*cpxR*	Response regulator in two-component regulatory system with cpxa	-0.0734	0.0785
JW3886	*fieF*	Ferrous iron and zinc transporter	-0.0247	0.5138
JW3887	*pfkA*	6-phosphofructokinase I	0.0865	0.0520
JW3888	*sbp*	Sulfate transporter subunit	-0.0847	0.1828
JW3889	*cdh*	CDP-diacylglycerol phosphotidylhydrolase	-0.0489	0.0955
JW3890	*tpiA*	Triosephosphate isomerase	-0.0010	0.0214
JW3891	*yiiQ*	DUF1454 family putative periplasmic protein	0.0666	0.2348
JW3892	*yiiR*	DUF805 family putative inner membrane protein	0.0050	0.9033
JW3893	*yiiS*	UPF0381 family protein	-0.0064	0.9029
JW3894	*yiiT*	Stress-induced protein	-0.0330	0.3617
JW3895	*fpr*	Ferredoxin-NADP reductase; flavodoxin reductase	0.0144	0.7088
JW3896	*glpX*	Fructose 1,6-bisphosphatase II	0.0855	0.1351
JW3897	*glpK*	Glycerol kinase	-0.0464	0.1114
JW3898	*glpF*	Glycerol facilitator	0.0026	0.9570
JW3899	*yiiU*	Ftsz stabilizer, septal ring assembly factor, cell division stimulator	-0.1240	0.0111
JW3900	*rraA*	Ribonuclease E (rnase E) inhibitor protein	0.0093	0.7880
JW3901	*menA*	1,4-dihydroxy-2-naphthoate octaprenyltransferase	0.0621	0.2205
JW3902	*hslU*	Molecular chaperone and atpase component of hsluv protease	0.0197	0.5740
JW3903	*hslV*	Peptidase component of the hsluv protease	-0.0488	0.3213
JW3905	*cytR*	Anti-activator for cytr-CRP nucleoside utilization regulon	0.1105	0.0391

JW3906	*priA*	Primosome factor n' (replication factor Y)	0.0024	0.0192
JW3907	*rpmE*	50S ribosomal subunit protein L31	-0.0419	0.1750
JW3908	*yiiX*	Putative lipid binding hydrolase, DUF830 family protein	-0.0096	0.8217
JW3909	*metJ*	Transcriptional repressor, S-adenosylmethionine-binding	-0.2917	0.0767
JW3910	*metB*	Cystathionine gamma-synthase, PLP-dependent	0.0590	0.3756
JW3911	*metL*	Bifunctional aspartokinase/homoserine dehydrogenase 2	-0.1890	0.0000
JW3913	*metF*	5,10-methylenetetrahydrofolate reductase	0.0429	0.0695
JW3914	*katG*	Catalase-peroxidase HPI, heme b-containing	0.0107	0.7912
JW3916	*yijF*	DUF1287 family protein	0.0753	0.2169
JW3918	*fsaB*	Fructose-6-phosphate aldolase 2	0.0006	0.9903
JW3921	*frwC*	Putative enzyme IIC component of PTS	0.0111	0.7333
JW3922	*frwB*	Putative enzyme IIB component of PTS	-0.0228	0.6492
JW3923	*pflD*	Putative glycine radical domain-containing pyruvate formate-lyase	-0.0844	0.0200
JW3924	*pflC*	Putative [formate-C-acetyltransferase 2]-activating enzyme; pyruvate formate-lyase 1-activating enzyme	-0.0005	0.9891
JW3925	*frwD*	Putative enzyme IIB component of PTS	-0.0154	0.5589
JW3926	*yijO*	Arac family putative transcriptional activator	0.0170	0.7107
JW3927	*yijP*	LPS heptose I phosphoethanolamine transferase	-0.1918	0.1174
JW3928	*ppc*	Phosphoenolpyruvate carboxylase	-0.1489	0.1398
JW3929	*argE*	Acetylornithine deacetylase	0.0368	0.4426
JW3930	*argC*	N-acetyl-gamma-glutamylphosphate reductase, NAD(P)-binding	0.0755	0.0241
JW3932	*argH*	Argininosuccinate lyase	0.0287	0.5398
JW3933	*oxyR*	Oxidative and nitrosative stress transcriptional regulator	-0.0196	0.6120
JW3935	*fabR*	Transcriptional repressor of faba and fabb	-0.0014	0.9729
JW3937	*trmA*	Trna m(5)U54 methyltransferase, SAM-dependent; tmrna m(5)U341 methyltransferase	-0.0534	0.0810
JW3938	*btuB*	Vitamin B12/cobalamin outer membrane transporter	0.0093	0.7752
JW3942	*coaA*	Pantothenate kinase	0.1930	0.0021
JW3943	*tufB*	Translation elongation factor EF-Tu 2	0.0003	0.9951
JW3946	*rplK*	50S ribosomal subunit protein L11	-0.0039	0.8965
JW3947	*rplA*	50S ribosomal subunit protein L1	0.0558	0.1973
JW3952	*htrC*	Stationary phase growth adaptation protein	-0.0871	0.0118
JW3953	*thiH*	Tyrosine lyase, involved in thiamine-thiazole moiety synthesis	0.0221	0.6826
JW3955	*thiS*	Immediate sulfur donor in thiazole formation	0.1271	0.0042
JW3956	*thiF*	Adenylyltransferase, modifies this C-terminus	0.0363	0.2425
JW3957	*thiE*	Thiamine phosphate synthase (thiamine phosphate pyrophosphorylase)	0.2258	0.0004
JW3958	*thiC*	Phosphomethylpyrimidine synthase	-0.0222	0.6732

JW3959	*rsd*	Stationary phase protein, binds sigma 70 RNA polymerase subunit	-0.0551	0.3845
JW3961	*hemE*	Uroporphyrinogen decarboxylase	0.0678	0.0467
JW3963	*yjaG*	DUF416 domain protein	-0.0214	0.5626
JW3964	*hupA*	HU, DNA-binding transcriptional regulator, alpha subunit	0.0542	0.2712
JW3965	*yjaH*	DUF1481 family putative lipoprotein	-0.0254	0.5794
JW3967	*zraS*	Sensory histidine kinase in two-component regulatory system with zrar	0.0900	0.0494
JW3968	*zraR*	Fused DNA-binding response regulator in two-component regulatory system with zras: response regulator/sigma54 interaction protein	0.0858	0.1120
JW3969	*purD*	Phosphoribosylglycinamide synthetase phosphoribosylamine-glycine ligase	0.0263	0.7158
JW3970	*purH*	IMP cyclohydrolase and phosphoribosylaminoimidazolecarboxamide formyltransferase	-0.0846	0.0032
JW3971	*yjaA*	Stress-induced protein	0.0436	0.3152
JW3973	*metA*	Homoserine O-transsuccinylase	0.2412	0.0018
JW3974	*aceB*	Malate synthase A	0.0159	0.7386
JW3975	*aceA*	Isocitrate lyase	0.0144	0.5314
JW3976	*aceK*	Isocitrate dehydrogenase kinase/phosphatase	-0.0955	0.0398
JW3977	*arpA*	Ankyrin repeat protein	0.0129	0.5985
JW3978	*iclR*	Transcriptional repressor	-0.0252	0.2830
JW3979	*metH*	Homocysteine-N5-methyltetrahydrofolate transmethylase, B12-dependent	-0.1545	0.0546
JW3980	*yjbB*	Putative Na+/Pi-cotransporter	0.0046	0.8420
JW3981	*pepE*	Peptidase E, alpha-aspartyl dipeptidase	-0.0618	0.0465
JW3982	*yjbC*	23S rrna pseudouridine(2604) synthase	0.0836	0.0011
JW3983	*yjbD*	DUF3811 family protein	-0.0077	0.8626
JW3984	*lysC*	Lysine-sensitive aspartokinase 3	-0.0609	0.6105
JW3985	*pgi*	Glucosephosphate isomerase	0.1018	0.1363
JW3988	*yjbG*	Extracellular polysaccharide export OMA protein	-0.0406	0.2935
JW3989	*yjbH*	DUF940 family extracellular polysaccharide protein	-0.0680	0.2311
JW3990	*yjbA*	Phosphate starvation inducible protein	-0.0221	0.6336
JW3991	*xylE*	D-xylose transporter	-0.0087	0.8844
JW3992	*malG*	Maltose transporter subunit	0.0332	0.3064
JW3993	*malF*	Maltose transporter subunit	0.0369	0.3827
JW3994	*malE*	Maltose transporter subunit	0.0679	0.0203
JW3995	*malK*	Maltose ABC transportor atpase	-0.0030	0.9274
JW3996	*lamB*	Maltose outer membrane porin (maltoporin)	0.0165	0.6835
JW3997	*malM*	Maltose regulon periplasmic protein	-0.0602	0.1589
JW3998	*yjbI*	Pseudogene, sopa-related, pentapeptide repeats-containing	0.0148	0.6139
JW4002	*dgkA*	Diacylglycerol kinase	-0.0237	0.5532

JW4004	*dinF*	Oxidative stress resistance protein; putative MATE family efflux pump; UV and mitomycin C inducible protein	0.0704	0.4124
JW4005	*yjbJ*	Stress-induced protein, UPF0337 family	0.0100	0.8211
JW4007	*yjbL*	Uncharacterized protein	-0.0253	0.4180
JW4008	*yjbM*	Uncharacterized protein	0.0157	0.6606
JW4011	*qor*	Quinone oxidoreductase, NADPH-dependent	0.0837	0.1182
JW4013	*alr*	Alanine racemase, biosynthetic, PLP-binding	0.3526	0.0036
JW4014	*tyrB*	Tyrosine aminotransferase, tyrosine-repressible, PLP-dependent	0.0247	0.7122
JW4015	*aphA*	Acid phosphatase/phosphotransferase, class B, non-specific	-0.0569	0.0724
JW4018	*yjbR*	DUF419 family protein	0.0143	0.7992
JW4019	*uvrA*	Atpase and DNA damage recognition protein of nucleotide excision repair excinuclease uvrabc	-0.0328	0.5868
JW4022	*yjcC*	Putative membrane-anchored cyclic-di-GMP phosphodiesterase	-0.0100	0.8351
JW4025	*yjcD*	Guanine/hypoxanthine permease, high affinity; guanine/hypoxanthine:H+ symporter	0.0264	0.6654
JW4026	*yjcE*	Putative cation/proton antiporter	0.0001	0.8435
JW4027	*yjcF*	Uncharacterized protein	0.0886	0.0035
JW4028	*actP*	Acetate transporter	-0.0538	0.1443
JW4029	*yjcH*	DUF485 family inner membrane protein	0.0684	0.2898
JW4030	*acs*	Acetyl-coa synthetase	0.0377	0.3752
JW4031	*nrfA*	Nitrite reductase, formate-dependent, cytochrome	-0.2103	0.2397
JW4032	*nrfB*	Nitrite reductase, formate-dependent, penta-heme cytochrome c	0.0247	0.4743
JW4033	*nrfC*	Formate-dependent nitrite reductase, 4Fe4S subunit	-0.0353	0.5636
JW4034	*nrfD*	Formate-dependent nitrite reductase, membrane subunit	-0.0275	0.4026
JW4035	*nrfE*	Heme lyase (nrfefg) for insertion of heme into c552, subunit nrfe	-0.0372	0.4665
JW4036	*nrfF*	Heme lyase (nrfefg) for insertion of heme into c552, subunit nrff	0.0325	0.5424
JW4037	*nrfG*	Heme lyase (nrfefg) for insertion of heme into c552, subunit nrfg	0.0644	0.3680
JW4038	*gltP*	Glutamate/aspartate:proton symporter	-0.0117	0.7798
JW4039	*yjcO*	Sel1 family TPR-like repeat protein	0.0132	0.8317
JW4040	*fdhF*	Formate dehydrogenase-H, selenopolypeptide subunit	-0.0223	0.4799
JW4041	*yjcP*	Outer membrane factor of efflux pump	0.0351	0.5659
JW4042	*yjcQ*	Membrane translocase (MDR) of mdtnop efflux pump, PET family	-0.0076	0.7062
JW4043	*yjcR*	Membrane fusion protein of efflux pump	0.1060	0.0101
JW4046	*alsE*	Allulose-6-phosphate 3-epimerase	0.0178	0.7279
JW4047	*alsC*	D-allose ABC transporter permease	-0.0125	0.8543
JW4048	*alsA*	D-allose ABC transporter atpase	0.0568	0.0790

JW4049	*alsB*	D-allose ABC transporter periplasmic binding protein	0.0148	0.5440
JW4050	*rpiR*	D-allose-inducible als operon transcriptional repressor; autorepressor; repressor of rpir	0.1148	0.0206
JW4051	*rpiB*	Ribose 5-phosphate isomerase B/allose 6-phosphate isomerase	-0.0955	0.0091
JW4053	*phnP*	5-phospho-alpha-D-ribosyl 1,2-cyclic phosphate phosphodiesterase	-0.0917	0.0132
JW4054	*phnO*	Aminoalkylphosphonate N-acetyltransferase	-0.0097	0.6055
JW4055	*phnN*	Ribose 1,5-bisphosphokinase	0.1062	0.0012
JW4056	*phnM*	Ribophosphonate triphosphate hydrolase	-0.0015	0.9750
JW4057	*phnL*	Ribophosphonate triphosphate synthase subunit; putative ABC transporter-related atpase	0.0278	0.5018
JW4059	*phnJ*	Carbon-phosphorus lyase, SAM-dependent	0.0691	0.0074
JW4060	*phnI*	Ribophosphonate triphosphate synthase complex putative catalytic subunit	-0.0075	0.8927
JW4061	*phnH*	Ribophosphonate triphosphate synthase subunit	-0.0421	0.0765
JW4062	*phnG*	Ribophosphonate triphosphate synthase subunit	-0.0171	0.5450
JW4063	*phnF*	Putative DNA-binding transcriptional regulator of phosphonate uptake and biodegradation	0.1314	0.0597
JW4064	*phnE*	CP4-44 prophage; putative disrupted hemin or colicin receptor	-0.0307	0.5303
JW4065	*phnE*	Putative cryptic phosphonate transport system permease protein phne2	-0.0222	0.2908
JW4066	*phnD*	Phosphonate ABC transporter periplasmic binding protein	0.0425	0.3577
JW4067	*phnC*	Phosphonate ABC transporter atpase	0.1183	0.0362
JW4068	*phnB*	Metalloprotein superfamily protein	-0.0006	0.9866
JW4069	*phnA*	Zinc-ribbon family protein	-0.0543	0.1487
JW4072	*proP*	Proline/glycine betaine transporter	-0.0240	0.5385
JW4074	*basR*	Response regulator in two-component regulatory system with bass	-0.0172	0.7861
JW4076	*adiC*	Arginine:agmatine antiporter	0.1086	0.0005
JW4077	*adiY*	Adi system transcriptional activator	0.1247	0.0200
JW4079	*melR*	Melibiose operon transcriptional regulator; autoregulator	-0.0246	0.4962
JW4080	*melA*	Alpha-galactosidase, NAD(P)-binding	0.0494	0.2766
JW4081	*melB*	Melibiose:sodium symporter	0.0514	0.0655
JW4082	*yjdF*	DUF2238 family inner membrane protein	0.0016	0.9620
JW4083	*fumB*	Anaerobic class I fumarate hydratase (fumarase B)	-0.0459	0.1239
JW4084	*dcuB*	C4-dicarboxylate transporter, anaerobic; dcus co-sensor	0.0085	0.7760
JW4085	*dcuR*	Response regulator in two-component regulatory system with dcus	-0.0210	0.5054
JW4087	*yjdI*	Putative 4Fe-4S mono-cluster protein	-0.0310	0.6867
JW4088	*yjdJ*	GNAT family putative N-acetyltransferase	0.0014	0.9695
JW4089	*yjdK*	Antitoxin of ghots toxin-antitoxin pair; endonuclease for ghot mrna	-0.0203	0.5644

JW4090	*lysU*	Lysine trna synthetase, inducible	0.0607	0.2346
JW4091	*yjdL*	Dipeptide and tripeptide permease	0.0023	0.9279
JW4092	*cadA*	Lysine decarboxylase, acid-inducible	-0.0843	0.0249
JW4093	*cadB*	Putative lysine/cadaverine transporter	0.0143	0.6662
JW4099	*aspA*	Aspartate ammonia-lyase	0.0708	0.0277
JW4100	*fxsA*	Suppressor of F exclusion of phage T7	0.0564	0.2158
JW4101	*yjeH*	L-methionine and branched chain amino acid exporter	-0.0352	0.5410
JW4103	*groL*	Cpn60 chaperonin groel, large subunit of groesl	-0.0603	0.1908
JW4106	*yjeK*	EF-P-Lys34 lysylation protein; weak lysine 2,3-aminomutase	-0.0096	0.6327
JW4107	*efp*	Polyproline-specific translation elongation factor EF-P	0.0115	0.6735
JW4108	*ecnB*	Entericidin B membrane lipoprotein	0.0695	0.0624
JW4110	*blc*	Outer membrane lipoprotein cell division and growth lipocalin	0.0301	0.3562
JW4111	*ampC*	Penicillin-binding protein; beta-lactamase, intrinsically weak	0.0247	0.3269
JW4112	*frdD*	Fumarate reductase (anaerobic), membrane anchor subunit	-0.0763	0.0154
JW4113	*frdC*	Fumarate reductase (anaerobic), membrane anchor subunit	0.0415	0.4293
JW4114	*frdB*	Fumarate reductase (anaerobic), Fe-S subunit	0.0636	0.1797
JW4115	*frdA*	Anaerobic fumarate reductase catalytic and NAD/flavoprotein subunit	0.0028	0.9229
JW4116	*poxA*	Elongation Factor P Lys34 lysyltransferase	0.0434	0.3041
JW4118	*yjeN*	Uncharacterized protein	0.0211	0.7173
JW4119	*yjeO*	Inner membrane protein	-0.0210	0.7072
JW4120	*yjeP*	Mechanosensitive channel protein, miniconductance	-0.0126	0.8491
JW4122	*rsgA*	Ribosome small subunit-dependent gtpase A	0.1184	0.0698
JW4124	*yjeS*	Epoxyqueuosine reductase, cobalamine-stimulated; queosine biosynthesis	-0.0328	0.3983
JW4125	*yjeF*	Bifunctional NAD(P)H-hydrate repair enzyme; C-terminal domain ADP-dependent (S)-NAD(P)H-hydrate dehydratase and N-terminal domain NAD(P)H-hydrate epimerase	0.0044	0.9351
JW4127	*amiB*	N-acetylmuramoyl-l-alanine amidase II	-0.0187	0.4711
JW4128	*mutL*	Methyl-directed mismatch repair protein	-0.0397	0.4566
JW4129	*miaA*	Delta(2)-isopentenylpyrophosphate trna-adenosine transferase	-0.0666	0.1329
JW4130	*hfq*	Global srna chaperone; HF-I, host factor for RNA phage Q beta replication	0.0320	0.3729
JW4131	*hflX*	Gtpase, stimulated by 50S subunit binding; Mn(2+) homeostasis regulator	0.0872	0.0161
JW4132	*hflK*	Modulator for hflb protease specific for phage lambda cii repressor	0.0546	0.1898
JW4133	*hflC*	Hflb protease modulator specific for phage lambda cii repressor	0.0232	0.5568

JW4134	*yjeT*	DUF2065 family protein	-0.0889	0.0158
JW4135	*purA*	Adenylosuccinate synthetase	0.0271	0.4324
JW4136	*yjeB*	Nitric oxide-sensitive repressor for NO regulon	-0.0029	0.9409
JW4139	*yjfI*	DUF2170 family protein	-0.1067	0.0016
JW4140	*yjfJ*	Pspa/IM30 family protein	-0.0887	0.0721
JW4141	*yjfK*	DUF2491 family protein	0.1644	0.0153
JW4142	*yjfL*	UPF0719 family inner membrane protein	0.0281	0.5578
JW4148	*yjfP*	Acyl coa esterase	-0.0812	0.0400
JW4149	*ulaR*	Transcriptional repressor for the L-ascorbate utilization divergent operon	-0.5564	0.0001
JW4152	*ulaB*	L-ascorbate-specific enzyme IIB component of PTS	0.0475	0.3172
JW4153	*ulaC*	L-ascorbate-specific enzyme IIA component of PTS	0.0239	0.2909
JW4155	*ulaE*	L-xylulose 5-phosphate 3-epimerase	0.0224	0.6874
JW4156	*ulaF*	L-ribulose 5-phosphate 4-epimerase	0.0396	0.3022
JW4157	*yjfY*	Yhcn family protein, periplasmic	0.0060	0.9504
JW4158	*rpsF*	30S ribosomal subunit protein S6	-0.1290	0.1688
JW4159	*priB*	Primosomal protein N	0.0831	0.0431
JW4161	*rplI*	50S ribosomal subunit protein L9	0.0089	0.7678
JW4162	*yjfZ*	Uncharacterized protein	0.0779	0.0821
JW4163	*ytfA*	Pseudogene, related to transcriptional regulators	0.0083	0.8653
JW4166	*cycA*	D-alanine/D-serine/glycine transporter	-0.0178	0.7507
JW4167	*ytfE*	Iron-sulfur cluster repair protein RIC	0.0469	0.4769
JW4168	*ytfF*	DMT transporter family inner membrane protein	-0.0862	0.1403
JW4169	*ytfG*	NAD(P)H:quinone oxidoreductase	0.0263	0.6956
JW4171	*cpdB*	2':3'-cyclic-nucleotide 2'-phosphodiesterase	-0.0140	0.6210
JW4172	*cysQ*	3'(2'),5'-bisphosphate nucleotidase	-0.2027	0.0014
JW4175	*ytfJ*	Putative transcriptional regulator	-0.1269	0.0458
JW4177	*ytfL*	UPF0053 family inner membrane protein	0.0729	0.0238
JW4178	*msrA*	Methionine sulfoxide reductase A	0.0136	0.6555
JW4179	*ytfM*	Translocation and assembly module for autotransporter export, outer membrane subunit	0.1394	0.0007
JW4180	*ytfN*	Translocation and assembly module for autotransporter export, inner membrane subunit	-0.0991	0.2387
JW4181	*ytfP*	GGCT-like protein	0.0384	0.6929
JW4184	*chpB*	Toxin of the chpb-chps toxin-antitoxin system	0.0760	0.1187
JW4186	*ytfQ*	Galactofuranose ABC transporter periplasmic binding protein	-0.0380	0.1459
JW4191	*fbp*	Fructose-1,6-bisphosphatase I	-0.0554	0.0920
JW4192	*mpl*	UDP-N-acetylmuramate:L-alanyl-gamma-D-glutamyl-meso-diaminopimelate ligase	-0.0345	0.3077
JW4193	*yjgA*	Ribosome-associated UPF0307 family protein	0.0613	0.4530
JW4194	*pmbA*	Putative antibiotic peptide mccb17 maturation peptidase	0.0665	0.0662

JW4195	*cybC*	CP4-44 prophage; putative disrupted hemin or colicin receptor	-0.0438	0.3683
JW4196	*nrdG*	Anaerobic ribonucleotide reductase activating protein	-0.0416	0.0980
JW4197	*nrdD*	Anaerobic ribonucleoside-triphosphate reductase	-0.0215	0.3957
JW4198	*treC*	Trehalose-6-P hydrolase	-0.0829	0.1696
JW4199	*treB*	PTS system trehalose-specific EIIBC component	-0.0128	0.7803
JW4200	*treR*	Trehalose 6-phosphate-inducible trehalose regulon transcriptional repressor	0.0175	0.7308
JW4201	*mgtA*	Magnesium transporter	0.0105	0.7795
JW4203	*pyrI*	Aspartate carbamoyltransferase, regulatory subunit	-0.0527	0.2546
JW4204	*pyrB*	Aspartate carbamoyltransferase, catalytic subunit	0.0766	0.0393
JW4205	*pyrL*	Pyrbi operon leader peptide	-0.1417	0.1364
JW4207	*yjgI*	C-di-GMP-binding biofilm dispersal mediator protein	0.0398	0.2821
JW4208	*yjgJ*	Transcriptional repressor for divergent bdca	-0.0594	0.0795
JW4211	*argI*	Ornithine carbamoyltransferase 1	0.0011	0.9812
JW4212	*yjgD*	Protein inhibitor of rnase E	0.0865	0.3528
JW4216	*holC*	DNA polymerase III, chi subunit	-0.3270	0.0001
JW4217	*pepA*	Multifunctional aminopeptidase A: a cyteinylglycinase, transcription regulator and site-specific recombination factor	0.0014	0.9742
JW4221	*idnR*	Transcriptional repressor, 5-gluconate-binding	0.0195	0.6268
JW4222	*idnT*	L-idonate and D-gluconate transporter	0.0905	0.0566
JW4223	*idnO*	5-keto-D-gluconate-5-reductase	0.0975	0.0476
JW4224	*idnD*	L-idonate 5-dehydrogenase, NAD-binding	0.0723	0.1400
JW4225	*idnK*	D-gluconate kinase, thermosensitive	0.0030	0.9178
JW4227	*intB*	Pseudogene, integrase homology	0.0277	0.4624
JW4233	*yjgW*	Uncharacterized protein	0.0253	0.4001
JW4234	*yjgX*	CP4-44 prophage; putative disrupted hemin or colicin receptor	0.0556	0.2337
JW4236	*yjgZ*	Uncharacterized protein	-0.0098	0.7745
JW4242	*yjhE*	CP4-44 prophage; putative disrupted hemin or colicin receptor	0.0169	0.6206
JW4246	*yjhV*	Pseudogene, kple2 phage-like element	-0.0211	0.3167
JW4247	*fecE*	Ferric citrate ABC transporter atpase	-0.0196	0.4134
JW4248	*fecD*	Ferric citrate ABC transporter permease	0.0565	0.5016
JW4249	*fecC*	Ferric citrate ABC transporter permease	-0.0057	0.8088
JW4250	*fecB*	Ferric citrate ABC transporter periplasmic binding protein	0.1075	0.0082
JW4251	*fecA*	Tonb-dependent outer membrane ferric citrate transporter and signal transducer; ferric citrate extracelluar receptor; fecr-interacting protein	0.0207	0.6481
JW4252	*fecR*	Anti-sigma transmembrane signal transducer for ferric citrate transport; periplasmic feca-bound ferric citrate sensor and cytoplasmic feci ECF sigma factor activator	-0.0322	0.5328

JW4253	*fecI*	RNA polymerase sigma-19 factor, fec operon-specific; ECF sigma factor	-0.0590	0.2955
JW4259	*yjhG*	Putative dehydratase	-0.0025	0.9731
JW4261	*yjhI*	Putative DNA-binding transcriptional regulator	0.0158	0.8364
JW4262	*sgcR*	Putative DNA-binding transcriptional regulator	-0.0370	0.3649
JW4263	*sgcE*	Putative epimerase	0.0528	0.2804
JW4264	*sgcA*	Putative phosphotransferase enzyme IIA component	0.0927	0.0066
JW4265	*sgcQ*	Putative nucleoside triphosphatase	-0.0454	0.1841
JW4266	*sgcC*	Putative PTS system EIIC permease component	-0.1199	0.0001
JW4268	*yjhP*	Putative methyltransferase	-0.0197	0.4643
JW4269	*yjhQ*	GNAT family putative N-acetyltransferase	-0.0684	0.5669
JW4271	*yjhR*	Pseudogene, helicase family	0.0946	0.4626
JW4272	*yjhS*	9-O-acetyl N-acetylneuraminic acid esterase	-0.0710	0.0167
JW4275	*fimB*	Tyrosine recombinase/inversion of on/off regulator of fima	-0.0079	0.7065
JW4277	*fimA*	Major type 1 subunit fimbrin (pilin)	-0.0422	0.2085
JW4282	*fimG*	Minor component of type 1 fimbriae	0.0496	0.1810
JW4284	*gntP*	Fructuronate transporter	0.0329	0.3726
JW4285	*uxuA*	Mannonate hydrolase	0.0257	0.5108
JW4286	*uxuB*	D-mannonate oxidoreductase, NAD-dependent	0.0151	0.7546
JW4288	*yjiC*	Uncharacterized protein	0.0702	0.4036
JW4290	*yjiE*	Hypochlorite-responsive transcription factor	0.0198	0.4929
JW4291	*iadA*	Isoaspartyl dipeptidase	-0.0281	0.3756
JW4292	*yjiG*	Spmb family inner membrane protein	-0.0581	0.5756
JW4295	*yjiJ*	DUF1228 family putative inner membrane MFS superfamily transporter	-0.0431	0.1115
JW4299	*yjiN*	Zinc-type alcohol dehydrogenase-like protein	-0.0413	0.5349
JW4302	*yjiQ*	Putative inactive recombination-promoting nuclease-like protein yjiq	-0.0517	0.0435
JW4303	*yjiR*	Putative DNA-binding transcriptional regulator/putative aminotransferase	-0.0351	0.6231
JW4304	*yjiS*	DUF1127 family protein	-0.0342	0.7665
JW4310	*yjiW*	Toxic peptide regulated by antisense srna symr	0.1773	0.0003
JW4311	*hsdS*	Specificity determinant for hsdm and hsdr	0.1001	0.0547
JW4313	*hsdR*	Endonuclease R Type I restriction enzyme	-0.0032	0.9462
JW4314	*mrr*	Methylated adenine and cytosine restriction protein	0.2095	0.0407
JW4316	*yjiX*	DUF466 family protein	-0.0523	0.5012
JW4318	*tsr*	Methyl-accepting chemotaxis protein I, serine sensor receptor	-0.0055	0.8884
JW4319	*yjiZ*	Putative L-galactonate:H+ symporter	-0.1432	0.0011
JW4326	*dnaT*	DNA biosynthesis protein (primosomal protein I)	-0.1285	0.0015
JW4327	*yjjB*	DUF3815 family inner membrane protein	0.0271	0.4732
JW4329	*yjjQ*	Putative transcriptional regulator	0.0177	0.6966

JW4331	*fhuF*	Ferric iron reductase involved in ferric hydroximate transport	0.0656	0.0337
JW4333	*rsmC*	16S rrna m(2)G1207 methyltransferase, SAM-dependent	-0.0712	0.1174
JW4334	*holD*	DNA polymerase III, psi subunit	-0.2171	0.0005
JW4335	*rimI*	Ribosomal-protein-S18-alanine N-acetyltransferase	-0.0013	0.9820
JW4336	*yjjG*	Dump phosphatase	0.0729	0.2052
JW4338	*osmY*	Salt-inducible putative ABC transporter periplasmic binding protein	0.0146	0.7736
JW4340	*yjjU*	Putative patatin-like family phospholipase	-0.0178	0.7077
JW4341	*yjjV*	Putative dnase	0.0066	0.7905
JW4342	*yjjW*	Putative pyruvate formate lyase activating enzyme	0.0755	0.2093
JW4343	*yjjI*	DUF3029 family protein, putative glycine radical enzyme	0.1307	0.0825
JW4344	*deoC*	2-deoxyribose-5-phosphate aldolase, NAD(P)-linked	0.0194	0.7051
JW4345	*deoA*	Thymidine phosphorylase	-0.0038	0.9364
JW4346	*deoB*	Phosphopentomutase	0.0047	0.9000
JW4347	*deoD*	Purine nucleoside phosphorylase 1; nicotinamide 1-beta-D-riboside phosphorylase	-0.0486	0.0438
JW4348	*yjjJ*	Putative protein kinase	0.0253	0.5740
JW4350	*ytjB*	SMP_2 family putative membrane-anchored periplasmic protein	-0.0456	0.3212
JW4351	*serB*	3-phosphoserine phosphatase	-0.0173	0.3861
JW4352	*radA*	DNA repair protein	-0.0112	0.6225
JW4354	*yjjK*	Energy-dependent translational throttle A	0.1213	0.0351
JW4355	*slt*	Lytic murein transglycosylase, soluble	0.0519	0.3167
JW4356	*trpR*	Transcriptional repressor, tryptophan-binding	-0.0551	0.0716
JW4358	*ytjC*	Phosphatase	-0.0224	0.6429
JW4359	*rob*	Right oric-binding transcriptional activator, arac family	0.0843	0.1541
JW4360	*creA*	Putative periplasmic protein	0.0126	0.7339
JW4361	*creB*	Response regulator in two-component regulatory system with crec	0.0322	0.4030
JW4362	*creC*	Sensory histidine kinase in two-component regulatory system with creb or phob	0.0561	0.1769
JW4363	*creD*	Inner membrane protein cred	0.0279	0.4895
JW4364	*arcA*	Response regulator in two-component regulatory system with arcb or cpxa	-0.0385	0.3720
JW4365	*yjjY*	Uncharacterized protein	0.0675	0.5230
JW4366	*yjtD*	Putative methyltransferase	-0.0263	0.4566
JW4367	*thrL*	Thr operon leader peptide	-0.0810	0.1402
JW5001	*htgA*	Uncharacterized protein	-0.0619	0.0603
JW5002	*hokC*	Toxic protein hokc	-0.1542	0.0130
JW5003	*yaaY*	Uncharacterized protein	-0.0545	0.1205
JW5004	*caiE*	Stimulator of caid and caib enzyme activities	0.1146	0.0562

JW5005	*yabI*	Ionizing radiation survival protein; deda family inner membrane protein	-0.0294	0.2398
JW5008	*yacG*	DNA gyrase inhibitor	-0.0674	0.1209
JW5009	*hpt*	Hypoxanthine phosphoribosyltransferase	0.0518	0.2142
JW5010	*yadD*	Recombination-promoting nuclease rpnc	0.0067	0.7975
JW5011	*ligT*	2'-5' RNA ligase	0.0976	0.0798
JW5012	*clcA*	H(+)/Cl(-) exchange transporter	0.0455	0.0424
JW5013	*cdaR*	Carbohydrate diacid regulon transcriptional regulator; autoregulator	0.0156	0.6813
JW5014	*yaeI*	Phosphodiesterase with model substrate bis-pnpp	0.0890	0.0359
JW5016	*yaeF*	Putative lipoprotein	-0.0359	0.2365
JW5017	*yafD*	Endo/exonuclease/phosphatase family protein	-0.0304	0.2410
JW5018	*mltD*	Putative membrane-bound lytic murein transglycosylase D	-0.0186	0.6219
JW5019	*yafV*	Putative NAD(P)-binding C-N hydrolase family amidase	0.0049	0.8894
JW5020	*fadE*	Acyl coenzyme A dehydrogenase	0.0170	0.7242
JW5022	*yafX*	CP4-6 prophage; uncharacterized protein	0.0516	0.0981
JW5023	*ykfF*	CP4-6 prophage; uncharacterized protein	0.0908	0.0567
JW5027	*mmuP*	CP4-6 prophage; putative S-methylmethionine transporter	0.0424	0.1451
JW5030	*yagV*	ECP production pilus chaperone	-0.0275	0.5715
JW5031	*ykgK*	Putative transcriptional regulator for the ecp operon	0.1277	0.0079
JW5033	*ykgL*	Uncharacterized protein	-0.0046	0.8886
JW5034	*ykgM*	Rpmj-like protein	0.0232	0.6510
JW5035	*ykgM*	50S ribosomal protein L31 type B; alternative zinc-limitation L31 protein	0.0107	0.7807
JW5037	*ykgA*	Pseudogene, arac family	-0.0141	0.7027
JW5038	*ykgB*	Reactive chlorine species (RCS) stress resistance inner membrane protein	-0.0707	0.0407
JW5039	*ykgI*	Reactive chlorine species (RCS) stress resistance periplasmic protein	-0.0062	0.8757
JW5040	*ykgC*	Reactive chlorine stress species (RCS) resistance protein; pyridine nucleotide-dependent disulfide oxidoreductase family	0.0531	0.2766
JW5042	*ykgG*	Lutc family protein; putative electron transport chain ykgefg component	0.0989	0.0391
JW5044	*yahM*	Uncharacterized protein	0.1198	0.0002
JW5046	*mhpT*	3-hydroxyphenylpropionic transporter	-0.0629	0.2419
JW5049	*ykiB*	CP4-44 prophage; putative disrupted hemin or colicin receptor	-0.0332	0.3925
JW5051	*yaiU*	CP4-44 prophage; putative disrupted hemin or colicin receptor	0.0548	0.1768
JW5052	*ampH*	D-alanyl-D-alanine-carboxypeptidase/endopeptidase; penicillin-binding protein; weak beta-lactamase	0.0986	0.0001
JW5053	*yaiZ*	DUF2754 family putative inner membrane protein	0.0947	0.0756

JW5054	*psiF*	Psif family protein	0.0325	0.1764
JW5055	*proY*	Proline-specific permease	0.1321	0.0011
JW5056	*yajI*	Putative lipoprotein	-0.0015	0.9575
JW5057	*yajL*	Oxidative-stress-resistance chaperone	0.0038	0.9181
JW5058	*yajQ*	Phage Phi6 host factor, ATP/GTP binding protein	0.0703	0.0544
JW5059	*yajR*	Putative transporter	0.0247	0.4571
JW5060	*bolA*	Stationary-phase morphogene, transcriptional repressor for mreb; also regulator for daca, dacc, and ampc	0.1500	0.0001
JW5061	*mdlB*	Putative multidrug ABC transporter atpase	0.0163	0.5122
JW5062	*ylaB*	Putative membrane-anchored cyclic-di-GMP phosphodiesterase	-0.0819	0.0249
JW5063	*ylaC*	DUF1449 family inner membrane protein	0.0251	0.5318
JW5065	*ybbJ*	Inner membrane protein; stimulator of the qmca suppressor of ftsh-htpx	0.0378	0.1561
JW5066	*ybbM*	Iron export ABC transporter permease; peroxide resistance protein	-0.0694	0.0098
JW5067	*ybbN*	Dnak co-chaperone, thioredoxin-like protein	-0.0358	0.1080
JW5070	*ybcJ*	Ribosome-associated protein; putative RNA-binding protein	0.0446	0.0871
JW5071	*sfmH*	Fima homolog, function unknown	0.0142	0.5351
JW5072	*sfmF*	Fima homolog, function unknown	-0.0095	0.8288
JW5073	*fimZ*	Response regulator family protein	0.0268	0.5384
JW5076	*ylcG*	Uncharacterized protein, DLP12 prophage	-0.0237	0.4750
JW5078	*nmpC*	CP4-44 prophage; putative disrupted hemin or colicin receptor	0.0139	0.7200
JW5079	*rzpD*	DLP12 prophage; putative murein endopeptidase	-0.0039	0.9198
JW5080	*rzoD*	DLP12 prophage; putative lipoprotein	-0.0788	0.1552
JW5081	*ybcV*	DLP12 prophage; DUF1398 family protein	-0.0286	0.1646
JW5082	*cusS*	Copper-sensing histidine kinase in two-component regulatory system with cusr	-0.0069	0.8546
JW5083	*ybdF*	DUF419 family protein	0.0571	0.1218
JW5084	*hokE*	Toxic polypeptide, small	0.0845	0.2064
JW5087	*citF*	Citrate lyase, citrate-ACP transferase (alpha) subunit	0.0228	0.5271
JW5089	*lipB*	Octanoyltransferase; octanoyl-[ACP]:protein N-octanoyltransferase	-0.0779	0.0035
JW5090	*ybeB*	Ribosomal silencing factor	0.0260	0.4872
JW5091	*ybeQ*	Sel1 family TPR-like repeat protein	-0.0140	0.7254
JW5092	*gltI*	Glutamate/aspartate periplasmic binding protein	-0.0486	0.0430
JW5094	*ybfG*	CP4-44 prophage; putative disrupted hemin or colicin receptor	-0.0110	0.7151
JW5095	*ybfH*	CP4-44 prophage; putative disrupted hemin or colicin receptor	0.0373	0.1318
JW5096	*kdpE*	Response regulator in two-component regulatory system with kdpd	0.0038	0.9418
JW5097	*abrB*	Regulator of aidb expression; inner membrane protein	-0.0393	0.0715

JW5098	*ybgO*	Putative fimbrial protein	-0.1626	0.0004
JW5099	*ybgQ*	Putative outer membrane protein	-0.1220	0.0002
JW5100	*tolB*	Periplasmic protein	0.1996	0.0004
JW5102	*ybhT*	Acrab-tolc efflux pump accessory protein, membrane-associated	-0.0394	0.1086
JW5103	*ybhJ*	Aconitase family protein	-0.0143	0.5059
JW5104	*ybhF*	Putative ABC transporter atpase	0.0228	0.5374
JW5105	*ybiX*	Fe(II)-dependent oxygenase superfamily protein	0.0169	0.6451
JW5106	*ybiM*	Colanic acid mucoidy stimulation protein	-0.0145	0.6421
JW5107	*ybiN*	23S rrna m(6)A1618 methyltransferase, SAM-dependent	-0.0282	0.4683
JW5108	*ybiO*	Mechanosensitive channel protein, intermediate conductance	-0.0126	0.5769
JW5109	*fsaA*	Fructose-6-phosphate aldolase 1	-0.0047	0.8625
JW5111	*yliB*	Glutathione-binding protein gsib	-0.0124	0.6544
JW5112	*ybjG*	Undecaprenyl pyrophosphate phosphatase	0.0469	0.3873
JW5113	*ybjI*	5-amino-6-(5-phospho-D-ribitylamino)uracil phosphatase; pyrimidine phosphatase; riboflavin synthesis	0.0196	0.4174
JW5114	*ybjK*	Transcriptional regulator of csgd and ybiji; autoregulator	-0.0778	0.0115
JW5116	*ybjT*	Putative NAD-dependent oxidoreductase	0.0460	0.0617
JW5117	*hcr*	HCP oxidoreductase, NADH-dependent	0.0835	0.1558
JW5118	*dmsA*	Dimethyl sulfoxide reductase, anaerobic, subunit A	-0.0182	0.7006
JW5119	*ycaM*	Putative transporter	0.1335	0.0126
JW5120	*ycaI*	Comec family inner membrane protein	-0.0598	0.1835
JW5121	*ssuC*	Aliphatic sulfonate ABC transporter permease	0.0332	0.4931
JW5122	*ycbQ*	Laminin-binding fimbrin subunit	-0.0656	0.1154
JW5123	*ycbV*	Putative fimbrial-like adhesin protein	0.0497	0.0699
JW5124	*ycbF*	Putative periplasmic pilin chaperone	-0.0157	0.5654
JW5125	*ycbW*	Ftsz stabilizer	0.0612	0.0050
JW5126	*ycbX*	6-N-hydroxylaminopurine detoxification oxidoreductase	-0.0356	0.1561
JW5127	*ymbA*	OM-anchored periplasmic lipoprotein component of the putative pqiabc transporter, paraquat-inducible	0.3448	0.0000
JW5128	*yccS*	Putative transporter, FUSC superfamily inner membrane protein	-0.0013	0.9656
JW5129	*mgsA*	Methylglyoxal synthase	0.0177	0.7183
JW5130	*yccU*	Putative coa-binding protein	0.0580	0.1736
JW5131	*yccX*	Weak acylphosphatase	0.0487	0.3303
JW5132	*etp*	O-antigen capsule forming protein-tyrosine-phosphatase; Etk-P dephosphorylase	0.1088	0.0146
JW5133	*ymcD*	O-antigen capsule production threonine-rich inner membrane protein	0.0347	0.4480
JW5134	*cspH*	Cold shock-like protein csph	-0.0140	0.6556

JW5135	*torS*	Hybrid sensory histidine kinase in two-component regulatory system with torr	-0.0090	0.5815
JW5137	*ycdG*	Pyrimidine permease	-0.0115	0.7857
JW5138	*ycdH*	Flavin:NADH reductase	0.0876	0.0339
JW5139	*ycdL*	Ureidoacrylate amidohydrolase	0.0004	0.0010
JW5141	*ycdN*	CP4-44 prophage; putative disrupted hemin or colicin receptor	0.0028	0.9399
JW5142	*ycdR*	Poly-beta-1,6-N-acetyl-D-glucosamine (PGA) N-deacetylase outer membrane export lipoprotein	0.0691	0.0838
JW5143	*ycdT*	Diguanylate cyclase, membrane-anchored	0.0644	0.0356
JW5145	*ymdE*	CP4-44 prophage; putative disrupted hemin or colicin receptor	0.0253	0.5988
JW5146	*ycdW*	Glyoxylate/hydroxypyruvate reductase A	-0.1084	0.0588
JW5150	*ymdC*	Stationary phase cardiolipin synthase 3	0.0040	0.8850
JW5151	*yceK*	Outer membrane integrity lipoprotein	-0.0394	0.2707
JW5152	*yceP*	Biofilm regulator	-0.0036	0.9510
JW5153	*flgH*	Flagellar protein of basal-body outer-membrane L ring	-0.0501	0.2657
JW5155	*yceF*	M(7)GTP pyrophosphatase	-0.1333	0.0080
JW5156	*plsX*	Putative phosphate acyltransferase	-0.0951	0.1738
JW5157	*ycfM*	OM lipoprotein stimulator of mrcb transpeptidase	0.0468	0.0680
JW5158	*ycfP*	Putative UPF0227 family esterase	0.0803	0.0012
JW5159	*ycfQ*	Repressor for bhsa(ycfr)	0.0730	0.0249
JW5164	*ymfA*	DUF3592 family inner membrane protein	0.0007	0.1121
JW5165	*hflD*	Putative lysogenization regulator	-0.0566	0.1325
JW5166	*ymfE*	E14 prophage; putative inner membrane protein	-0.0593	0.4089
JW5168	*ymfI*	E14 prophage; uncharacterized protein	0.0411	0.3424
JW5169	*ymfT*	E14 prophage; putative DNA-binding transcriptional regulator	-0.1038	0.0032
JW5170	*ymfP*	Pseudogene, e14 prophage	-0.0385	0.2090
JW5172	*stfE*	Pseudogene, e14 prophage; side tail fiber protein fragment family	0.1415	0.0338
JW5173	*icdC*	CP4-44 prophage; putative disrupted hemin or colicin receptor	-0.1527	0.0000
JW5174	*ycgG*	Putative membrane-anchored cyclic-di-GMP phosphodiesterase	-0.0952	0.0085
JW5176	*ycgH*	CP4-44 prophage; putative disrupted hemin or colicin receptor	-0.0144	0.6743
JW5177	*ymgD*	Periplasmic protein, hdea structural homolog	0.0272	0.4402
JW5178	*ymgG*	UPF0757 protein ymgg	-0.0163	0.4779
JW5179	*ymgH*	CP4-44 prophage; putative disrupted hemin or colicin receptor	-0.1051	0.0300
JW5180	*ycgN*	UPF0153 family cysteine cluster protein	-0.1266	0.0846
JW5181	*hlyE*	Hemolysin E	-0.0251	0.5831
JW5184	*cvrA*	Putative cation/proton antiporter	-0.0809	0.0799
JW5185	*dhaH*	Uncharacterized protein	-0.0222	0.4570
JW5186	*dhaL*	Dihydroxyacetone kinase, C-terminal domain	0.0037	0.9216

JW5187	*dhaK*	Dihydroxyacetone kinase, PTS-dependent, dihydroxyacetone-binding subunit	-0.0293	0.4204
JW5188	*dhaR*	PTS-dependent dihydroxyacetone kinase operon regulatory protein	-0.1010	0.0441
JW5189	*ychM*	C4-dicarboxylic acid transporter	0.4440	0.0371
JW5195	*tonB*	Membrane spanning protein in tonb-exbb-exbd transport complex	0.3408	0.0061
JW5196	*yciO*	Putative RNA binding protein	-0.0475	0.1426
JW5197	*yciQ*	Enhancer of membrane protein expression; putative inner membrane protein	0.0864	0.0036
JW5198	*yciX*	CP4-44 prophage; putative disrupted hemin or colicin receptor	0.0149	0.7540
JW5199	*yciX*	CP4-44 prophage; putative disrupted hemin or colicin receptor	-0.0166	0.6771
JW5200	*yciW*	Putative oxidoreductase	0.1132	0.0063
JW5201	*puuA*	Glutamate--putrescine ligase	0.0522	0.0681
JW5202	*ycjR*	Putative TIM alpha/beta barrel enzyme	-0.1126	0.0855
JW5203	*ymjB*	CP4-44 prophage; putative disrupted hemin or colicin receptor	0.0154	0.5686
JW5205	*abgA*	P-aminobenzoyl-glutamate hydrolase subunit A	0.0473	0.1590
JW5206	*ydaM*	Diguanylate cyclase, csgd regulator	0.0430	0.6309
JW5207	*ydaQ*	Rac prophage; conserved protein	-0.0440	0.1522
JW5208	*lar*	Rac prophage; restriction alleviation protein	0.0457	0.1739
JW5209	*sieB*	Phage superinfection exclusion protein, Rac prophage	-0.0702	0.1919
JW5210	*ydaG*	Uncharacterized protein	0.0059	0.8135
JW5211	*ydaW*	Rac prophage; pseudogene, DNA-binding protein family	0.0240	0.5496
JW5212	*rzpR*	Pseudogene, Rac prophage; Bacteriophage Rz lysis protein family	-0.0702	0.2051
JW5213	*rzoR*	Rac prophage; putative lipoprotein	-0.0698	0.1612
JW5215	*ydbJ*	DUF333 family putative lipoprotein	0.0446	0.1815
JW5216	*ydbL*	DUF1318 family protein	0.0355	0.2816
JW5217	*paaD*	Ring 1,2-phenylacetyl-coa epoxidase subunit	0.0733	0.0538
JW5218	*paaK*	Phenylacetate-coenzyme A ligase	0.0007	0.0039
JW5221	*ydbD*	DUF2773 family methylglyoxal resistance protein	0.0628	0.0670
JW5224	*cybB*	Cytochrome b561	-0.0571	0.0798
JW5225	*hokB*	Toxic polypeptide, small	-0.0508	0.1963
JW5226	*ydcI*	Putative DNA-binding transcriptional regulator	0.0085	0.7597
JW5227	*yncK*	CP4-44 prophage; putative disrupted hemin or colicin receptor	-0.0481	0.0633
JW5228	*ydcM*	IS609 transposase B	-0.0778	0.0036
JW5229	*ydcO*	Bene family inner membrane putative transporter	0.0374	0.5638
JW5230	*yncN*	Mrna interferase toxin of the hicab toxin-antitoxin system	-0.0001	0.9977
JW5232	*ydcX*	DUF2566 family protein	0.0679	0.0117
JW5233	*yncA*	Methionine N-acyltransferase; L-amino acid N-acyltransferase	0.0119	0.7030

JW5234	*ansP*	L-asparagine transporter	-0.0795	0.0091
JW5235	*yncH*	IPR020099 family protein	0.0442	0.3481
JW5237	*yncM*	CP4-44 prophage; putative disrupted hemin or colicin receptor	-0.0544	0.1409
JW5238	*sfcA*	Malate dehydrogenase, decarboxylating, NAD-requiring; malic enzyme	-0.0328	0.2943
JW5239	*bdm*	Biofilm-dependent modulation protein	0.0023	0.9388
JW5240	*ddpA*	D,D-dipeptide ABC transporter periplasmic binding protein	-0.0860	0.0345
JW5241	*yddV*	Diguanylate cyclase, cold- and stationary phase-induced oxygen-dependent biofilm regulator	-0.0043	0.9140
JW5242	*yddA*	Putative multidrug ABC transporter permease/atpase	0.0051	0.8294
JW5243	*ydeN*	Putative Ser-type periplasmic non-aryl sulfatase	-0.0435	0.4243
JW5244	*yneL*	Pseudogene, arac family	-0.1236	0.0281
JW5245	*yneE*	Bestrophin family putative inner membrane protein	-0.0477	0.1328
JW5247	*yneI*	Succinate semialdehyde dehydrogenase, NAD(P)+-dependent	-0.0655	0.0296
JW5248	*marR*	Transcriptional repressor of multiple antibiotic resistance	0.0353	0.1588
JW5249	*marA*	Multiple antibiotic resistance transcriptional regulator	0.0603	0.0666
JW5250	*eamA*	Cysteine and O-acetyl-L-serine efflux system	-0.0036	0.9235
JW5251	*ynfO*	Uncharacterized protein, Qin prophage	-0.0578	0.2179
JW5252	*ydfO*	Qin prophage; DUF1398 family protein	-0.0211	0.6669
JW5253	*gnsB*	Qin prophage; multicopy suppressor of secg(Cs) and faba6(Ts)	0.0060	0.8907
JW5254	*ynfN*	Qin prophage; cold shock-induced protein	0.0229	0.2755
JW5255	*essQ*	Qin prophage; putative S lysis protein	0.0128	0.7281
JW5257	*ynfP*	CP4-44 prophage; putative disrupted hemin or colicin receptor	0.0192	0.5300
JW5258	*ynfC*	UPF0257 family lipoprotein	-0.0034	0.9224
JW5259	*ynfD*	DUF1161 family periplasmic protein	0.0029	0.9369
JW5260	*ynfF*	S- and N-oxide reductase, A subunit, periplasmic	-0.0321	0.3910
JW5261	*ynfH*	Oxidoreductase, membrane subunit	-0.1104	0.0053
JW5262	*dmsD*	Twin-argninine leader-binding protein for dmsa and tora	0.0800	0.1530
JW5263	*clcB*	H(+)/Cl(-) exchange transporter	-0.0874	0.0274
JW5264	*ynfK*	Putative dethiobiotin synthetase	-0.0215	0.2811
JW5265	*ydgJ*	Putative oxidoreductase	0.0224	0.4841
JW5267	*slyA*	Global transcriptional regulator	-0.0372	0.1038
JW5270	*ydhO*	Murein DD-endopeptidase, space-maker hydrolase	0.1036	0.0034
JW5271	*ydhX*	Putative 4Fe-4S ferridoxin-type protein; FNR, Nar, narp-regulated protein; putative subunit of ydhyvwxut oxidoreductase complex	0.0149	0.8180
JW5272	*ydhV*	Putative oxidoreductase subunit	0.0331	0.4364

JW5273	*sufB*	Component of sufbcd Fe-S cluster assembly scaffold	-0.0405	0.3297
JW5274	*ydiN*	Putative MFS transporter, membrane protein	-0.0075	0.8338
JW5275	*ydiO*	Putative acyl-coa dehydrogenase	0.0422	0.2909
JW5276	*ydiQ*	Putative electron transfer flavoprotein subunit	0.0022	0.9576
JW5278	*arpB*	CP4-44 prophage; putative disrupted hemin or colicin receptor	0.0130	0.7718
JW5280	*pfkB*	6-phosphofructokinase II	0.0419	0.2550
JW5281	*ydjM*	Inner membrane protein regulated by lexa	-0.0686	0.0652
JW5282	*astD*	Succinylglutamic semialdehyde dehydrogenase	-0.3010	0.0000
JW5283	*ydjY*	Putative ferrodoxin-like lipoprotein	0.0051	0.9027
JW5284	*ynjB*	Putative ABC transporter periplasmic binding protein	-0.1072	0.1064
JW5285	*ynjC*	Putative ABC transporter permease	0.0194	0.7025
JW5286	*ynjD*	Putative ABC transporter atpase	0.1425	0.0008
JW5287	*ynjE*	Molybdopterin synthase sulfurtransferase	0.0268	0.4603
JW5288	*ynjI*	Inner membrane protein	-0.0832	0.0280
JW5289	*ydjH*	Putative kinase	-0.0083	0.6745
JW5290	*ydjK*	Putative MFS sugar transporter, membrane protein	0.0147	0.7066
JW5291	*yeaJ*	Putative diguanylate cyclase	0.0171	0.5966
JW5292	*yeaP*	Diguanylate cyclase	0.0946	0.0608
JW5293	*yeaV*	Putative transporter	0.0430	0.1840
JW5294	*yeaW*	Putative yeawx dioxygenase alpha subunit; 2Fe-2S cluster	0.1092	0.0177
JW5295	*yoaB*	Putative reactive intermediate deaminase	0.0378	0.2420
JW5296	*yoaC*	DUF1889 family protein	0.0738	0.1112
JW5298	*yobH*	Uncharacterized protein	0.0043	0.8535
JW5299	*yebQ*	Putative transporter	-0.0222	0.5722
JW5300	*proQ*	RNA chaperone proq	-0.0330	0.3443
JW5301	*yebU*	16S rrna m(5)C1407 methyltransferase, SAM-dependent	0.0723	0.0970
JW5302	*yebV*	Uncharacterized protein	0.0507	0.1019
JW5303	*yebW*	Uncharacterized protein	-0.0628	0.1800
JW5304	*yebA*	Murein DD-endopeptidase, space-maker hydrolase, septation protein	0.1323	0.0197
JW5306	*yebB*	DUF830 family protein	0.0410	0.1388
JW5307	*yecD*	Isochorismatase family protein	0.0264	0.3532
JW5308	*yecN*	MAPEG family inner membrane protein	0.0928	0.1249
JW5309	*yecM*	Putative metal-binding enzyme	-0.1064	0.0112
JW5310	*yecT*	Uncharacterized protein	0.0272	0.3054
JW5312	*otsA*	Trehalose-6-phosphate synthase	-0.0172	0.2718
JW5313	*yedO*	D-cysteine desulfhydrase, PLP-dependent	0.0712	0.1400
JW5316	*fliO*	Flagellar biosynthesis protein	-0.0550	0.2617
JW5317	*yodD*	Uncharacterized protein	0.0085	0.8017

JW5319	*yedS*	CP4-44 prophage; putative disrupted hemin or colicin receptor	-0.0065	0.7986
JW5322	*yedW*	Response regulator family protein	-0.0663	0.0138
JW5323	*yodB*	Cytochrome b561 homolog	0.0532	0.0497
JW5325	*yeeL*	CP4-44 prophage; putative disrupted hemin or colicin receptor	0.0195	0.6448
JW5326	*yoeA*	CP4-44 prophage; putative disrupted hemin or colicin receptor	0.0566	0.2633
JW5327	*yeeP*	Pseudogene, CP4-44 prophage; 50S ribosome-binding gtpase family	0.1370	0.0011
JW5328	*yoeF*	Pseudogene, CP4-44 putative prophage remnant	-0.0437	0.3900
JW5329	*dacD*	D-alanyl-D-alanine carboxypeptidase; penicillin-binding protein 6b	-0.0002	0.0878
JW5330	*yeeF*	Putrescine importer, low affinity	-0.0269	0.5186
JW5331	*yoeB*	Toxin of the yoeb-yefm toxin-antitoxin system	-0.0014	0.9562
JW5335	*nudD*	GDP-mannose mannosyl hydrolase	0.0883	0.0037
JW5336	*yegH*	Inner membrane protein	-0.0337	0.2359
JW5338	*mdtA*	Multidrug efflux system, subunit A	0.0970	0.0094
JW5339	*yegP*	UPF0339 family protein	0.0661	0.0500
JW5340	*gatR*	CP4-44 prophage; putative disrupted hemin or colicin receptor	-0.1071	0.0000
JW5343	*gatY*	D-tagatose 1,6-bisphosphate aldolase 2, catalytic subunit	-0.0533	0.1967
JW5344	*fbaB*	Fructose-bisphosphate aldolase class I	-0.0435	0.1498
JW5345	*yegX*	Putative family 25 glycosyl hydrolase	0.0649	0.0256
JW5346	*yohN*	Periplasmic modulator of Ni and Co efflux	0.0211	0.3383
JW5349	*yehL*	Putative hexameric AAA+ moxr family atpase	-0.0143	0.6327
JW5350	*yehP*	VMA domain putative yehl atpase stimulator	0.1540	0.0055
JW5351	*yehR*	Lipoprotein, DUF1307 family	0.0158	0.5178
JW5352	*yehT*	Response regulator inducing btst; two-component system btssr	-0.0389	0.1293
JW5353	*yehU*	Sensory kinase regulating btst; two-component system btssr	0.0571	0.2162
JW5354	*yohO*	Putative membrane protein	0.1162	0.0557
JW5355	*pbpG*	D-alanyl-D-alanine endopeptidase	0.0558	0.1819
JW5356	*yohC*	Yip1 family inner membrane protein	-0.0431	0.4198
JW5358	*yohH*	CP4-44 prophage; putative disrupted hemin or colicin receptor	0.0023	0.9520
JW5359	*yeiS*	DUF2542 family protein	-0.0453	0.0815
JW5361	*yeiW*	UPF0153 cysteine cluster protein	-0.0265	0.5589
JW5362	*yeiP*	Elongation factor P-like protein	0.0337	0.4442
JW5363	*bcr*	Bicyclomycin/cysteine/sulfonamide efflux transporter	-0.0421	0.1776
JW5366	*ccmA*	Heme export ABC transporter atpase	0.0062	0.7883
JW5367	*napB*	Nitrate reductase, small, cytochrome C550 subunit, periplasmic	-0.0662	0.0726
JW5368	*yojL*	Putative thiamine-synthetic flavin transferase lipoprotein	0.1503	0.0001

JW5371	*yfaZ*	Outer membrane protein, putative porin	0.0271	0.1461
JW5372	*yfbE*	Uridine 5'-(beta-1-threo-pentapyranosyl-4-ulose diphosphate) aminotransferase, PLP-dependent	-0.0982	0.0033
JW5373	*yfbJ*	Undecaprenyl phosphate-alpha-L-ara4n exporter; flippase arnef subunit	-0.0425	0.1923
JW5374	*menD*	2-succinyl-5-enolpyruvyl-6-hydroxy-3-cyclohexene-1-carboxylate synthase; SEPHCHC synthase	0.0015	0.9535
JW5375	*nuoC*	NADH:ubiquinone oxidoreductase, fused CD subunit	0.1487	0.0220
JW5376	*yfbT*	Hexitol phosphatase A	-0.0598	0.2595
JW5377	*yfcE*	Phosphodiesterase activity on bis-pnpp	0.0316	0.3230
JW5378	*dedD*	Membrane-anchored periplasmic protein involved in septation	-0.0353	0.5595
JW5380	*trmC*	Fused 5-methylaminomethyl-2-thiouridine-forming enzyme methyltransferase and FAD-dependent demodification enzyme	-0.0331	0.3922
JW5381	*yfcM*	Elongation Factor P Lys34 hydroxylase	0.0001	0.9963
JW5382	*yfdI*	Serotype-specific glucosyl transferase, CPS-53 (kple1) prophage	0.0424	0.1238
JW5384	*yfdL*	Pseudogene, CPS-53 (kple1) prophage	0.0142	0.6600
JW5387	*torI*	Response regulator inhibitor for tor operon	-0.0744	0.1410
JW5388	*ypdA*	Sensor kinase regulating yhjx; pyruvate-responsive ypdab two-component system	-0.0256	0.5285
JW5389	*ypdH*	Putative enzyme IIB component of PTS	-0.0049	0.9052
JW5391	*yfeA*	Putative diguanylate cyclase	0.0112	0.7771
JW5394	*ucpA*	Furfural resistance protein, putative short-chain oxidoreductase	-0.0116	0.6141
JW5395	*yfeW*	Penicillin binding protein PBP4B; weak DD-carboxypeptidase activity	0.0464	0.1470
JW5396	*ypfH*	Palmitoyl-coa esterase activity, uncertain physiological substrate	-0.0465	0.3737
JW5397	*hda*	Atpase regulatory factor involved in dnaa inactivation	-0.0450	0.0618
JW5399	*yfgG*	Uncharacterized protein	-0.0078	0.8281
JW5400	*yfgH*	Outer membrane integrity lipoprotein	-0.0120	0.8031
JW5401	*guaB*	IMP dehydrogenase	-0.0276	0.2688
JW5402	*yfgJ*	DUF1407 family protein	-0.0205	0.2841
JW5404	*sseB*	Rhodanase-like enzyme, sulfur transfer from thiosulfate	0.0278	0.5021
JW5405	*yphG*	DUF4380 domain-containing TPR repeat protein	-0.0090	0.6886
JW5406	*yphH*	Putative DNA-binding transcriptional regulator	0.0372	0.3418
JW5407	*yfhK*	Sensor protein kinase regulating glmy srna in two-component system with response regulator glrr	0.0343	0.3679
JW5408	*yfhB*	Phosphatidylglycerophosphatase C, membrane bound	-0.1049	0.0046
JW5409	*yfiP*	DTW domain protein	-0.0579	0.1382
JW5412	*yfiL*	Lipoprotein	-0.0409	0.1756

JW5413	*rimM*	Ribosome maturation factor	-0.2984	0.0006
JW5415	*yfjD*	UPF0053 family inner membrane protein	-0.0209	0.5283
JW5416	*recN*	Recombination and repair protein	-0.0086	0.8236
JW5418	*yfjO*	CP4-57 prophage; uncharacterized protein	0.0343	0.4329
JW5419	*yfjP*	CP4-57 prophage; 50S ribosome-binding gtpase family protein	0.1371	0.0213
JW5420	*ypjM*	CP4-44 prophage; putative disrupted hemin or colicin receptor	0.0163	0.7958
JW5421	*ypjJ*	Uncharacterized protein	-0.0174	0.3634
JW5422	*ypjA*	Adhesin-like autotransporter	-0.1070	0.0008
JW5424	*ypjC*	Uncharacterized protein	0.0500	0.2442
JW5425	*ygaQ*	CP4-44 prophage; putative disrupted hemin or colicin receptor	0.0322	0.2784
JW5426	*yqaC*	CP4-44 prophage; putative disrupted hemin or colicin receptor	0.0217	0.6945
JW5427	*ygaT*	Carbon starvation protein	-0.0464	0.1397
JW5428	*ygaY*	CP4-44 prophage; putative disrupted hemin or colicin receptor	-0.0051	0.8837
JW5429	*srlA*	Glucitol/sorbitol-specific enzyme IIC component of PTS	-0.0282	0.4723
JW5430	*srlE*	Glucitol/sorbitol-specific enzyme IIB component of PTS	-0.0868	0.0097
JW5431	*gutQ*	D-arabinose 5-phosphate isomerase	0.0497	0.1470
JW5433	*hypF*	Carbamoyl phosphate phosphatase and [nife] hydrogenase maturation protein	0.1155	0.0200
JW5434	*ascG*	Asc operon transcriptional repressor; prpbc operon repressor	-0.0225	0.2512
JW5435	*ascF*	Cellobiose/arbutin/salicin-specific PTS enzymes, IIB and IC components	0.0358	0.2781
JW5437	*rpoS*	RNA polymerase, sigma S (sigma 38) factor	0.0351	0.4698
JW5438	*ygbF*	CRISPR adaptation ssrna endonuclease	0.1775	0.0009
JW5440	*ygcQ*	Putative flavoprotein	0.0349	0.2701
JW5441	*ygcR*	Putative flavoprotein	-0.0778	0.0725
JW5442	*ygcU*	Putative FAD-linked oxidoreductase	-0.0143	0.6573
JW5443	*ygcW*	Putative SDR family oxidoreductase	-0.0489	0.2900
JW5444	*ygcE*	Putative kinase	0.0029	0.9411
JW5445	*ygcG*	TPM domain protein, putative phosphatase	-0.0230	0.5546
JW5446	*exo*	Ssb-binding protein, misidentified as exoix	0.0385	0.3067
JW5448	*ygdI*	DUF903 family verified lipoprotein	0.0030	0.9351
JW5449	*amiC*	N-acetylmuramoyl-L-alanine amidase	-0.0019	0.9256
JW5450	*ygdB*	DUF2509 family protein	-0.0220	0.4573
JW5451	*ppdB*	Putative prepilin peptidase-dependent protein	0.0411	0.3561
JW5453	*yqeF*	Short chain acyltransferase	-0.0624	0.0436
JW5454	*yqeH*	Putative luxr family transcriptional regulator	0.1268	0.0105
JW5455	*yqeJ*	Uncharacterized protein	-0.0383	0.4357
JW5456	*ygeI*	Uncharacterized protein	-0.0006	0.9917

JW5457	*pbl*	CP4-44 prophage; putative disrupted hemin or colicin receptor	-0.0308	0.3569
JW5458	*ygeK*	CP4-44 prophage; putative disrupted hemin or colicin receptor	0.0319	0.3697
JW5459	*ygeM*	CP4-44 prophage; putative disrupted hemin or colicin receptor	-0.0188	0.4184
JW5460	*ygeN*	CP4-44 prophage; putative disrupted hemin or colicin receptor	0.0785	0.0030
JW5461	*ygeQ*	Uncharacterized protein	0.0433	0.2470
JW5462	*xdhA*	Xanthine dehydrogenase, molybdenum binding subunit	-0.0027	0.9133
JW5463	*ygeW*	Putative carbamoyltransferase	0.0448	0.1366
JW5464	*yqeC*	Putative selenium-dependent hydroxylase accessory protein	0.0048	0.8061
JW5466	*guaD*	Guanine deaminase	-0.0481	0.2055
JW5467	*ygfQ*	Guanine/hypoxanthine permease, high affinity; guanine/hypoxanthine:H+ symporter	-0.0078	0.8028
JW5468	*ygfS*	Putative 4Fe-4S ferredoxin-type oxidoreductase subunit	0.0288	0.3637
JW5469	*ygfT*	Putative oxidoreductase, Fe-S subunit/nucleotide-binding subunit	-0.0186	0.5712
JW5470	*ygfU*	Uric acid permease	-0.0551	0.3218
JW5473	*ygfB*	UPF0149 family protein	0.0274	0.3221
JW5475	*rpiA*	Ribose 5-phosphate isomerase, constitutive	-0.0064	0.8921
JW5476	*ygfI*	Putative DNA-binding transcriptional regulator	-0.0339	0.1466
JW5477	*yggP*	Putative Zn-binding dehydrogenase	-0.0139	0.6421
JW5478	*tktA*	Transketolase 1, thiamine triphosphate-binding	-0.0578	0.2939
JW5479	*yggU*	UPF0235 family protein	-0.0240	0.3497
JW5481	*mltC*	Membrane-bound lytic murein transglycosylase C	0.0186	0.7146
JW5482	*speC*	Ornithine decarboxylase, constitutive	-0.0049	0.8282
JW5484	*yghF*	Pseudogene, secretion pathway protein, C-type protein homology	0.0209	0.6242
JW5486	*glcF*	Glycolate oxidase 4Fe-4S iron-sulfur cluster subunit	0.0800	0.1205
JW5487	*glcE*	Glycolate oxidase FAD binding subunit	0.0017	0.9519
JW5490	*yghQ*	Putative inner membrane polysaccharide flippase	0.0697	0.0000
JW5491	*yghS*	Putative ATP-binding protein	0.0294	0.1229
JW5492	*yghU*	Putative S-transferase	-0.0218	0.3089
JW5493	*hybF*	Protein involved with the maturation of hydrogenases 1 and 2	-0.0206	0.3843
JW5494	*hybB*	Putative hydrogenase 2 cytochrome b type component	-0.0047	0.8717
JW5496	*yghY*	CP4-44 prophage; putative disrupted hemin or colicin receptor	-0.0542	0.1257
JW5499	*dkgA*	2,5-diketo-D-gluconate reductase A	0.0255	0.1095
JW5500	*yqhG*	DUF3828 family putative periplasmic protein	-0.0151	0.5716
JW5501	*ygiQ*	Radical SAM superfamily protein	0.0147	0.5822
JW5502	*ygiV*	Probable transcriptional regulator ygiv	0.0444	0.2187

JW5503	*tolC*	Transport channel	-0.5387	0.0000
JW5507	*yqiG*	Pseudogene; fimbrial export usher family	0.0487	0.1569
JW5508	*yqiH*	Putative periplasmic pilin chaperone	-0.0082	0.8178
JW5509	*yqiI*	Fimbrial protein	-0.0714	0.0757
JW5510	*ygjG*	Putrescine:2-oxoglutaric acid aminotransferase, PLP-dependent	-0.0545	0.1062
JW5511	*ebgA*	Evolved beta-D-galactosidase, alpha subunit	-0.2174	0.0003
JW5512	*ygjI*	Putative transporter	-0.0027	0.8591
JW5513	*ygjO*	23S rrna m(2)G1835 methyltransferase, SAM-dependent	-0.0623	0.1674
JW5514	*ygjP*	UTP pyrophosphatase	0.0457	0.3199
JW5515	*alx*	Putative membrane-bound redox modulator	0.0512	0.1793
JW5516	*yqjC*	DUF1090 family putative periplasmic protein	-0.0087	0.7751
JW5517	*yhaL*	Uncharacterized protein	0.0377	0.2852
JW5518	*yhaM*	Putative L-serine dehydratase alpha chain	0.0834	0.0253
JW5519	*yhaO*	Putative transporter	0.0214	0.5777
JW5520	*tdcG*	L-serine dehydratase 3, anaerobic	0.0515	0.1655
JW5522	*tdcE*	Pyruvate formate-lyase 4/2-ketobutyrate formate-lyase	-0.0103	0.6843
JW5525	*tdcR*	L-threonine dehydratase operon activator protein	-0.0511	0.4366
JW5526	*garR*	Tartronate semialdehyde reductase	0.0455	0.0446
JW5527	*agaA*	Pseudogene, N-acetylgalactosamine-6-phosphate deacetylase fragment	0.0084	0.6851
JW5528	*yraR*	Putative nucleoside-diphosphate-sugar epimerase	-0.0113	0.7636
JW5529	*yhbO*	Stress-resistance protein	0.0712	0.1125
JW5531	*deaD*	ATP-dependent RNA helicase	-0.0061	0.8926
JW5533	*yhbC*	Ribosome maturation factor for 30S subunits	0.0210	0.6925
JW5534	*yhbX*	Putative eptab family phosphoethanolamine transferase, inner membrane protein	0.0677	0.1186
JW5536	*arcB*	Aerobic respiration control sensor histidine protein kinase, cognate to two-component response regulators arca and rssb	-0.0261	0.6282
JW5538	*nanK*	N-acetylmannosamine kinase	0.0036	0.9170
JW5539	*yhcB*	DUF1043 family inner membrane-anchored protein	-0.0234	0.4229
JW5540	*yhcN*	Cadmium and peroxide resistance protein, stress-induced	0.0058	0.8228
JW5542	*yhdP*	DUF3971-asma2 domains protein	-0.0020	0.9361
JW5543	*yhdJ*	DNA adenine methyltransferase, SAM-dependent	-0.0034	0.8987
JW5544	*yhdX*	Putative amino acid ABC transporter permease	0.0334	0.3161
JW5545	*yhdY*	Putative amino acid ABC transporter permease	-0.0735	0.0891
JW5546	*zraP*	Zn-dependent periplasmic chaperone	0.0132	0.7212
JW5547	*nfi*	Endonuclease V; deoxyinosine 3' endonuclease	0.0437	0.1863
JW5548	*nudC*	NADH pyrophosphatase	-0.0568	0.3859
JW5549	*thiG*	Thiamine biosynthesis thigh complex subunit	0.0048	0.8937
JW5551	*sthA*	Pyridine nucleotide transhydrogenase, soluble	-0.0556	0.1372

JW5553	*argB*	Acetylglutamate kinase	-0.0288	0.4398
JW5555	*ptsA*	Putative PTS enzyme: Hpr, enzyme I and II components	0.0433	0.1458
JW5556	*gldA*	Glycerol dehydrogenase, NAD+ dependent; 1,2-propanediol:NAD+ oxidoreductase	-0.1060	0.0076
JW5557	*yijE*	Eama-like transporter family protein	0.0202	0.5577
JW5558	*cpxP*	Inhibitor of the cpx response; periplasmic adaptor protein	-0.0048	0.8473
JW5559	*yiiM*	6-N-hydroxylaminopurine resistance protein	-0.0770	0.0793
JW5560	*kdgT*	2-keto-3-deoxy-D-gluconate transporter	-0.0129	0.7321
JW5562	*frvB*	Putative PTS enzyme, IIB component/IIC component	0.0380	0.3265
JW5563	*yiiF*	Putative thymol sensitivity protein, copg family putative transcriptional regulator	0.0024	0.9385
JW5566	*yihX*	Alpha-D-glucose-1-phosphate phosphatase, anomer-specific	-0.0367	0.4617
JW5567	*yihW*	Putative transcriptional regulator for sulphoquinovose utilization	-0.0291	0.4231
JW5568	*yihV*	6-deoxy-6-sulphofructose kinase	0.0991	0.0897
JW5569	*yihS*	Sulphoquinovose isomerase	0.0072	0.7730
JW5571	*bipA*	GTP-binding protein	-0.2043	0.0002
JW5574	*yihF*	DUF945 family protein	-0.0377	0.1865
JW5575	*mobB*	Molybdopterin-guanine dinucleotide biosynthesis protein B	0.0332	0.0522
JW5576	*trkH*	Potassium transporter	0.0833	0.0369
JW5577	*yigZ*	UPF0029 family protein	-0.0874	0.0867
JW5578	*fadA*	3-ketoacyl-coa thiolase (thiolase I)	0.0336	0.3791
JW5580	*tatB*	Tatabce protein translocation system subunit	0.0377	0.2454
JW5581	*ubiE*	Bifunctional 2-octaprenyl-6-methoxy-1,4-benzoquinone methylase/ S-adenosylmethionine:2-DMK methyltransferase	-0.1514	0.0018
JW5584	*pldB*	Lysophospholipase L2	-0.0515	0.1768
JW5585	*rhtB*	Homoserine, homoserine lactone and S-methyl-methionine efflux pump	-0.0225	0.4473
JW5586	*rhtC*	Threonine efflux pump	0.0323	0.4360
JW5588	*yigI*	4HBT thioesterase family protein	-0.0538	0.0584
JW5589	*rarD*	Putative chloramphenical resistance permease	0.0109	0.8154
JW5590	*yigG*	PRK11371 family inner membrane protein	0.0714	0.0197
JW5591	*yigE*	DUF2233 family protein	-0.0493	0.2884
JW5592	*dapF*	Diaminopimelate epimerase	0.1032	0.0636
JW5594	*aslB*	Putative asla-specific sulfatase-maturating enzyme	-0.0027	0.8336
JW5595	*yifK*	Putative APC family amino acid transporter	0.1803	0.0031
JW5596	*rffT*	TDP-Fuc4NAc:lipidiifuc4nac transferase	-0.0543	0.0188
JW5597	*rffC*	TDP-fucosamine acetyltransferase	-0.0193	0.4722
JW5599	*rffD*	UDP-N-acetyl-D-mannosaminuronic acid dehydrogenase	0.0779	0.0179

JW5600	*rffE*	UDP-N-acetyl glucosamine-2-epimerase	0.0531	0.2754
JW5601	*wzzE*	Entobacterial Common Antigen (ECA) polysaccharide chain length modulation protein	-0.0297	0.3669
JW5603	*gpp*	Guanosine pentaphosphatase/exopolyphosphatase	0.0329	0.3993
JW5604	*rep*	DNA helicase and single-stranded DNA-dependent atpase	-0.0075	0.6027
JW5605	*ilvD*	Dihydroxyacid dehydratase	-0.0101	0.7526
JW5606	*ilvE*	Branched-chain amino acid aminotransferase	0.0385	0.4716
JW5607	*hdfR*	Flhdc operon transcriptional repressor	0.0172	0.5031
JW5608	*yieP*	Putative transcriptional regulator	-0.0526	0.4039
JW5609	*trkD*	Potassium transporter	0.0318	0.0718
JW5610	*yieM*	Stimulator of rava atpase activity; von Willebrand factor domain protein	0.0072	0.7084
JW5611	*atpI*	ATP synthase, membrane-bound accessory factor	-0.0137	0.6386
JW5612	*yieL*	Putative xylanase	0.0702	0.2540
JW5613	*yieK*	Putative 6-phosphogluconolactonase	0.1396	0.0222
JW5619	*tnaB*	CP4-44 prophage; putative disrupted hemin or colicin receptor	0.0662	0.0725
JW5627	*dgoR*	D-galactonate catabolism operon transcriptional repressor	-0.0129	0.5108
JW5628	*dgoA*	2-oxo-3-deoxygalactonate 6-phosphate aldolase	-0.0039	0.9317
JW5629	*dgoD*	D-galactonate dehydratase	0.0187	0.6576
JW5631	*cbrA*	Colicin M resistance protein; FAD-binding protein, putative oxidoreductase	0.0848	0.0294
JW5633	*yidQ*	DUF1375 family outer membrane protein	-0.0241	0.3885
JW5634	*emrD*	Multidrug efflux system protein	-0.0222	0.4377
JW5636	*yicO*	Adenine permease, high affinity; adenine:H+ symporter	-0.0207	0.5749
JW5637	*yicN*	DUF1198 family protein	0.0028	0.9568
JW5641	*ttk*	Nucleoid occlusion factor, anti-ftsz division inhibitor	0.0017	0.9639
JW5643	*yicR*	UPF0758 family protein	-0.0434	0.0334
JW5644	*htrL*	Yibb family protein, function unknown	0.0591	0.0945
JW5645	*yibQ*	Putative polysaccharide deacetylase	-0.0357	0.3502
JW5646	*envC*	Activator of amib,C murein hydrolases, septal ring factor	0.2035	0.0012
JW5648	*yiaY*	L-threonine dehydrogenase	-0.0404	0.4030
JW5650	*sgbU*	Putative L-xylulose 5-phosphate 3-epimerase	-0.0875	0.1562
JW5651	*yiaN*	2,3-diketo-L-gulonate TRAP transporter large permease protein	-0.0199	0.7674
JW5652	*avtA*	Valine-pyruvate aminotransferase; transaminase C; alanine-valine transaminase	0.3840	0.0008
JW5653	*bax*	Putative glucosaminidase	-0.1328	0.1362
JW5655	*yiaF*	Barrier effect co-colonization resistance factor; DUF3053 family lipoprotein	-0.0842	0.0292
JW5656	*tiaE*	Glyoxylate/hydroxypyruvate reductase B	0.0177	0.3164
JW5659	*yhjY*	Autotransporter beta-domain protein	-0.0678	0.0778

JW5660	*eptB*	KDO phosphoethanolamine transferase, Ca(2+)-inducible	0.0343	0.4548
JW5663	*bcsF*	DUF2636 family cellulose production small membrane protein	0.0552	0.2277
JW5665	*bcsA*	Cellulose synthase, catalytic subunit	-0.0300	0.2441
JW5668	*kdgK*	2-dehydro-3-deoxygluconokinase	-0.0217	0.6316
JW5669	*hdeB*	Acid-resistance protein	0.0189	0.7178
JW5670	*yhiD*	Putative Mg(2+) transport atpase, inner membrane protein	-0.0265	0.5640
JW5672	*yhiQ*	16S rrna m(2)G1516 methyltransferase, SAM-dependent	0.0311	0.1823
JW5674	*yhiK*	CP4-44 prophage; putative disrupted hemin or colicin receptor	0.0901	0.0058
JW5676	*rbbA*	Ribosome-associated atpase: ATP-binding protein/ATP-binding membrane protein	0.0300	0.3099
JW5677	*yhhJ*	Putative ABC transporter permease	-0.0026	0.9382
JW5678	*yrhC*	CP4-44 prophage; putative disrupted hemin or colicin receptor	-0.0147	0.8058
JW5679	*rhsB*	Rhs protein with DUF4329 family putative toxin domain; putative neighboring cell growth inhibitor	-0.0725	0.0981
JW5680	*yhhT*	UPF0118 family putative transporter	0.0015	0.0390
JW5682	*dcrB*	Putative lipoprotein	0.0274	0.4426
JW5683	*yhhL*	DUF1145 family protein	0.0381	0.3048
JW5686	*gntU*	Gluconate transporter, low affinity GNT 1 system	-0.0614	0.0838
JW5687	*glpG*	Rhomboid intramembrane serine protease	0.0221	0.5222
JW5688	*rtcA*	RNA 3'-terminal phosphate cyclase	0.0496	0.1244
JW5689	*malP*	Maltodextrin phosphorylase	0.0093	0.7025
JW5690	*gntT*	Gluconate transporter, high-affinity GNT I system	0.0564	0.1765
JW5691	*gntX*	DNA catabolic protein	-0.0174	0.7455
JW5692	*hslO*	Heat shock protein Hsp33	0.0219	0.7581
JW5693	*yrfD*	DNA catabolic putative pilus assembly protein	0.6202	0.0000
JW5694	*yrfA*	DNA catabolic protein	0.0014	0.9739
JW5696	*yhfY*	PRD domain protein	-0.0395	0.4800
JW5697	*yhfU*	DUF2620 family protein	0.0170	0.5894
JW5698	*frlR*	Putative DNA-binding transcriptional regulator	-0.0305	0.3029
JW5699	*frlC*	Fructoselysine 3-epimerase	0.0201	0.5825
JW5700	*frlB*	Fructoselysine-6-P-deglycase	-0.0361	0.3464
JW5701	*yhfK*	Putative transporter, FUSC superfamily inner membrane protein	0.1075	0.0003
JW5702	*crp*	Camp-activated global transcription factor, mediator of catabolite repression	0.0094	0.8515
JW5703	*yheO*	Putative PAS domain-containing DNA-binding transcriptional regulator	-0.0779	0.0990
JW5704	*gspM*	General secretory pathway component, cryptic	0.0254	0.3516
JW5705	*gspL*	General secretory pathway component, cryptic	0.1327	0.0037
JW5706	*gspI*	General secretory pathway component, cryptic	0.0116	0.6431

JW5707	*gspD*	General secretory pathway component, cryptic	-0.0002	0.9944
JW5708	*smf*	DNA recombination-mediator A family protein	0.0321	0.4463
JW5710	*yrdA*	Bacterial transferase hexapeptide domain protein	-0.0645	0.3149
JW5711	*yjbF*	Extracellular polysaccharide production lipoprotein	0.0236	0.7148
JW5713	*ubiC*	Chorismate--pyruvate lyase	-0.0088	0.8275
JW5714	*zur*	Transcriptional repressor, Zn(II)-binding	0.0335	0.5561
JW5716	*yjbO*	Phage shock protein G	0.0064	0.9161
JW5718	*yjcB*	Putative inner membrane protein	-0.0298	0.2867
JW5721	*yjcS*	Putative alkylsulfatase; SDS catabolic enzyme	0.0030	0.8303
JW5727	*phnK*	Carbon-phosphorus lyase complex subunit, putative ATP transporter ATP-binding protein	-0.0431	0.1303
JW5729	*yjcZ*	Yjcz family protein; yhjh motility defect suppressor	0.0059	0.8561
JW5730	*eptA*	Phosphoethanolamine transferase epta	-0.0545	0.2094
JW5731	*adiA*	Arginine decarboxylase	-0.0521	0.2973
JW5732	*yjdO*	Toxin ghot	0.0007	0.9812
JW5733	*yjdC*	Putative transcriptional regulator	0.0153	0.5941
JW5734	*dipZ*	Thiol:disulfide interchange protein and activator of dsbc	-0.0305	0.4895
JW5735	*dcuA*	C4-dicarboxylate antiporter	-0.0448	0.1303
JW5736	*yjeI*	DUF4156 family lipoprotein	0.0093	0.7551
JW5737	*ecnA*	Entericidin A membrane lipoprotein, antidote entericidin B	-0.0520	0.4278
JW5738	*sugE*	Multidrug efflux system protein	-0.0109	0.8413
JW5739	*yjeM*	Putative transporter	0.0768	0.0012
JW5741	*rnr*	Exoribonuclease R, rnase R	-0.0354	0.3417
JW5742	*yjfN*	DUF1471 family periplasmic protein	0.0523	0.0547
JW5743	*yjfO*	Bioflm peroxide resistance protein	0.0141	0.6006
JW5744	*ulaA*	L-ascorbate-specific enzyme IIC permease component of PTS	0.0218	0.5155
JW5745	*ytfB*	Oapa family protein	0.0230	0.5032
JW5746	*fklB*	FKBP-type peptidyl-prolyl cis-trans isomerase (rotamase)	-0.0604	0.1461
JW5747	*ytfH*	DUF24 family hxlr-type putative transcriptional regulator	0.0119	0.6692
JW5748	*ytfI*	Uncharacterized protein	0.0349	0.2617
JW5749	*ytfK*	DUF1107 family protein	-0.0378	0.4577
JW5752	*ytfR*	Putative sugar ABC transporter atpase	0.0145	0.6574
JW5753	*ytfT*	Putative sugar ABC transporter permease	-0.0310	0.2016
JW5754	*yjfF*	Putative sugar ABC transporter permease	0.0835	0.0667
JW5755	*yjgF*	Enamine/imine deaminase, reaction intermediate detoxification	0.0852	0.1585
JW5756	*yjgK*	Biofilm modulator regulated by toxins; DUF386 family protein, cupin superfamily protein	0.0952	0.0057
JW5757	*yjgL*	Sopa-central-domain-like hexapeptide repeat protein	-0.0134	0.7256

JW5758	*yjgM*	GNAT family putative N-acetyltransferase	-0.0138	0.5957
JW5759	*yjgN*	DUF898 family inner membrane protein	0.0247	0.5559
JW5761	*yjgB*	Broad specificity NADPH-dependent aldehyde reductase, Zn-containing	0.0006	0.9864
JW5763	*yjgX*	CP4-44 prophage; putative disrupted hemin or colicin receptor	-0.0240	0.4913
JW5764	*yjgX*	CP4-44 prophage; putative disrupted hemin or colicin receptor	-0.0193	0.4979
JW5768	*yjhB*	Putative MFS transporter, membrane protein	0.0211	0.4408
JW5769	*yjhC*	GFO/IDH/MOCA family putative oxidoreductase. NAD(P)-dependent	-0.0023	0.9588
JW5770	*yjhD*	CP4-44 prophage; putative disrupted hemin or colicin receptor	0.0815	0.0252
JW5775	*yjhH*	Putative lyase/synthase	0.0194	0.5301
JW5776	*sgcX*	Putative endoglucanase with Zn-dependent exopeptidase domain	-0.0083	0.8183
JW5777	*yjhT*	N-acetylneuraminate epimerase	-0.0319	0.3722
JW5778	*yjhA*	N-acetylnuraminic acid outer membrane channel protein	0.0671	0.0185
JW5780	*fimD*	Fimbrial usher outer membrane porin protein; fimcd chaperone-usher	0.0213	0.7061
JW5782	*yjiD*	Rpos stabilzer after DNA damage, anti-rssb factor	-0.0487	0.1656
JW5783	*yjiH*	Nucleoside recognition pore and gate family putative inner membrane transporter	0.0749	0.0381
JW5784	*kptA*	RNA 2'-phosphotransferase	-0.0194	0.5097
JW5785	*yjiL*	Putative atpase, activator of (R)-hydroxyglutaryl-coa dehydratase	0.0003	0.9908
JW5786	*yjiM*	Putative 2-hydroxyglutaryl-coa dehydratase	-0.0839	0.0348
JW5787	*yjiT*	Pseudogene	-0.0120	0.8055
JW5789	*mcrC*	5-methylcytosine-specific restriction enzyme mcrbc, subunit mcrc	-0.0268	0.6326
JW5790	*yjiA*	Metal-binding gtpase	-0.0157	0.8083
JW5791	*yjiY*	Pyruvate/H+ symporter	-0.0303	0.4414
JW5792	*yjjM*	Putative transcriptional activator for L-galactonate catabolism	-0.4007	0.0004
JW5793	*yjjN*	L-galactonate oxidoreductase; L-gulonate oxidoreductase	0.0580	0.0439
JW5794	*mdoB*	OPG periplasmic biosynthetic phosphoglycerol transferases I (membrane-bound) and II (soluble);	0.0383	0.1441
JW5795	*yjjA*	Putative DUF2501 family periplasmic protein	-0.1178	0.0035
JW5796	*yjjP*	DUF1212 family inner membrane protein	0.0386	0.4007
JW5797	*yjjZ*	Uncharacterized protein	0.0455	0.0400
JW5800	*nadR*	Trifunctional protein: nicotinamide mononucleotide adenylyltransferase, ribosylnicotinamide kinase, transcriptional repressor	0.0493	0.3087
JW5801	*yjjX*	Non-canonical purine NTP phosphatase, itpase/xtpase	-0.0643	0.0656
JW5802	*ydbA*	CP4-44 prophage; putative disrupted hemin or colicin receptor	-0.0494	0.2204

JW5803	*ybhR*	Putative ABC transporter permease	-0.0247	0.3646
JW5804	*ycjY*	S9 homolog non-peptidase family protein	-0.0486	0.0354
JW5805	*hyfI*	Hydrogenase 4, Fe-S subunit	-0.0864	0.0128
JW5806	*tdcD*	Propionate kinase/acetate kinase C, anaerobic	0.0371	0.3158
JW5808	*pcnB*	Poly(A) polymerase	-0.0540	0.1110
JW5811	*fhiA*	Pseudogene, flagellar system protein, promoterless fragment	-0.1108	0.1283
JW5812	*mbhA*	Pseudogene, lateral flagellar motor protein fragment	0.0034	0.9331
JW5813	*ykfC*	CP4-6 prophage; conserved protein	0.0340	0.3222
JW5814	*yaiF*	CP4-44 prophage; putative disrupted hemin or colicin receptor	0.0400	0.0664
JW5815	*tfaD*	CP4-44 prophage; putative disrupted hemin or colicin receptor	0.0435	0.4100
JW5816	*ybfE*	Lexa-regulated protein, copb family	-0.0009	0.9724
JW5818	*potG*	Putrescine ABC transporter atpase	0.0954	0.0428
JW5819	*ybjS*	Putative NAD(P)H-dependent oxidoreductase	-0.0585	0.2233
JW5820	*ycfS*	L,D-transpeptidase linking Lpp to murein	0.0092	0.8862
JW5821	*emtA*	Lytic murein endotransglycosylase E	0.0633	0.0028
JW5822	*abgT*	P-aminobenzoyl-glutamate transporter; membrane protein	-0.1196	0.0252
JW5823	*ydcH*	DUF465 family protein	-0.0550	0.2892
JW5825	*yneF*	Putative membrane-bound diguanylate cyclase	0.1571	0.1001
JW5826	*asr*	Acid shock-inducible periplasmic protein	0.2467	0.0037
JW5827	*ydhL*	DUF1289 family protein	-0.0529	0.3469
JW5830	*yebN*	Putative Mn(2+) efflux pump, mntr-regulated	0.0000	0.7018
JW5831	*znuA*	Zinc ABC transporter periplasmic binding protein	0.0448	0.1602
JW5832	*yedQ*	Putative membrane-anchored diguanylate cyclase	0.0080	0.8024
JW5834	*yeeY*	Lysr family putative transcriptional regulator	-0.0302	0.5455
JW5836	*cld*	Regulator of length of O-antigen component of lipopolysaccharide chains	0.0570	0.1731
JW5837	*yegR*	Uncharacterized protein	0.0004	0.0092
JW5838	*yohG*	CP4-44 prophage; putative disrupted hemin or colicin receptor	0.0219	0.6944
JW5839	*yejO*	Pseudogene, autotransporter outer membrane homology	0.0125	0.6316
JW5840	*elaD*	Protease, capable of cleaving an AMC-ubiquitin model substrate	-0.1019	0.0582
JW5841	*prmB*	N5-glutamine methyltransferase	0.0368	0.5464
JW5842	*yphC*	Putative Zn-dependent NAD(P)-binding oxidoreductase	-0.1124	0.0006
JW5843	*norR*	Anaerobic nitric oxide reductase DNA-binding transcriptional activator	-0.0219	0.5326
JW5844	*ygcI*	CRISP RNA (crrna) containing Cascade antiviral complex protein	-0.1490	0.0037
JW5845	*ygcS*	Putative MFS sugar transporter; membrane protein	-0.1217	0.0233

JW5846	*ygeO*	Pseudogene, orga family, part of T3SS PAI ETT2 remnant	-0.0129	0.6515
JW5847	*prfB*	Peptide chain release factor RF-2	-0.0140	0.5465
JW5848	*yghO*	Pseudogene, IS-interrupted	-0.0388	0.3535
JW5849	*yqhC*	Transcriptional activator of yqhd	-0.0203	0.8237
JW5850	*yqjF*	Putative quinol oxidase subunit, trinitrotoluene-inducible	0.0257	0.4305
JW5851	*pnp*	Polynucleotide phosphorylase/polyadenylase	0.0627	0.2491
JW5852	*yihO*	Putative sulphoquinovose importer	0.0883	0.3056
JW5853	*ysgA*	Putative carboxymethylenebutenolidase	0.0350	0.6245
JW5854	*yigL*	Pyridoxal phosphate phosphatase	-0.0029	0.9034
JW5855	*recQ*	ATP-dependent DNA helicase	-0.0228	0.5800
JW5856	*trxA*	Thioredoxin 1	0.0057	0.8968
JW5857	*rbsD*	D-ribose pyranase	-0.0251	0.7557
JW5858	*yidX*	Putative lipoprotein	0.0255	0.1966
JW5859	*dgoT*	D-galactonate transporter	0.0793	0.0064
JW5860	*yidR*	DUF3748 family protein	0.0354	0.2498
JW5864	*yrhA*	Pseudogene, interrupted by IS1E	-0.0396	0.2355
JW5865	*yrfG*	GMP/IMP nucleotidase	0.0596	0.0986
JW5867	*aidB*	DNA alkylation damage repair protein; flavin-containing DNA binding protein, weak isovaleryl coa dehydrogenase	-0.0160	0.5958
JW5868	*ulaG*	L-ascorbate 6-phosphate lactonase	-0.0349	0.1907
JW5869	*yjiK*	Sdia-regulated family putative membrane-anchored protein; putative phytase-like esterase	-0.0426	0.2885
JW5871	*mcrB*	5-methylcytosine-specific restriction enzyme mcrbc, subunit mcrb	0.0755	0.1938
JW5873	*prfC*	Peptide chain release factor RF-3	0.1115	0.0346
JW5874	*ydhM*	Transcriptional repressor for the nemra-gloa operon, quinone-, glyoxal-, and hocl-activated	-0.0358	0.4051
JW5875	*nuoB*	NADH:ubiquinone oxidoreductase, chain B	0.0215	0.6863
JW5876	*yfeH*	Putative inorganic ion transporter	0.0250	0.3265
JW5877	*ypeB*	DUF3820 family protein	-0.0569	0.0884
JW5878	*csiE*	Stationary phase inducible protein	0.0287	0.4991
JW5892	*yadB*	Glutamyl-Q trna(Asp) synthetase	0.0659	0.0875
JW5893	*yahH*	Uncharacterized protein	0.0658	0.0978
JW5894	*cynR*	Transcriptional activator of cyn operon; autorepressor	0.1116	0.1371
JW5896	*ybhD*	Putative DNA-binding transcriptional regulator	-0.0443	0.1941
JW5897	*yliA*	Glutathione ABC transporter atpase	0.0256	0.4091
JW5898	*yccW*	23S rrna m(5)C1962 methyltransferase, SAM-dependent	-0.0130	0.7210
JW5901	*ycgH*	CP4-44 prophage; putative disrupted hemin or colicin receptor	-0.0866	0.0483
JW5904	*lomR*	CP4-44 prophage; putative disrupted hemin or colicin receptor	-0.0904	0.0185
JW5905	*hrpA*	Putative ATP-dependent helicase	-0.0262	0.4981

JW5906	*gapC*	CP4-44 prophage; putative disrupted hemin or colicin receptor	0.0327	0.1909
JW5907	*yncB*	Curcumin/dihydrocurcumin reductase, NADPH-dependent	-0.0135	0.7316
JW5908	*yddM*	Putative DNA-binding transcriptional regulator	0.3552	0.0393
JW5909	*ydfU*	Qin prophage; DUF968 family protein	-0.0022	0.9454
JW5910	*ydiD*	Medium-chain fatty-acid--coa ligase	0.0562	0.5272
JW5911	*yniD*	Uncharacterized protein	-0.0187	0.4682
JW5912	*yedN*	CP4-44 prophage; putative disrupted hemin or colicin receptor	0.0172	0.6704
JW5913	*intG*	CP4-44 prophage; putative disrupted hemin or colicin receptor	0.0109	0.8156
JW5915	*molR*	CP4-44 prophage; putative disrupted hemin or colicin receptor	0.0856	0.0105
JW5916	*molR*	CP4-44 prophage; putative disrupted hemin or colicin receptor	-0.0014	0.9648
JW5917	*rcsC*	CP4-44 prophage; putative disrupted hemin or colicin receptor	0.0131	0.7198
JW5921	*yfjS*	CP4-57 prophage; uncharacterized protein	-0.0015	0.9601
JW5923	*ygfK*	Putative Fe-S subunit oxidoreductase subunit	0.0430	0.0413
JW5924	*yghE*	CP4-44 prophage; putative disrupted hemin or colicin receptor	0.0846	0.0074
JW5925	*yghJ*	Putative secreted and surface-associated lipoprotein mucinase	-0.0534	0.1289
JW5926	*yghX*	CP4-44 prophage; putative disrupted hemin or colicin receptor	-0.0449	0.1164
JW5927	*ygiB*	DUF1190 family protein	0.0178	0.3741
JW5929	*yiiE*	Copg family putative transcriptional regulator	-0.0302	0.4433
JW5931	*tatD*	Quality control of Tat-exported fes proteins; Mg-dependent cytoplasmic dnase	-0.0690	0.0022
JW5937	*ysdC*	CP4-44 prophage; putative disrupted hemin or colicin receptor	0.0291	0.3287
JW5938	*yicM*	Putative transporter	0.0871	0.1302
JW5939	*yicJ*	Putative transporter	-0.0103	0.6325
JW5940	*bisC*	Biotin sulfoxide reductase	0.0872	0.0004
JW5941	*yhjQ*	CP4-44 prophage; putative disrupted hemin or colicin receptor	0.0393	0.3432
JW5942	*bcsC*	Cellulose synthase subunit	-0.0482	0.1226
JW5943	*yhjK*	Cyclic-di-GMP phosphodiesterase	0.0355	0.3838
JW5944	*yhiM*	Acid resistance protein, inner membrane	0.0090	0.5994
JW5945	*yhhS*	Putative arabinose efflux transporter	0.0026	0.9352
JW5946	*gntR*	D-gluconate inducible gluconate regulon transcriptional repressor	-0.0340	0.2597
JW5947	*aroK*	Shikimate kinase 1	-0.0126	0.5728
JW5948	*yhfZ*	Putative DNA-binding transcriptional regulator	0.0444	0.0570
JW5949	*yrdD*	Ssdna-binding protein, function unknown	-0.0178	0.5967
JW5950	*yjbN*	Trna-dihydrouridine synthase A	0.0209	0.5231

JW5952	*yjhU*	Putative DNA-binding transcriptional regulator; kple2 phage-like element	-0.0440	0.0653
JW5953	*yjiP*	CP4-44 prophage; putative disrupted hemin or colicin receptor	0.0538	0.1816
JW5954	*yjiV*	CP4-44 prophage; putative disrupted hemin or colicin receptor	0.0078	0.6908
JW5955	*bglJ*	Bgl operon transcriptional activator	-0.0974	0.0315
JW5956	*ykfH*	Uncharacterized protein	-0.0155	0.6173
JW5960	*ymjC*	Pseudogene	-0.0084	0.7474
JW5962	*sra*	Stationary-phase-induced ribosome-associated protein	0.0340	0.5359
JW5963	*blr*	Beta-lactam resistance membrane protein; divisome-associated protein	-0.0275	0.4834
JW5964	*ypaA*	CP4-44 prophage; putative disrupted hemin or colicin receptor	-0.0503	0.2689
JW5965	*yicS*	Putative periplasmic protein	0.0290	0.3327
JW5967	*sgcB*	Putative enzyme IIB component of PTS	0.1681	0.0004
JW5968	*yjhX*	UPF0386 family protein	-0.0607	0.3753
JW5970	*yccV*	Heat shock protein hspq	-0.0345	0.3263
